# Automated seizure onset zone locator from resting-state functional MRI in drug-resistant epilepsy

**DOI:** 10.3389/fnimg.2022.1007668

**Published:** 2023-01-04

**Authors:** Ayan Banerjee, Payal Kamboj, Sarah N. Wyckoff, Bethany L. Sussman, Sandeep K. S. Gupta, Varina L. Boerwinkle

**Affiliations:** ^1^School of Computing and Augmented Intelligence, Arizona State University, Tempe, AZ, United States; ^2^Division of Neuroscience, Barrow Neurological Institute at Phoenix Children's Hospital, Phoenix, AZ, United States; ^3^Division of Child Neurology, University of North Carolina Department of Neurology, Chapel Hill, NC, United States

**Keywords:** resting state fMRI, seizure onset zone, resting state network, drug resistant epilepsy, expert knowledge driven classification

## Abstract

**Objective:**

Accurate localization of a seizure onset zone (SOZ) from independent components (IC) of resting-state functional magnetic resonance imaging (rs-fMRI) improves surgical outcomes in children with drug-resistant epilepsy (DRE). Automated IC sorting has limited success in identifying SOZ localizing ICs in adult normal rs-fMRI or uncategorized epilepsy. Children face unique challenges due to the developing brain and its associated surgical risks. This study proposes a novel SOZ localization algorithm (EPIK) for children with DRE.

**Methods:**

EPIK is developed in a phased approach, where fMRI noise-related biomarkers are used through high-fidelity image processing techniques to eliminate noise ICs. Then, the SOZ markers are used through a maximum likelihood-based classifier to determine SOZ localizing ICs. The performance of EPIK was evaluated on a unique pediatric DRE dataset (*n* = 52). A total of 24 children underwent surgical resection or ablation of an rs-fMRI identified SOZ, concurrently evaluated with an EEG and anatomical MRI. Two state-of-art techniques were used for comparison: (a) least squares support-vector machine and (b) convolutional neural networks. The performance was benchmarked against expert IC sorting and Engel outcomes for surgical SOZ resection or ablation. The analysis was stratified across age and sex.

**Results:**

EPIK outperformed state-of-art techniques for SOZ localizing IC identification with a mean accuracy of 84.7% (4% higher), a precision of 74.1% (22% higher), a specificity of 81.9% (3.2% higher), and a sensitivity of 88.6% (16.5% higher). EPIK showed consistent performance across age and sex with the best performance in those < 5 years of age. It helped achieve a ~5-fold reduction in the number of ICs to be potentially analyzed during pre-surgical screening.

**Significance:**

Automated SOZ localization from rs-fMRI, validated against surgical outcomes, indicates the potential for clinical feasibility. It eliminates the need for expert sorting, outperforms prior automated methods, and is consistent across age and sex.

## Introduction

Epilepsy is devastating, affecting 50 million people worldwide (WHO). One in 150 children have epilepsy (Aaberg et al., 2017; Epilepsy Foundation, 2018), with 30% having drug-resistant epilepsy (DRE; Wieser et al., 2001; Kwan and Sander, 2004; Kwan and Brodie, 2010), which causes significant morbidity and mortality (Sillanpää and Shinnar, 2010; Laxer et al., 2014; Engel, 2016). A consensus proposal by the *ad-hoc* Task Force of the International League Against Epilepsy (ILAE) proposed the following definition for DRE: “a failure of adequate trials of two tolerated, appropriately chosen, and used antiepileptic drug schedules (whether as monotherapies or in combination) to achieve sustained seizure freedom (considered as freedom from all seizures, including auras) for at least 12 months” (Kwan et al., 2010).

Early diagnosis and treatment of DRE can potentially deflect complications such as evolution into status epilepticus (Prisco et al., 2020) and Sudden Unexplained Death in Epilepsy (SUDEP), wherein the individual dies due to cardio-respiratory failure from presumed nocturnal seizure activity (Sillanpää and Shinnar, 2010). Moreover, in children, timely diagnosis, intensive management, and treatment are pivotal in minimizing neurological damage (Prisco et al., 2020). Further, the earliest onset of severe epilepsy in the neonatal population can lead to nearly constant life-threatening seizures requiring an urgent need for surgical evaluation early in life (Russ et al., 2021).

### Surgery for DRE

The most effective treatment for DRE is surgery (Luders et al., 2006; Luckett et al., 2022). Early surgery is key: “minimally invasive surgical treatment can be a life-changing option for DRE patients; hence management of the SOZ requiring disconnecting techniques (Young et al., 2020), or deep sited lesions requiring excision should be considered earlier rather than later (Chibbaro et al., 2017).” Notably, recent findings showed that ultra-early (before 3 months old) surgical intervention in children evaluated to have DRE after trials of an average of four anti-seizure drugs, although seldom performed, has excellent epilepsy outcomes and leads to a decrease in usage of anti-seizure drugs, without any increased risk of surgery-related permanent morbidity (Roth et al., 2021).

### Brain imaging for pre-surgical screening

Surgical intervention in DRE requires accurate localization of the seizure onset zone (SOZ) for success. We make a distinction between the epileptic network (EN) and the SOZ. The EN denotes regions where seizure propagates and may be more extensive than the SOZ. As such, it may be difficult as well as unnecessary to surgically eliminate the EN since it can incorporate sensitive areas of the brain. Several brain imaging techniques have been explored to identify the ictal seizure onset zone, propagation zone (i.e., EN), and interictal activity (Table 1). This can be done with nuclear medicine-based imaging techniques such as positron emission tomography (PET) or ictal single-photon emission computerized tomography (SPECT; Desai et al., 2013). Recent studies suggest some SOZ identification capabilities for PET and SPECT in both adults and children; however, their accuracy heavily depends on the timing of the scan. Delay in drug infusion can result in the detection of the EN instead of the SOZ. Invasive modalities such as intracranial EEG (iEEG) are considered the gold standard for SOZ identification and have shown excellent accuracy for both adults and pediatric DRE. Stereo-electroencephalography (SEEG) is minimally invasive, uses a three-dimensional configuration of depth electrodes to localize epileptiform activity, and has shown some SOZ identification capability recently (Satzer et al., 2022).

**Table 1:** Summary of brain imaging techniques and their application in SOZ identification for adults and children.

**Imaging technique**	**Invasive/non-invasive**	**Number of imaging sessions**	**Spatial resolution**	**Temporal resolution**	**Brain area**	**SOZ ID in adults with DRE**	**SOZ ID in children with DRE**
PET	Invasive	1	6 mm	5 mins	Surface	Identified in 66.7% of subjects (Mayoral et al., 2019)	Only detects EN in 6.6% of patients and not SOZ (Bansal et al., 2016)
Ictal SPECT	Invasive	2, needs difference between ictal and inter-ictal SPECT (Desai et al., 2013)	6 mm	15 mins	Surface	Detects EN and not SOZ (Kaiboriboon et al., 2002; von Oertzen, 2018)	Detects EN and not SOZ (Van Paesschen et al., 2007)
MEG	Non-invasive	1	10–20 mm	<10 ms	Cortex	75% accurate (Foley et al., 2021) in eight patients, 60% with ML in a large cohort (Nissen et al., 2018)	Poor concordance with SOZ. Identified onset zone is >20 mm distance from ground truth SOZ (Ntolkeras et al., 2022)
iEEG (gold standard)	Invasive	Long-term monitoring (14 days)	6–8 cm	<5 ms	Cortex	92.3% accurate (Nagahama et al., 2018)	>90% accurate (Nagahama et al., 2018)
fMRI	Non-invasive	1	3 mm	2–5s	Central brain	Sensitivity 83% and specificity 67% (Chen et al., 2017)	89% accurate SOZ identification (Boerwinkle et al., 2019)

PET, positron emission tomography; SPECT, single-photon emission computerized tomography; MEG, magnetoencephalography; iEEG, intracranial electroencephalography; fMRI, functional magnetic resonance imaging.

However, traditional analysis of PET, SPECT, or SEEG is relatively, temporally, and spatially restricted, whereas functional interpolation of brain activity might allow for a non-invasive three-dimensional representation of epileptiform activity and avoid pitfalls inherent of other modalities (Table 1). Recently, magnetoencephalography (MEG) and functional magnetic resonance imaging (fMRI)-based non-invasive techniques have been analyzed for DRE in both adults and children and show decent SOZ identification capability. A combination of MEG and fMRI imaging has also been proposed for accurate SOZ identification (Berger et al., 2021). However, a major drawback of such brain imaging-based SOZ identification techniques is the heavy reliance on manual sorting of images and their components, which not only increases cost but also reduces accessibility and repeatability.

Unfortunately, <1% of patients with DRE are evaluated for surgery and only 25% of those undergo surgery (Engel, 2016), partly due to the high cost of diagnostic and surgical treatment (>$200,000/patient) and the risk of debilitating impairment (Murray et al., 1996; Begley et al., 2000). Of the 1% evaluated, surgical failure rates are 30–70% despite the use of non-invasive SOZ-localization biomarkers such as anatomical MRI, scalp EEG, simultaneous EEG-fMRI, and magnetoencephalography, which are then often confirmed by invasive iEEG (McIntosh et al., 2004; Luders et al., 2006; Sillanpää and Shinnar, 2010; Bulacio et al., 2012; Laxer et al., 2014; Engel, 2016; Epilepsy Foundation, 2018). Hence, for surgery to be safe and efficient for wide acceptance (England et al., 2012), accessible, minimally invasive, and accurate SOZ localization is essential.

One of the newer methods showing promise, to this end, is resting-state functional MRI (rs-fMRI). Rs-fMRI has been shown to have an accurate SOZ-localization capacity through various analysis approaches (Bandt et al., 2014; van Houdt et al., 2015; Malmgren and Edelvik, 2017; Boerwinkle et al., 2018), but only independent component analysis (ICA; Gonzalez-Martinez et al., 2007) has provided Level 1 evidence and has led to improvement in surgical outcomes (Malmgren and Edelvik, 2017; Chakraborty et al., 2020) and candidacy (Boerwinkle et al., 2019) in DRE. However, expert interpretation of independent components (IC) into sources of noise, normal resting state networks (RSN), and SOZs (Hunyadi et al., 2014; Boerwinkle et al., 2017, 2020) limits reproducibility and availability. An automated whole-brain data-driven SOZ-localizing IC identification technique that is rigorously validated against surgical destruction outcomes, reproducible, equally effective across age and sex, and applicable to all epilepsy subtypes may greatly improve epilepsy care feasibility, morbidity, and mortality.

### fMRI-based screening

**Figure 1:**
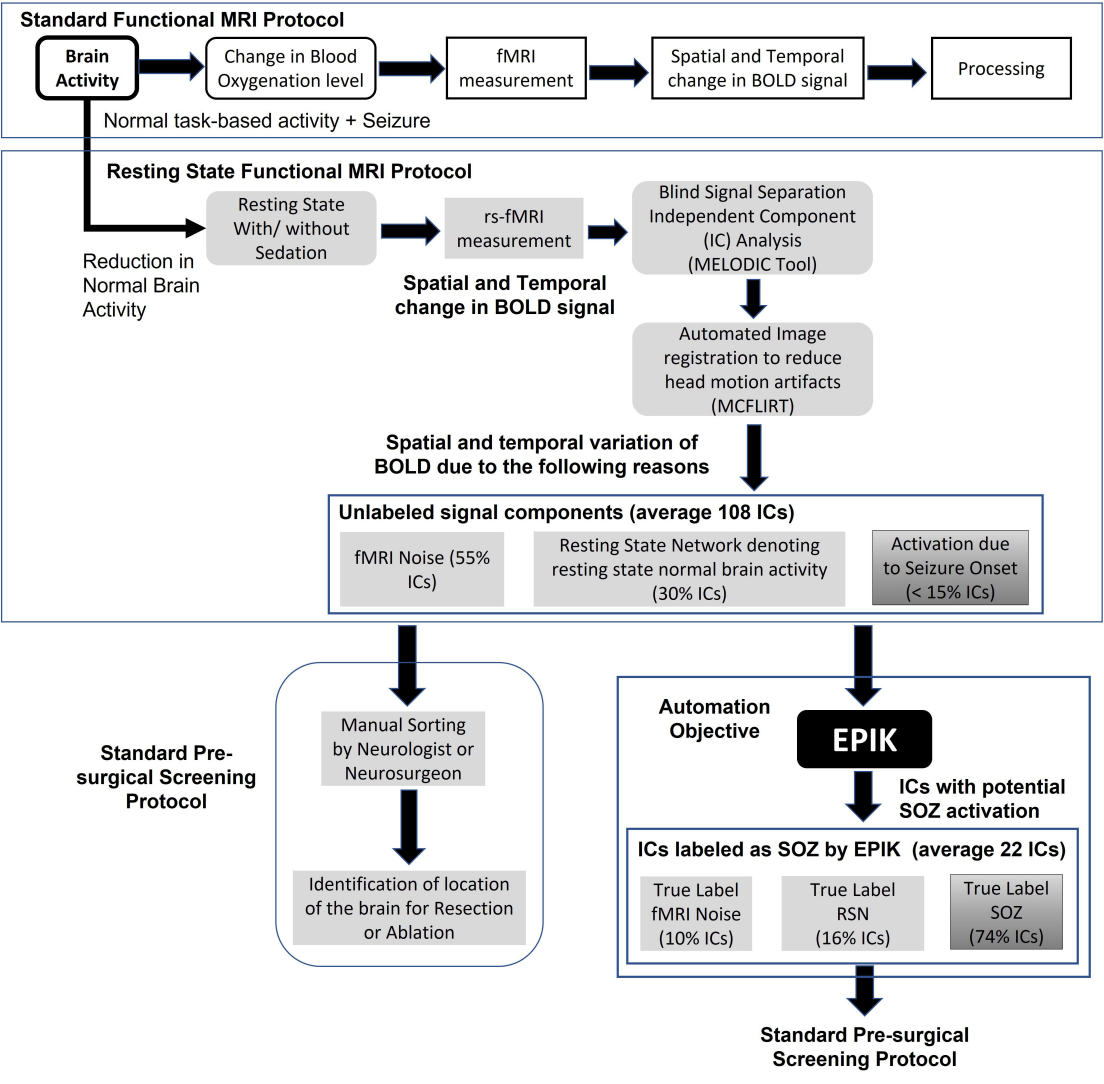
The standard task-based fMRI protocol (top panel), resting state fMRI and data processing pipeline (middle panel), standard rs-fMRI-based pre-surgical evaluation of SOZ location (lower left panel), automation objective of EPIK (lower right panel), and advantages of EPIK in terms of reduction in manual processing requirements.

Functional MRI (fMRI) is a popular imaging technique originally used to identify brain activity in terms of blood oxygenation level change in different parts of the brain for a given mental task (Figure 1). However, for SOZ detection, it is required to identify blood oxygenation changes due to the onset of seizure. Hence, an important step is to remove other sources of brain activity such as mental tasks, fMRI noise, and head motion. Rs-fMRI requires the subject to be in a resting state, which is achieved in a majority of children through sedation. Even if any mental task is eliminated, there is still the presence of resting-state brain activity in subjects, which manifests as RSN brain activity. Head motion is a significant source of noise. Even if head motion is limited to <1 mm, it still can pose a significant amount of noise in the rs-fMRI measurement. Automated image registration is used to reduce head motion artifacts in rs-fMRI (Figure 1 middle panel). The resulting rs-fMRI captures brain activity due to several sources including (a) noise (fMRI machine noise and head motion), (b) RSN (resting-state activity of the brain), and (c) SOZ (change in blood oxygenation due to seizure onset). To decouple the effects of noise, RSN, and SOZ in rs-fMRI signals, ICA is used to recover mutually independent fMRI signal components (ICs) that potentially only capture brain activity from one of the three sources.

Rs-fMRI ICA results in ~100 ICs. Each IC is a spatial-temporal distribution of regions of synchronous activity. In ICA of those with DRE, there are three IC categories: (1) RSNs which are well-described and validated in the literature; (2) SOZ which is, currently, highly dependent on expert sorting; and (3) noise, which is also well-understood, resulting from cardiovascular, cerebral-spinal-fluid-pulsation, or scanner artifacts [see Boerwinkle et al. (2017) for details and examples]. In standard rs-fMRI-based pre-surgical screening for children with DRE, the entire set of ICs is analyzed by a neurosurgeon or neurologist to determine which ICs capture blood oxygenation changes due to seizure onset. Such ICs are referred to as SOZ localizing IC. The neurosurgeon then determines the location of seizure onset in the brain using the SOZ localizing IC and a recommendation for a surgical procedure such as resection or ablation or neurostimulation is made.

Given that ICA results in > 100 ICs and only < 10% are SOZ localizing ICs, manual sorting of rs-fMRI ICs to search for SOZ localizing IC is a significant time commitment by the neurosurgeon, resulting in increased cost, reduced availability, and a higher chance of false positives (Figure 1). This study focuses on automating the task of IC sorting and reducing the number of ICs to be analyzed by the neurosurgeon for pre-surgical evaluation for children with DRE.

### Automation of fMRI-based screening

Artificial Intelligence (AI) has been employed on rs-fMRI to automatically identify several brain disorders including Attention Deficit Hyperactivity Disorder (ADHD), Alzheimer's disease, White Matter Hyperintensity (WMH; Bharath et al., 2019), and major depressive disorder (Nguyen et al., 2021). Recent studies considered two automation objectives in epilepsy with rs-fMRI (Table 2): (a) classification of subjects with or without epilepsy by identifying epilepsy networks using rs-fMRI blood oxygen level-dependent (BOLD) signal *z*-score latency maps (Lopes et al., 2012; Bharath et al., 2019; Nguyen et al., 2021), and (b) localization of the seizure onset zone using rs-fMRI ICs (Hunyadi et al., 2014, 2015; Shah et al., 2019). Epilepsy networks indicate the areas of the brain that are affected by the propagation of a seizure. As such, they may not indicate the origin of the seizure, which is encapsulated by the SOZ. Our research focus in this study tackles the second automation objective of SOZ localization.

**Table 2:** Comparison of related research highlighting innovative aspects of the proposed research.

**Problem**	**References**	**Epilepsy type**	**Epileptic zone indicators**	**Task objective**	**Validation modality**	**Machine learning method**	**Supervised (sup)/ unsupervised (U)**	**Performance**	***N***	**A adult C child**	***N* of epilepsy Subjects (*N* with DRE)**	***N* of test epilepsy subjects**	***N* of subjects with surgical outcomes**
Detection of epilepsy	Nguyen et al., 2021	DRE	Epilepsy Network (EN)	Epilepsy classification using fMRI *z* score latency	Seizures	CNN	Sup	Accuracy in Epilepsy identification = 74% Sensitivity = 85% Specificity = 71%	322	C	63 (63)	13	0
	Lopes et al., 2012	Focal	Not Specified (NS)	Epilepsy classification using BOLD time series	Seizures	Time series analysis	U	Accuracy = 87.5%	15	A	15 (0)	15 subjects with 40 events	0
	Bharath et al., 2019	Focal Temporal Lobe	Hand classification EN	Epilepsy classification using ICA	Seizures	SVM	Sup	Accuracy = 97.5% Sensitivity = 100% Specificity = 94.4%	132	A	42 (0)	0 (No test data, cross validation accuracy)	0
Pre-surgical screening to determine seizure onset zone (SOZ)	Boerwinkle et al., 2017	DRE	RSN and SOZ	Manual SOZ localization	iEEG and post-op seizure	No automation	Sup	89% accuracy	40	C	40 (40)	33	40
	Shah et al., 2018	DRE	NS	Finding correlation between fMRI *z* score latency and seizure freedom	Post-op seizure	Statistical correlation measures	U	25 out of 26 subjects have temporal lobe signal latency	26	C	26 (26)	26	26 (21 seizure free)
	Shah et al., 2019	DRE	NS	fMRI *z* score latency-based seizure foci lateralization	Manual lateralization	Statistical correlation measures	U	Mean accuracy 70% Mean sensitivity 85% Mean Specificity 65%	38	C	38 (38)	38	38 (14 seizure free)
	Hunyadi et al., 2015	DRE	SOZ	Automated SOZ identification (ID)	EEG-fMRI	LS-SVM	Sup	40% sensitivity 77% specificity	18	A	18 (18)	10	Not Specified (NS)
	Zhang et al., 2015	DRE	SOZ	SOZ localization with the manual determination of brain boundary	Concordance with surgery resection	Statistical methods using thresholds	U	77.7% sensitivity^*^ 57% specificity^**^	9	A	9 (9)	9	NS
	Lee et al., 2014	Intractable partial	SOZ	VDC-based SOZ localization	iEEG.	Statistical methods on time series	U	72.4% sensitivity^***^	29	A	29 (29)	21	29 (2 seizure free)
	Nozais et al., 2021	Healthy	RSN	DL-based RSN ID	Manual IC Sorting	MLP	Sup	92% accuracy	2000	A	0 (0)	0	0
	Luckett et al., 2022	Focal Temporal Lobe	RSN and SOZ	DL-based SOZ hemisphere ID	Hemisphere lateralization of SOZ, and RSNs	CNN	Sup	90.6% accuracy^*****^	2164	A	32 (0)	32	15 (11 seizure free)
	EPIK (current study)	DRE	RSN and SOZ	Fully automated unsupervised method (EPIK)	Manual IC sorting Comparison of Hunyadi et al. and Nozais et al. on the same dataset; and post-op seizures	Rule guided noise elimination, maximum likelihood based classification	U	RSN success: Specificity: 73.7% Sensitivity: 72% SOZ success: Specificity: 81.9% Sensitivity: 88.6%	52	C	52 (52)	52	24 (18 seizure free)

DL, deep learning; DRE, drug-resistant or intractable epilepsy; EEG-fMRI, simultaneous EEG and functional MRI; IC, independent component; ID, identification; iEEG, intracranial EEG; ML, machine learning; LS-SVM, least squares support vector machine; VDC, voxel degree connectivity; CNN, convolutional neural network.

* Zhang et al. (2015) only mentions concordance with surgical resection. Concordance is assumed to be true positive, and failure is assumed to be false negative. Hence percentage concordance is assumed to be sensitivity.

** Zhang et al. (2015) mentions success in rejecting non-epilepsy related IC. Rejection of non-epilepsy IC is assumed to be true negative and failure to reject is assumed to be false positive. Hence success rate is specificity.

*** Lee et al. (2014) defines accordance with IC EEG. Accordance is assumed to be true positive, and failure is assumed to be false negative. Hence, percentage accordance is assumed to be sensitivity.

***** Sensitivity and specificity not mentioned. The high accuracy could also be due to the presence of a large number of true negatives.

T epileptic networks were those found to be altered compared to healthy controls, however, were not identified as being causative of epilepsy or a seizure onset zone.

Automated classification of rs-fMRI ICs as SOZ or RSN has been explored using supervised shallow machine learning (ML; Nozais et al., 2021) and using deep learning (DL) in healthy adults to identify the typical RSNs and is yet to be tested in epilepsy (Zhang et al., 2019; Table 2). Supervised ML indicates that the DRE population has to be divided into two parts: (a) a training set, which is used to configure the ML, and (b) a testing set, which is used to test the performance of the ML. Some supervised ML can also choose to utilize a validation set as mentioned in a previous study (Nguyen et al., 2021). The performance of the ML technique on the validation set is used to update the training process and improve the performance in the validation set. Hence, the performance on the validation set is excluded from the analysis in Table 2 and only the test set performance is reported. Recent automated (Luckett et al., 2022) methods to classify adult rs-fMRI into RSN, SOZ, and noise ICs are of three types: (1) voxel-based network measures quantifying the number of connections to each voxel in an IC, called voxel degree connectivity (VDC), as indicators for SOZ (Hunyadi et al., 2014; Lee et al., 2014). Such approaches have a small sample size (*n* ≈ 20) and show a maximum reported sensitivity of 77% and a specificity of 57% (Table 2); (2) ML-based classification, with a sensitivity of 40% and a specificity of 77% (Hunyadi et al., 2014); and (3) DL approaches for only identifying RSN and noise, but not SOZs, for normal and non-DRE patients with epilepsy [accuracy 92% (Nozais et al., 2021) in Table 2].

To date, automated approaches have not been successful in the classification of RSN, noise, and SOZ, in rs-fMRI for pediatric patients with DRE due to the following challenges: (1) Lack of normalized pathological rs-fMRI RSN data for children (Zhang et al., 2019); (2) databases with balanced instances of RSN, SOZ, and noise, large enough for DL techniques to effectively recognize the three IC categories that are not available; (3) the potentially inadequate performance of SOZ identification in children with DRE can indicate a high risk of developmental disorders post-surgery. Given that each patient only has 5% ICs as SOZ, a 40% sensitivity (Hunyadi et al., 2014) indicates that only two out of five SOZ ICs are correctly identified but 14 of them are wrongly identified as SOZ; and (4) fMRI-based pre-surgical mapping is more complicated for children with DRE due to developmental changes during cognitive maturation (Jiang et al., 2018; Bouyssi-Kobar et al., 2019), the impairment experienced due to DRE, and the normal representation of memory function during development (Michels et al., 2012; Darki and Klingberg, 2015; Cui et al., 2018; Kasradze et al., 2021), which may differ from adults (Faghiri et al., 2017; Lee et al., 2019; DeGeorge et al., 2021; Moncrief et al., 2021). Hence, the efficacy of fMRI classification techniques on adults needs to be reexamined for children with DRE.

Most current studies (Table 2) focused on adult epilepsy with an unknown effect of the degree of hypothesized network disruption effect on localization. Currently available automated IC sorting techniques either only identify SOZ or RSN localizing ICs. Hunyadi et al. (2015), the first major work to attempt SOZ localizing ICs identification, used supervised ML but could only achieve a specificity of 77% and a sensitivity of 40% on a subset of the adult patient population. A more recent technique by Nozais et al. (2021) used DL to identify only RSN in healthy adults and reports an accuracy of 92%. The major drawback of DL techniques is the requirement for labeled data on all three IC categories. Table 2 shows that such labeled data is rarely available, even if we combine datasets from different authors, IC data labeled as RSN and SOZ are only available from 212 children with DRE. For DL to successfully recognize SOZ, it will need at least a balanced distribution of RSN and SOZ. The DL works in this domain utilize RSN data from 2,000 healthy subjects for appropriate training (Table 2; Nozais et al., 2021; Luckett et al., 2022). Hence, to achieve balanced data, we would need SOZ from at least 2,000 subjects, a sample size that is currently not available.

There has been one prior unsupervised approach by Zhang et al. (2015); however, it was applied to DRE adults and achieved a sensitivity of 78% and a specificity of 57%. We cannot replicate that study for this paper, because specific information about parameter settings was not discussed in Zhang et al. (2015).

### The difference between EPIK and supervised ML

**Figure 2:**
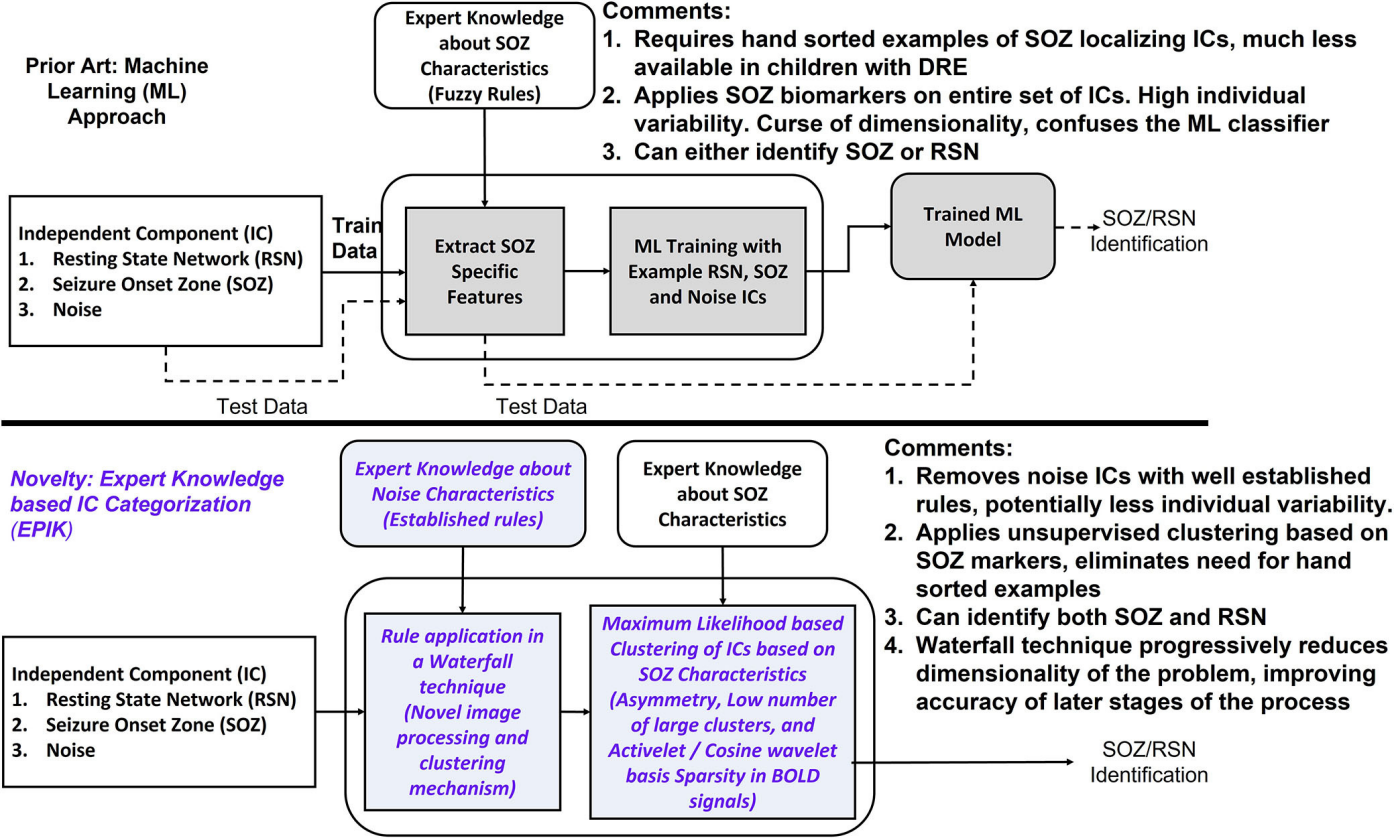
Innovations in EPIK. Compared to prior ML techniques, EPIK first purges ICs with noise markers by employing rules compiled from experts. The ICs that pass the initial purge, are then classified into RSN and SOZ based on the maximum likelihood-based clustering mechanism. The italicized text marks the innovations in this work.

In this study, we present a novel, unsupervised technique to identify SOZ localizing ICs that require no prior dataset for training and classify ICs by encoding expert knowledge. The unsupervised nature of our algorithm implies that the entire dataset is used as a test set and no training dataset is required. Our algorithm is tested on the largest number of children with DRE among the recent studies on automated SOZ identification mechanisms with rs-fMRI listed in Table 2. Figure 2 illustrates differences from Figure 1. ML techniques (Figure 2) utilize examples of SOZ and RSN ICs to learn a model in the training phase, which is subsequently used for the identification of SOZ on previously unseen rs-fMRI signals. Such techniques have not been successful, possibly for the following reasons: (1) SOZ biomarkers are not precise and exhibit significant individual variances (Hunyadi et al., 2015; Boerwinkle et al., 2017) and (2) patients have low numbers of SOZ localizing ICs as compared to noise and RSN, leading to an imbalance in data and potential overfitting of ML models.

In the current study, EPIK (ExPert Knowledge-based IC categorization; Figure 2) is used with an alternative approach. Instead of directly learning SOZ-related features from training data, EPIK first used expert rules in a waterfall technique to purge noise ICs. Noise markers used by EPIK such as clusters outside brain boundaries or overlapping white matter or arteries are well-established, evidenced by consistency across several publications (Kelly et al., 2010; Griffanti et al., 2014, 2017). It then used SOZ-specific spatial and temporal markers in a maximum likelihood-based clustering to further classify the ICs into RSN and SOZ. Clustering was unsupervised and did not implement training with prior data to tune its parameters.

To illustrate differences compared to prior, we replicated the shallow learning strategy of Hunyadi et al. (2014) and implemented a Convolution Neural Network (CNN) based DL technique (Krizhevsky et al., 2012; Cui et al., 2018; Nozais et al., 2021) for the identification of SOZ localizing ICs from rs-fMRI, thereby providing a preliminary comparative study of all three approaches on the same dataset of children with DRE using the standard metrics of accuracy, precision, specificity, and sensitivity. We hypothesize that EPIK will perform at least equally well as prior methods and consistently across age and sex, due to being informed by developmental- and sex-informed expert sorting in the pediatric DRE population.

## Materials and methods

### Inclusion criteria

Patients who were determined to have DRE by a treating epileptologist and received surgery evaluation were included. Most of the patients had focal epilepsy; however, rapid generalization of epileptiform activity from an epileptogenic focus may appear to be generalized epilepsy when evaluated using surface EEG. Hence, generalized epilepsy was not an exclusion criterion.

### Data collection method

**Figure 3:**
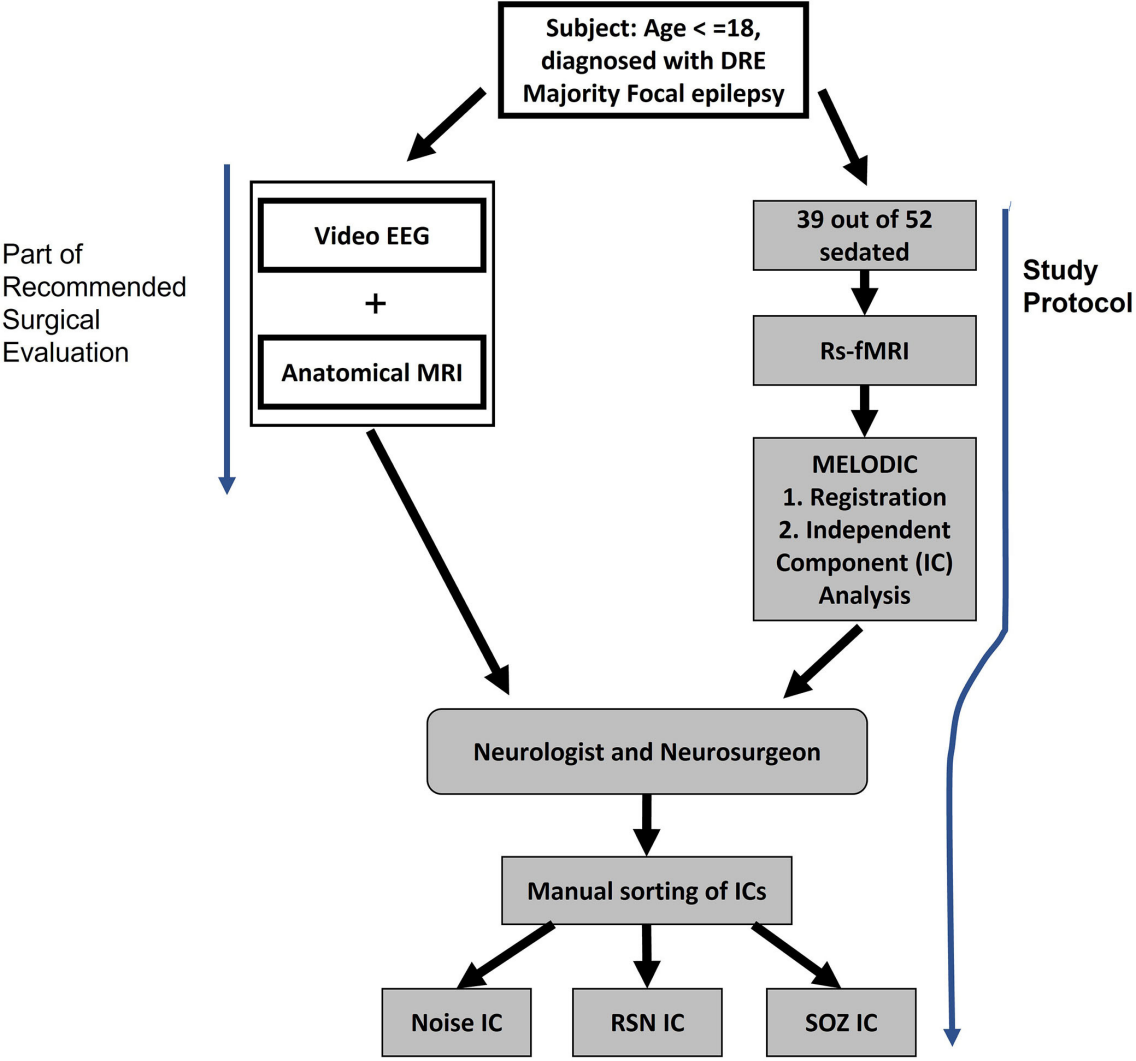
Study procedure for extracting noise, RSN, and SOZ localizing ICs. Subjects underwent video EEG and anatomical MRI as a part of the normal pre-surgical evaluation. In addition, the study protocol was also administered where rs-fMRI was collected.

The rs-fMRI data from 52 children with DRE aged 3 months−18 years old, who were under the care of a treating epileptologist at Phoenix Children's Hospital (PCH), were selected in descending alphabetical order from the PCH clinical database (Age and sex distribution provided in Table 3). The diagnosis of DRE was according to the treating epileptologist's documented medical record notes. The children received rs-fMRI, video EEG, and anatomical MRI as part of standard clinical MRI SOZ localization for epilepsy surgery evaluation (Figure 3). For rs-fMRI, patients who were determined to require conscious sedation, received a propofol infusion as a part of standard care determined by the institution's policies. Of the 52 children, 39 required conscious sedation. The dataset included patients who had <1 mm head motion in any direction during scanning. For children who received sedation, propofol administered at levels to produce conscious sedation (80–110 micrograms/kilogram/minute), avoiding higher dosages typical of general anesthesia, was utilized. Propofol administered at levels producing conscious sedation reduces the BOLD signal strength by ~10%, still allowing for complete network detection (Vanderby et al., 2010; Schrouff et al., 2011). General anesthesia causes gross loss of ability to detect the large-scale cortical networks and, was, therefore avoided.

**Table 3:** Patient distribution and information about the data set.

Number of subjects	52
Age ≤ 5 years	20
Age > 5 and ≤ 13	18
Age > 13 and ≤ 18	14
Men/Women	23/29
Prior surgery	2
Surgery post resting-state fMRI	24 (ablation 15, resection 7, disconnection 2)
Seizure free post-surgery, and rs-fMRI SOZ is the same location as the region destroyed determined by expert review of pre-operative rs-fMRI SOZ and post-operative imaging	16 (ablation 10, resection 6, disconnection 1)

As part of the standard of care, the children also received inpatient video EEG and anatomical MRI. This data also aided the manual identification of SOZ localizing ICs in rs-fMRI (Figure 3).

The MRI images were acquired using a 3T MRI unit from Ingenuity Philips Medical systems. It has a 32-channel head coil. The resting state fMRI parameters were set at TR 2,000 ms, TE 30 ms, matrix size 80 × 80, flip angle 80, number of slices 46, slice thickness 3.4 mm with no gap, in-plane resolution 3 × 3 mm, interleaved acquisition, and number of total volumes 600, in two 10-min runs, with a total time of 20 mins.

### rs-fMRI pre-processing

Oxford Centre FMRIB (Functional MRI of the Brain) Software Library tool MELODIC (Beckmann and Smith, 2004) was used to analyze the rs-fMRI and extract ICs as detailed in a previous study (Boerwinkle et al., 2019). Pre-processing included deletion of the first five volumes to remove T1 saturation effects, passing through a high-pass filter at 100 s, slice time correction, spatial smoothing of 1-mm full-width at half maximum, and motion corrected by MCFLIRT (Jenkinson et al., 2002), with non-brain structures removed.

Linear registration was performed between the individual functional scans and patients' high-resolution anatomical scans (Jenkinson and Smith, 2001), which was further optimized using boundary-based registration (Greve and Fischl, 2009). Individual rs-fMRI data sets then underwent independent component analysis (ICA) as previously reported (Boerwinkle et al., 2017).

### Expert rs-fMRI evaluation methodology

The SOZ was evaluated by the expert epilepsy surgery conference team and deemed to be consistent with the other acquired data (video EEG and anatomical MRI) with high enough evidence to surgically target the SOZ. Further, the confirmation that the SOZ was deemed true by the treatment team was evidenced by the Engel I and II scores 1 year post-operatively.

The ICA results were viewed by two blinded reviewers (one neurologist and one neurosurgeon) who sorted the ICs into three categories—noise, resting-state network, and rs-fMRI SOZ—by the criteria below. In case of disagreement between the first two reviewers, the opinion of a third reviewer (a neurologist) was used to make the final determination. In this study, there was no disagreement between the blinded reviewers for the selected subjects.

Rs-fMRI was categorized into noise, resting state network (RSN), and SOZ using the following criteria.

#### Noise category

Consistent noise markers in rs-fMRI are reported in the literature (Hunyadi et al., 2015; Boerwinkle et al., 2019). The noise markers reported in different manuscripts are summarized in Table 4.

**Table 4:** Noise markers in fMRI IC.

**Noise independent component (IC) characteristics**
1. A large number of small voxel clusters
2. Cluster peaks in the white matter
3. High overlap with the white matter, the cerebrospinal fluid, or the blood vessels
4. Crescent shape aligning with the brain boundary
5. Sudden changes in the oscillation pattern in the BOLD signal
6. Located within area of signal loss

#### RSN category

These are activations in the MRI images that are spatially located in established anatomical regions. Such regions are highlighted in literature (Boerwinkle et al., 2019) and include “primary sensory motor networks located in the bilateral face area, the bilateral leg area, and the unilateral right- and left-hand regions; language networks primarily located within the left and right inferior frontal gyrus, posterior–superior temporal gyrus, posterior–superior temporal sulcus, posterior–middle temporal gyrus, and the supramarginal gyrus; parietal networks primarily located within the bilateral homologous parietal gyri; frontal networks primarily located within the bilateral premotor, and homologous bilateral frontal gyri; temporal networks primarily located within the bilateral homologous anterior and posterior temporal regions; visual networks located within the bilateral homologous primary and secondary visual association cortices; the default mode network located primarily within the bilateral posterior cingulate gyrus, precuneus, inferior parietal lobules, and medial prefrontal cortex; and the deep gray networks located with the bilateral putamen and bilateral mesial thalami.”

#### SOZ category

SOZ characteristics consist of two types of features: (a) spatial features and (b) temporal features.

##### Spatial features

The activation must be located within the gray matter while not overlapping with the RSN spatial patterns. It must have a bullseye pattern, where two or more overlapping abnormal neuronal IC can be identified, may have an alternating activation and deactivation pattern that does not overlap noise zones, (noise IC characteristics 2, 3, and 4 in Table 4), may extend to ventricles through white matter, and may have irregular borders.

##### Temporal features

The SOZ BOLD signal power spectra must contain dominant frequencies >0.073 Hz, the rs-fMRI SOZ must have power spectra at higher frequencies than RSN, and the BOLD time series may have irregular patterns.

The rs-fMRI IC were sorted by an expert and reported to the clinical epilepsy surgery evaluation team. The data includes ICs extracted using the MELODIC module in FSL (Beckmann and Smith, 2004). Table 3 provides the age and sex distribution and surgical outcome statistics.

### Ethics statement

Institutional IRB for retrospective analysis for this project was approved by the PCH Institutional Review Board (20–358), who determined that, since the retrospective rs-fMRI for these subjects was collected as part of a standard-preoperative MRI, no additional consent procedures were required.

### Data/code availability statement

The data were deidentified according to the National Institutes of Health (NIH) Privacy Rule permits and made available for research application. Further, in accordance with the open science policy, we will provide interested researchers access to EPIK to enable them to reproduce our results.

### EPIK method

**Figure 4:**
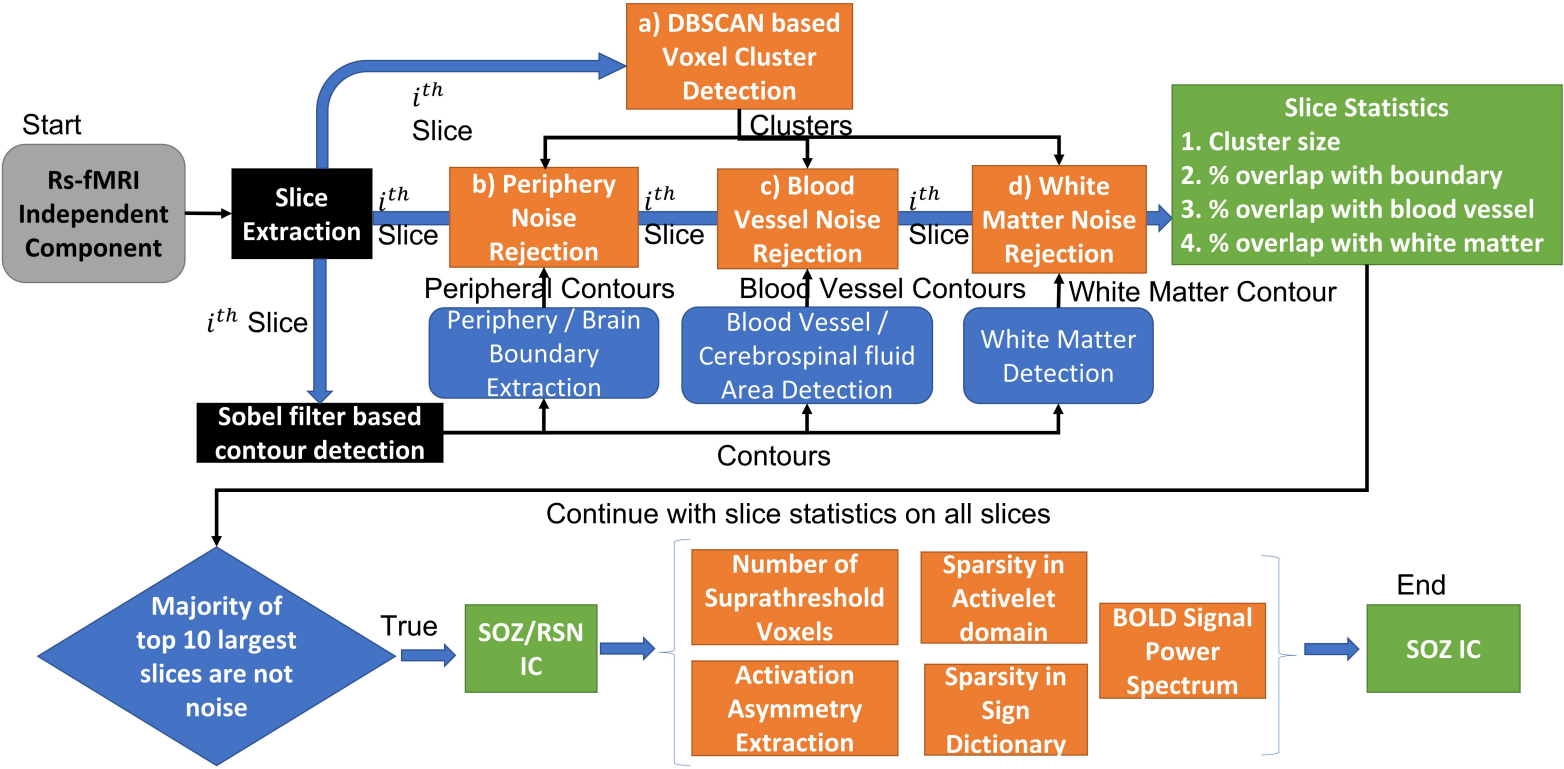
EPIK: Unsupervised approach for SOZ classification. The EPIK method is applied on individual IC to classify it as SOZ or RSN or noise.

**Figure 5:**
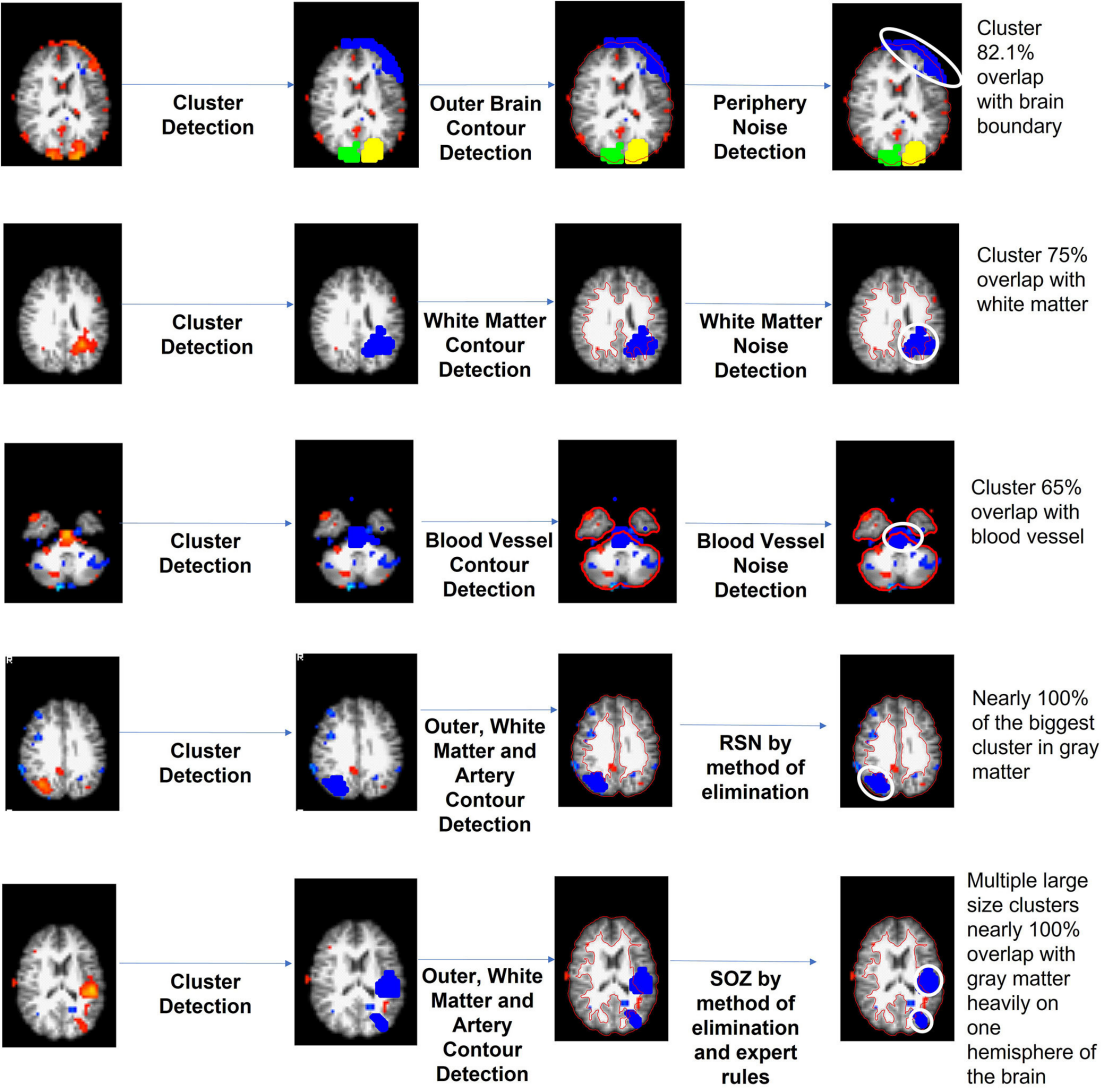
Demonstration of unsupervised IC sorting mechanism.

EPIK (Figure 4) considers noise markers for ICs in an rs-fMRI, as documented in several studies including Griffanti et al. (2014, 2017). The method applies rules in a waterfall technique to classify an IC as noise (Figure 4). If an IC is not noisy, then it classifies the IC as either an RSN or a SOZ. In detail, there are six expert-derived rules for IC noise markers, combined from Boerwinkle and Hunyadi's works (Table 4). Automated application of such rules necessitates the development of the following key components:

a) Voxel cluster detection algorithm: A density-based scanning approach is undertaken to derive voxel clusters (upper panel Figure 4). The algorithm takes two configurable inputs: neighborhood, which includes a distance metric and a value ϵ, and the minimum number of nearby voxels ***v***_**min**_. If a voxel has more than ***v***_**min**_ voxels in the ϵ neighborhood, then it is marked as a core point of a cluster. If a voxel is not a core point but is in ϵ neighborhood of a core point, then it is identified as a border point. All other points are ignored from clusters. Core points, that are in ϵ neighborhood of each other, are combined into one cluster, and border points are assigned to the cluster of the nearest core point. The output of this step is the set of clusters in each IC slice.
b) Brain boundary/periphery detection: Contours in the brain are derived using a Sobel filter-based edge detection technique (Figure 4; Chakraborty et al., 2020). The lowest intensity contour is most likely the outer contour of the brain. However, the cerebrospinal fluid and blood vessels also present as low-intensity contours. The method searches for the contour that encompasses all other contours, which gives us the brain periphery.
c) White matter detection: The white matter manifests as the brightest contour in the brain. The blood vessels and cerebrospinal fluid in the white matter contour are discarded.
d) Blood vessel detection: The major basal-region blood vessels present themselves as low-intensity contours encompassed in the brain periphery contour.
e) Noise IC classification: Utilizing the a, b, c, and d steps, an IC can be classified as noise (Figure 5). From each slice of an IC, the clusters and the contours are extracted. An overlapping cluster can cause the contour detection algorithm to fail in extracting the peripheral, the white matter, and the blood vessel contours. In the initial pass through the ICs, EPIK obtains a version of each slice devoid of clusters, which is subsequently used to identify contours. The algorithm then reruns through each slice of an IC and performs cluster detection. It then evaluates the overlap of the largest cluster with the brain boundary (first row in Figure 5) and the intersection of the largest cluster with the white matter and blood vessels (third row in Figure 5). The output of the first stage classifier (upper panel in Figure 4) is a statistic for each slice on the cluster size, the percentage (%) overlap with the brain boundary, the blood vessels, and the white matter for each cluster in a slice.

Each IC has multiple slices (around 55 for PCH dataset). The second stage classifier sorts the slices in decreasing order of cluster size (lower panel of Figure 2). It selects the top 10 slices and checks the percentage overlap to determine noise slices. If the majority of the top 10 slices are noise, the IC is classified as a noise IC. If the IC passes through the majority evaluation, it is passed to the second-level classifier, which determines if it is a normal RSN or an SOZ (Figure 4). The SOZ classification is based on expert guidance on the SOZ markers in ICs, as documented in Hunyadi et al. (2013; 2015; Table 4).

f) BOLD signal feature extraction: The BOLD signal was first divided into windows of length 256 samples. Four levels of activelet transformation coefficients using “à* trous*” algorithm with exponential-spline wavelets were extracted from each window. Sparsity in the activelet coefficients was evaluated using the Gini Index metric (Lerman and Yitzhaki, 1984). A Gini Index of >0.75 is sparse. If an IC is classified as white matter noise, then it can be classified as an SOZ if the Gini Index in the BOLD signal is >0.75. In addition, sparsity in matching pursuit using a sine dictionary limited to frequencies between 0.01 and 0.1 Hz was also evaluated using the Gini index. If an IC was classified as white noise, then it can be classified as SOZ if the BOLD signal Gini Index in the sine dictionary matching pursuit is >1.72.

### DL strategy for SOZ localization

Nozais et al. (2021) recently proposed a DL-based technique where a multi-layer perceptron (MLP) is trained on 12,690 RSNs from 282 participants. As such, it does not incorporate any expert knowledge but instead attempts to build its own hypothesis from examples. The technique has not been used to classify SOZ and can currently only identify RSNs. We implemented CNN-based DL for SOZ localization.

For the CNN technique, hyperparameter tuning is one of the most important steps. A KerasTuner was implemented to get the optimal values of the hyperparameters. We used a hyperband algorithm with the objective of least validation loss to select the best model of CNN by optimizing the following hyperparameters:

- Number of layers: [3; 4; 5]
- Number of units/filters per layer: min_value = 32, max_value = 512, default = 128.
- Learning rate: [10^−2^; 10^−3^; 10^−4^]
- Dropout rate: [0; 0.2; 0.33; 0.4; 0.5; 0.66].

We used 4,212 ICs for training and 1,404 ICs for validation in the hyperparameter tuning process. The input shape of the IC image was downsampled from 1,006 × 709 × 3 to 270 × 400 × 3 during preprocessing. Binary cross-entropy was used as a loss function, and Adam was used as an optimizer. To avoid the overfitting problem, “dropout” and “early stopping” strategies were implemented. “ReLU” (rectified linear unit), being more computationally efficient, was used as an activation function for the input and hidden layers, and the “Sigmoid” activation function was used for the output layer. For CNN, weights were initialized using the “He uniform” initializer.

### Shallow learning strategy

The technique proposed by Hunyadi et al. (2013, 2014, 2015) was replicated. The rs-fMRI image and BOLD signal features were extracted from the IC images. From the entire pool of ICs, 60% of the data were randomly sampled to be used as training data. The remaining 40% were used for testing. The features extracted from the rs-fMRI image and BOLD signal were used to train a Least Squares Support Vector Machine (LS-SVM), as described by Hunyadi et al. (2013, 2014, 2015).

The following features were extracted from each IC following the study of Griffanti et al. (2014): (a) number of clusters greater than a pixel size of 135; (b) asymmetry of an IC using the difference in the *z*-scored value of the voxels in the left hemisphere and their contralateral voxels in the right hemisphere; (c) sparsity in activelet basis using the Gini index metric; (d) sparsity in sine basis using the Gini index metric. The SVM was then trained for the two-class classification task [(either RSN vs. noise) or (SOZ vs. non-SOZ)]. We utilized two kernels: radial basis function (RBF) and linear kernel. The performance for the linear and RBF kernels was similar and, hence, followed Occam's Razor theory; in this manuscript, we only report the performance for the linear kernel.

### Metrics and statistical analysis method

We evaluated the performance of each approach for two objectives: (a) noise IC removal and (b) SOZ localizing IC identification. For the first objective, we defined true positives (TP) as ICs that are classified as RSN or SOZ by both expert and the automated approach, true negatives (TN) as ICs that are classified as noise by both the expert and the automated approach, false positives (FP) as ICs classified as noise by the expert but RSN or SOZ by the automated approach, and false negatives (FN) as ICs classified as RSN or SOZ by the expert but noise by the automated approach. For the second objective, we define TP as ICs classified by both the expert and the automated approach as SOZ or RSN, TN as ICs classified by both the expert and the automated approach as not SOZ, FP as ICs classified as non-SOZ by the expert but SOZ by the automated approach, and FN as ICs classified as SOZ by the expert but non-SOZ by the automated approach. From these, we derived accuracy, precision, sensitivity, and specificity.

We evaluated the statistical significance of a difference in performance metrics between the two approaches by utilizing a one-sided paired *t*-test. The alternate hypothesis was that there is a positive non-zero difference between EPIK and any other approach (LS-SVM or CNN). The alternate hypothesis was rejected if the *p*-value for the paired *t*-test was <0.05.

We also evaluated the effect of age and gender on each approach using a mixed-effects model with each parameter as the observation variable and age or gender as the predictor variable. A random effect on the patient ID was also introduced. For each algorithm, a separate mixed-effects model was generated for each metric and for each predictor variable, i.e., age/gender.

## Results

### Overall identification results

We compared the performance of EPIK with two competing ML-based approaches: shallow learning (LS-SVM) and deep learning (CNN). For the ML-based approach, training data was used from every subject. This is also known as the user-dependent (Bhakta et al., 2020) supervised classification approach and gave us the best performance metrics. For EPIK, no such training set is needed. The results in Table 5 show that EPIK outperforms both LS-SVM and CNN approaches for SOZ localizing IC identification tasks. The CNN approach is more accurate in noise removal but performs poorly in the SOZ identification task.

**Table 5:** Overall RSN or SOZ identification results for the three approaches.

**Approach**	**RSN or SOZ vs. noise**				**SOZ vs. non-SOZ (RSN or noise)**				**Key observations**
	**Accuracy**	**Precision**	**Sensitivity**	**Specificity**	**Accuracy**	**Precision**	**Sensitivity**	**Specificity**	
EPIK (this paper)	71.7%	73.1%	72%	73.7%	84.7%	74.1%	88.6%	81.9%	Best performance for SOZ identification
LS-SVM (Hunyadi et al., 2014)	61.8%	52.2%	43%	73.6%	80.7%	52.2%	72.1%	78.7%	High false positives and false negatives Significant variance across patients
One sided *t*-test for +ve difference between EPIK and LS-SVM	*p*-value = ~0 [5, 15.2]	*p*-value = ~0 [18.7, 27.4]	*p*-value = ~0 [27.4, 45.9]	Rejected *p*-value = 0.9	*p*-value = ~0 [2, 6.5]	*p*-value = ~0 [20.7, 29.1]	*p*-value = ~0 [14.1 25.3]	Rejected *p*-value = 0.06	
CNN (Nozais et al., 2021)	82.45%	82.7%	82.1%	81.5%	73.5%	28.5%	97.7%	42.85%	Best RSN identification performance. Poor SOZ performance due to lack of hand sorted SOZ IC examples.
One sided *t*-test for +ve difference between EPIK and CNN	Negative change *P*-value ~ 0 [−5.1, −13.2]	Rejected *P*-value = 0.6	Negative change *P*-value ~0 [−7, −12.1]	Negative change *P*-value = 0.02 [−4.1, −9]	*P*-value ~0 [8.3, 15.7]	*P*-value ~ 0 [51.2, 60]	Negative change *P*-value ~ 0 [−4.2, −11.3]	*P*-value = 0.001 [31.6, 45.2]	

EPIK has high sensitivity in the SOZ identification task with a low number of FNs. This implies that EPIK rarely misses any SOZ localizing IC. The LS-SVM approach is poor in noise removal, but its performance improves for the SOZ identification task. The confidence interval is specified as [a,b] for metrics with a *p*-value < 0.5.

### Performance variation with age and gender

Table 6 shows the variation of the performance metrics for EPIK, LS-SVM, and CNN with respect to age and gender. The accuracy, precision, and sensitivity of EPIK for noise removal do not have a statistically stable dependence on age or gender. The specificity of EPIK for noise removal decreases with age, resulting in more FPs, where noise is categorized as RSN or SOZ. For the SOZ identification task, there is a statistically significant trend for sensitivity to increase and specificity to decrease with age. This implies that, as age progresses, EPIK tends to classify more RSN or noise as SOZs; however, fewer SOZs are ignored as noise. Consequently, EPIK is observed to have an accuracy >85% at ages below 5, which is higher than those previously reported.

**Table 6:** Age- and sex-segregated metrics for the unsupervised IC classification algorithm, the LS-SVM approach by Hunyadi et al., and the CNN deep learning approach.

**Metric**	**Algorithm**	**0 < Age ≤ 5 (*N* = 20)**	**5 < Age ≤13 (*N* = 18)**	**13 < Age ≤18 (*N* = 14)**	***P*-value fixed effects on age**	**Men (*N* = 23)**	**Women (*N* = 29)**	***P*-value fixed effects on sex**	**Key observations**
**Noise vs. network/SOZ performance metrics**									
Accuracy	EPIK	**69.4% (±9%)**	**74% (±8.2%)**	**71.7% (±5.8%)**	**0.32**	**73.8% (±5.3%)**	**70% (±9** **.** **4** **%** **)**	**0.04** ^†^	CNN gives the best RSN identification accuracy for all age categories. followed closely by EPIK
	LS-SVM	55.8% (±11.5%)	63.7% (±7.7%)	65.3% (±8.4%)	0.004^†^	63.6% (±9.5%)	59.1% (±10.6%)	0.06	LS-SVM is poorest in identifying RSN since it only considers SOZ markers in ICs.
	CNN	73.2% (±4.5%)	76.1% (±0.6%)	80.2% (±5.8%)	~0	72.8% (±8.2%)	77.4% (±4.7%)	0.09	Success of CNN can be attributed to availability of a significant number of normal RSN ICs (*n* = 2,427)
Precision	EPIK	**74.9% (±16.2%)**	**73.6% (±13.7%)**	**66.5% (±10%)**	**0.048**	**73.5% (±11.5%)**	**71.1% (±16%)**	**0.27**	
	LS-SVM	55.6% (±32.4%)	52.8% (±15.9%)	46.5% (±18%)	0.3	54.8% (±22.2%)	50.1% (±25.4%)	0.24	
	CNN	68.2% (±11.7%)	75.2% (±1.5%)	75.4% (±7.3%)	~0	69.2% (±13%)	75.91% (±15.1%)	0.3	
Sensitivity	EPIK	**63% (±18%)**	**76.6% (±9.3%)**	**76.8% (±9.7%)**	**0.001** ^†^	**75.2% (±10.7%)**	**68.43% (±16.9%)**	**0.047**	
	LS-SVM	27.5% (±25.9%)	50.8% (±26.9%)	55.1% (±22%)	0.001^†^	52.6% (±28.9%)	35.4% (±24.9%)	0.012^†^	
	CNN	86.09%	81.5%	85.96%		78%	79.5%		
Specificity	EPIK	**79% (±13.8%)**	**72.7% (±17.1%)**	**68.2% (±8.4%)**	**0.01** ^†^	**73.4% (±13.5%)**	**74.2% (±15.2%)**	**0.41**	
	LS-SVM	80.5% (±17.4%)	68.8% (±23%)	70% (±10%)	0.035^†^	71.3% (±17%)	75.5% (±19.9%)	0.2	
	CNN	60.5%	70.8%	75%		67.9%	75.31%		
**SOZ identification metrics**									
Accuracy	EPIK	**87.5% (±7.6%)**	**83.5% (±9.6%)**	**82.2% (±6.1%)**	**0.025** ^†^	**84.6% (±6.7%)**	**84.7% (±9.3%)**	**0.48**	EPIK has the best performance for SOZ localizing IC identification
	LS-SVM	85.3% (±6.6%)	77.2% (±9.4%)	78.6% (5.7%)	0.008^†^	79.5% (±8.8%)	81.6% (±7.7%)	0.17	EPIK has consistent performance across age.
	CNN	75.5% (±27.7%)	75.3% (±26.6%)	76.5% (±21%)	0.8	71% (±28.2%)	73% (±30.2%)	0.44	EPIC has the best performance for children of age <5 years. This is a key benefit because it is known that earlier surgery for epilepsy yields better surgical and developmental outcomes.
Precision	EPIK	**76.7% (±16.3%)**	**75.2% (±14.4%)**	**69.2% (±9.9%)**	**0.07**	**76.3% (±10.7%)**	**72.5% (±16.5%)**	**0.17**	
	LS-SVM	62.5% (±17.2%)	56.9% (±15%)	51.4% (±15%)	0.06	54.7% (±15.7%)	55% (±16.3%)	0.2	
	CNN	53.8% (±50.2%)	50% (±51.4%)	45% (±50%)	0.035	45.9% (±49.9%)	54.4% (±50%)	0.54	
Sensitivity	EPIK	**86.8% (±8** **.** **8%)**	**89.4% (±6.8%)**	**90.4% (±7.6%)**	**0.085** ^†^	88.1% (±7.1%)	89.1% (±8.5%)	0.34	
	LS-SVM	58.8% (±33.9%)	74.6% (±19.5%)	87.8% (±23%)	0.001^†^	78.83% (±25.6%)	66.7% (±30.4%)	0.065	
	CNN	11.1% (±3%)	0 (±0)	0 (±0)	0.001	20% (±5%)	3.44% (±3%)	0.002	
Specificity	EPIK	**86.6% (±12.8%)**	**79.9 % (±15.6%)**	**77.8% (±8%)**	**0.02** ^†^	81.7% (±12.1%)	82% (±14.2%)	0.47	
	LS-SVM	86.4% (±14.1%)	73.2% (±21.8%)	74.9% (±9.3%)	0.015^†^	75.3% (±17%)	81.5% (±17%)	0.09	
	CNN	74.9% (±27.6%)	75.3% (±26.6%)	76.1% (±22%)	0.4	75.37% (±28.2%)	76.59% (±30%)	0.8	

† Indicates that the result has a p-value of < 0.05 and is statistically significant. Bold value refers to our technique EPIK's results.

The LS-SVM approach had consistently better performance for the SOZ identification task than noise removal. It also had the same pattern of increasing sensitivity and decreasing specificity with age. The LS-SVM approach had a higher variance in performance across subjects. This indicates that the hand-crafted features chosen by Hunyadi et al. may be less applicable to specific scenarios of DRE in children.

The CNN approach outperformed EPIK and LS-SVM for all age groups for noise removal. However, it had a lower performance for SOZ identification. In the training data, there were only 318 SOZ localizing ICs as opposed to 2427 RSN IC. This may have led to an underfitting of the CNN technique for SOZ identification. For the CNN technique in noise removal, both sensitivity and specificity increased with age. This potentially indicates that the CNN technique is finding novel hidden features from the ICs that are characteristic of RSN but not SOZ.

Overall, EPIK provided a consistent performance across the three age categories considered in this study compared to prior reported methods. Whereas, the ML techniques of Hunyadi et al. (2015) and CNN have significantly higher variance, possibly indicating inconsistent performance.

### Performance on subjects undergoing surgery

Out of the 52 subjects considered in this study, 24 underwent surgery. The surgical outcomes were varied with 16 subjects becoming seizure-free (Engel I) after surgically destroying an expert-identified SOZ using rs-fMRI and seven having reduced post-operative seizure frequency (Engel II; Table 7). We focused on EPIK and LS-SVM for the SOZ identification task on the 24 subjects that underwent surgery because CNN had significantly poorer performance than the other two.

**Table 7:** Performance of EPIK and LS-SVM approaches for subjects undergoing surgery.

**Age, years (months)**	**Sex**	**Pre-surgery frequency (per month)**	**Post-surgery frequency (per month)**	**Procedure**	**Method**	**Accuracy**	**Precision**	**Sensitivity**	**Specificity**
18 (0)	W	1	0	A^*^	EPIK	82.8%	71.7%	82.5%	82.9%
					LS-SVM	78.7%	58.8%	76.9%	79.4%
14 (8)	M	1	0	A	EPIK	85.2%	75.5%	90.2%	82.1%
					LS-SVM	68.2%	48%	92.3%	58.1%
14 (7)	W	3	1 (66% reduced)	A	EPIK	86.5%	78.2%	95.6%	79.7%
					LS-SVM	71.4%	52%	100%	58.6%
14 (10)	M	1	0	A	EPIK	86.3%	77.1%	86.1%	86.4%
					LS-SVM	82%	35.7%	100%	80%
16 (3)	W	210	0	A	EPIK	86.5%	67.9%	97.4%	82.4%
					LS-SVM	91.3%	37.5%	100%	63.5%
10 (1)	W	240	0	A	EPIK	74.2%	60.5%	95.8%	60.5%
					LS-SVM	76%	60%	100%	62.5%
8 (2)	M	4	2 (50% reduced)	A	EPIK	83.3%	77%	94.1%	73.1%
					LS-SVM	85.4%	71.4%	83.3%	86.2%
15 (6)	W	12	0	A	EPIK	86%	62.9%	78.6%	88%
					LS-SVM	87.3%	0	0	97.9%
10 (5)	W	60	8 (87% reduced)	A	EPIK	85.5%	69.6%	91.4%	82.9%
					LS-SVM	83%	40%	66.7%	85.4%
3 (2)	W	60	1 (98% reduced)	A	EPIK	87.4%	78%	86.5%	87.8%
					LS-SVM	88.9%	81.8%	75%	94%
17 (9)	M	2	0	A	EPIK	74.3%	50%	88.9%	69.2%
					LS-SVM	75%	25%	66.7%	76%
11 (8)	M	1	0	R^*^	EPIK	80.6%	62.5%	65.8%	85.8%
					LS-SVM	82.8%	16.7%	16.7%	90.4%
4 (9)	W	2	0	R	EPIK	84.1%	72.7%	84.2%	84%
					LS-SVM	80.4%	14.3%	25%	85.7%
18 (1)	W	5	0	R	EPIK	72%	55.6%	87%	64.4%
					LS-SVM	71.4%	50%	75%	70%
10 (5)	W	8	0	R	EPIK	57.5%	44.1%	93.8%	38.7%
					LS-SVM	63.1%	58.8%	100%	22%
13 (8)	M	120	1 (99% reduced)	R	EPIK	82.9%	72.9%	93.5%	75.4%
					LS-SVM	77.8%	43.7%	87.5%	75.7%
2 (7)	W	2	0	D^*^	EPIK	84.5%	53.2%	100%	81.3%
					LS-SVM	81.8%	38.5%	100%	79.5%
2 (7)	M	720	0	A	EPIK	89.1%	81.3%	88.6%	89.3%
					LS-SVM	90.4%	50%	60%	93.6%
0 (3)	W	90	30 (66% reduced)	A	EPIK	98.8%	100%	92.9%	100%
					LS-SVM	97.1%	100%	50%	100%
2 (10)	W	300	0	R	EPIK	84.8%	74.5%	97.4%	75.5%
					LS-SVM	89.2%	71.4%	100%	85.2%
2 (11)	M	4	0	D	EPIK	69.1%	61%	96.2%	44.8%
					LS-SVM	81.8%	78.9%	100%	42.8%
2 (1)	W	3,000	0	A	EPIK	88.4%	45%	60%	91.6%
					LS-SVM	88.1%	25%	20%	94.4%
3 (6)	M	30	0	A	EPIK	88.6%	78.6%	91.7%	87%
					LS-SVM	78.6%	68.4%	100%	60%
1 (4)	F	180	0	R	EPIK	94.1%	94.3%	84.6%	98%
					LS-SVM	88.9%	50%	16.7%	98%
**Agreement with expert hand classification for seizure-free/reduced post-operative outcome**				
EPIK	83.3% (8.43%)	69.45% (13.6%)	88.9% (9.6%)	79% (14.4%)
LS-SVM	80.45% (9.8%)	47.6% (23.9%)	72.5% (32.6%)	75.5% (19.5%)
**Agreement with expert hand classification for ablation procedures**				
EPIK	85.5% (5.8%)	71.6% (13.4%)	88% (9.3%)	82.9% (9.6%)
LS-SVM	82.8% (8.1%)	50.2% (24.9%)	72.7% (30.5%)	79.3% (15.3%)
**Agreement with expert hand classification for resection procedures**				
EPIK	79.4% (11.6%)	68.1% (16%)	86.6% (10.5%)	74.5% (18.9%)
LS-SVM	79.1% (9.4%)	43.6% (21.1%)	60.1% (39.1%)	75.3% (25.2%)

The table also shows the difference in agreement between the automated approach and the expert hand classification for subjects with seizure-free/reduced frequency post-operative outcomes and without any change in seizure frequency.

* A, Ablation; R, Resection; D, Disconnection.

**Figure 6:**
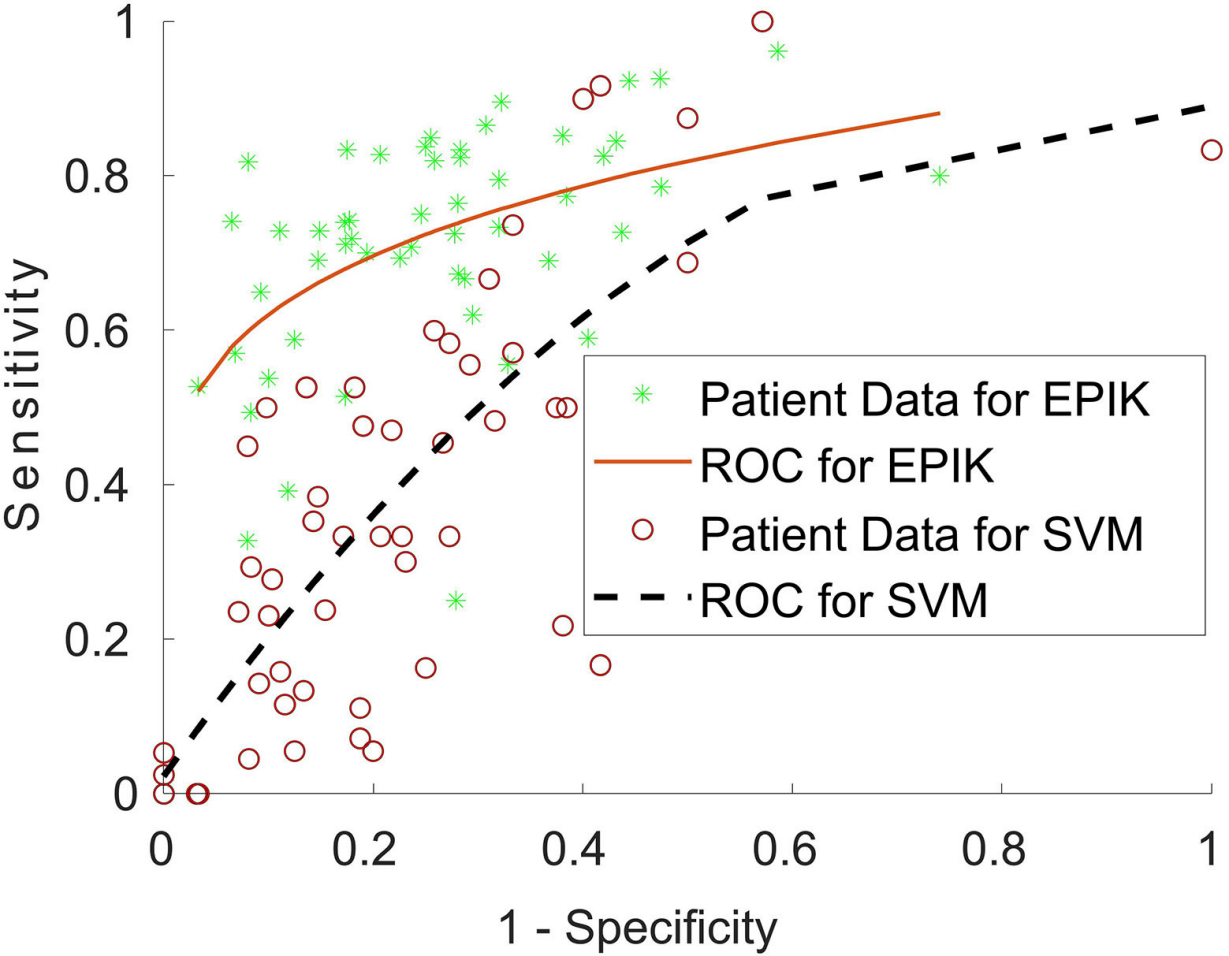
Receiver operating characteristics (ROC) curve for EPIK and the LS-SVM approach for patients undergoing surgery. A curve close to the top left-hand corner of the graph is favorable and shows a balance between sensitivity and specificity.

Table 7 shows that, for subjects whose post-operative outcomes are either seizure-free or have significantly reduced frequency, the agreement between EPIK and expert-hand classification is significantly high (88.9% sensitivity and 79% specificity). Although the LS-SVM approach has nearly similar accuracy as EPIK, the sensitivity is far lower in LS-SVM, with significant individual variance. To better understand the difference between EPIK and the LS-SVM approach, Figure 6 shows the receiver operating characteristics (ROC) curve for both EPIK and LS-SVM. EPIK exhibits higher sensitivity and specificity than LS-SVM, which appears to possibly sacrifice one for the other.

For patients undergoing ablation surgery, the specificity for EPIK was 82.9%, while the sensitivity was 88%. This is preliminarily an encouraging result, given that ablation is minimally invasive and thus largely accepted as less risky than resection. The specificity and sensitivity in EPIK for patients undergoing resection reduce to 79.5 and 86.6%, respectively. Of the 15 subjects who underwent the ablation procedure, 10 were seizure-free (Engel 1 outcome), which is slightly better than recently reported statistics [66% in this study vs. 60.4% reported in Kanner et al. (2022)]. Supplementary Table 1 gives the SOZ location and fMRI evidence of SOZ for all subjects in the study.

### Reduction in IC sorting effort for the neurosurgeon/neurologist

The ICs marked as SOZ by the EPIK method can be supplied to the neurosurgeon or neurologist for localization of SOZs in the brain. The number of SOZ classifications in EPIK per subject is 22 (±4). Out of 22, 16 are true positive SOZ ICs, two are noise ICs, and four are RSN ICs. These ICs are then evaluated by the neurosurgeon or neurologist for determining SOZ in the brain. This implies that there is ~5 times reduction in the number of ICs to be analyzed by the neurosurgeon or neurologist. This can significantly aid in presurgical screening by reducing the cognitive burden of the neurosurgeon or neurologist and improving the accuracy of the SOZ identification.

## Discussion

A strength of EPIK, which may increase its utility, is that it does not require any prior training data and hence it uses a plug-n-play IC sorting method. EPIK combines spatial and temporal markers specific for RSN and SOZ, which results in possibly equivalent or better performance than prior methods. The waterfall technique removes the number of noise ICs using well-established expert rules; hence, it may reduce false positives and increase true positives of SOZ localizing ICs.

For subjects with good postoperative outcomes, there was excellent agreement between expert hand sorting and EPIK-based SOZ localizing IC identification. Also, EPIK appeared to perform well in those <5 years of age, in whom surgery yields improved developmental outcomes (Pindrik et al., 2019; Perry and Shandley, 2021).

The LS-SVM approach did not perform as well for the noise identification task but did show a drastic improvement in performance for the SOZ identification task. This was expected because the hand-selected features proposed by Hunyadi et al. (2013, 2015) are specifically geared toward the SOZ identification task. However, LS-SVM exhibits significant variance in performance across subjects, resulting in inconsistent accuracy in this study. EPIK had a higher and more consistent balance in the identification of all three categories of IC compared to LS-SVM herein.

The CNN approach had a lower performance for SOZ identification. However, there was a significant improvement in the performance of the noise identification task. This can be explained by the difference in data availability for the two tasks. This gives confidence that CNN can perform better if given an adequate number of training-SOZ-localizing ICs; this could be an avenue for future research.

The general assumption in supervised machine learning is that elements from each class come from a unique distribution specific to the class. The ML technique then attempts to learn the differences in the distribution of each class and evaluate the best fit distribution for the test data. The fundamental limitation of the LS-SVM approach is that SVM is inherently a two-class classifier. Although there are multi-class versions of SVM, the multi-class classification is performed in stages, where each stage is a two-class classifier. For rs-fMRI sorting, this would mean that the RSN and noise class will have to be combined into one composite class, while the SOZ ICs are labeled as the class of interest. In rs-fMRI, noise ICs are composed of several different categories of noise such as peripheral noise, white matter noise, and artery noise. Each such noise characteristic has different feature distributions; however, they are considered to be the same class by the supervised ML technique. Moreover, the noise class is combined with the RSN class to make a non-SOZ composite class. Hence, the non-SOZ composite class for rs-fMRI ICs has a composite distribution. As such, it is very difficult for the supervised ML classifier to learn the unique distribution of the non-SOZ IC class. A way around this is to learn each kind of noise and RSN separately. However, that requires data for each kind of noise from each patient. This cannot be guaranteed in a practical real-life setting.

The performance of the CNN-based DL strategy suffered because of the differences in the size of the three classes. RSN and noise classes had a nearly balanced data size, and the CNN strategy had good performance in distinguishing between them. However, since there are very limited SOZ IC examples, the CNN strategy could not reliably identify them.

The unsupervised technique utilizes expert knowledge and image processing algorithms to detect each kind of noise without the need for training a machine. Hence, it learns the noise characteristics without utilizing noise data from each patient. This capability of the unsupervised technique to employ specific algorithms for each type of noise and RSN is one of the major reasons for its success in separating noise, RSN, and SOZ ICs.

### Limitations and future directions

This study (*n* = 52) evaluated a small group of data, and prior automated methods perform well on small samples but have reduced performance on larger datasets; hence, EPIK needs large set validation, which is a future direction. Larger datasets for focused performance evaluation within each age bracket, including young and separately older adults, and the very young vs. middle childhood are needed. Subtypes of epilepsy—acquired, congenital/genetic—and surgical approaches' success metrics should be statistically evaluated with acceptable power. Last, repeat studies in the same individuals over time would increase knowledge of the validity and reproducibility of the tool.

The majority of the subjects in this study received propofol infusion for sedation as part of a standard of clinical care for epilepsy surgery evaluation. Head motion maximum was <1 mm of frame wise displacement in any direction. Although propofol use has minimal effect on the overall rs-fMRI BOLD signal, it puts small but additional risks on the child (Pizoli et al., 2011). Several research studies proposed alternate methods of reducing head motion by engaging the child with videos and post-processing by measuring and accounting for head movements through computational methods (Dosenbach et al., 2017; Greene et al., 2018; D'Andrea et al., 2022). An area of future study is to evaluate the effect of sedation on the EPIK SOZ identification accuracy and integration of live motion monitoring and reduction-based approaches toward the elimination of head movement artifacts.

## Conclusion

1. EPIK identified seizure onset zone (SOZ) localizing resting-state fMRI-independent components in children with drug-resistant epilepsy with an accuracy of 84.7% in this preliminary study.
2. EPIK can reduce the number of potential ICs to be analyzed by the neurosurgeon by ~5-fold, hence significantly reducing the time commitment for pre-surgical evaluation.
3. EPIK is unsupervised and does not need any prior example of SOZ and works by codifying expert knowledge about fMRI noise and SOZ markers.
4. EPIK had consistent performance across age and gender and has been validated with surgical outcomes.
5. EPIK appeared to perform best for those under 5 years of age and thus may enable successful surgeries early in their life, potentially improving long-term postoperative outcomes.
6. EPIK preliminarily performed as well or better than shallow and deep learning systems for the identification of SOZ localizing ICs in a resting-state fMRI.

## Data availability statement

The raw data supporting the conclusions of this article will be made available by the authors, without undue reservation.

## Ethics statement

The studies involving human participants were reviewed and approved by Phoenix Children's Hospital. Written informed consent to participate in this study was provided by the participants' legal guardian/next of kin.

## Author contributions

AB was responsible for designing (with inputs from SKSG), implementing, writing, and statistical analysis of the EPIK methodology. PK was responsible for implementing, writing, and analyzing the DL technique for the automated classification of noise, RSN, and SOZ ICs. SW was responsible for rs-fMRI data's administrative, technical, and material support. BS was responsible for subject identification and data collection. SG was responsible for the revision of this manuscript and validating the authenticity of this study. VB conceived the project, evaluated the stepwise methodological design and intermediate results, responsible for providing the motivation and perspective of this work with respect to epilepsy care, analysis, and interpretation of rs-fMRI data, and providing explanation and guidelines of noise, RSN, and SOZ biomarkers. All authors have read and approved the final manuscript.
