# Supplementary material for: Automated seizure onset zone locator from resting-state functional MRI in drug-resistant epilepsy
Source: Front Neuroimaging. 2023 Jan 4;1:1007668. doi: 10.3389/fnimg.2022.1007668 (PMC10406253; doi:10.3389/fnimg.2022.1007668)
Supplement: Supplementary file 1 [file Table_1.docx]

| Age Years (months) | Sex | Location rs-SOZ; anatomical MRI findings | fMRI evidence | | Anatomical MRI |
| --- | --- | --- | --- | --- | --- |
| 18 (0) | F | L mesial temporal SOZ;  L mesial temporal sclerosis (MTS) | |  | 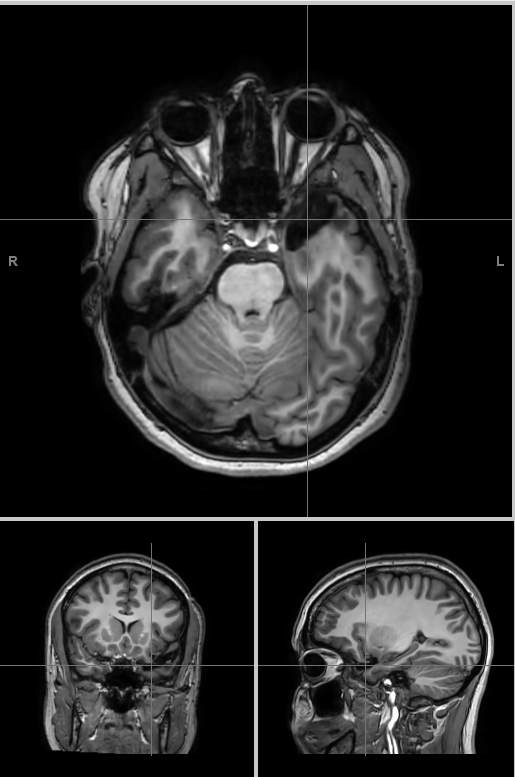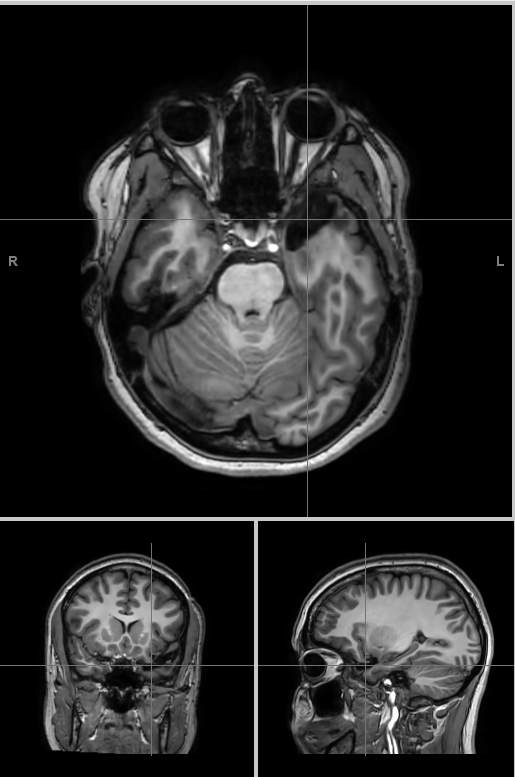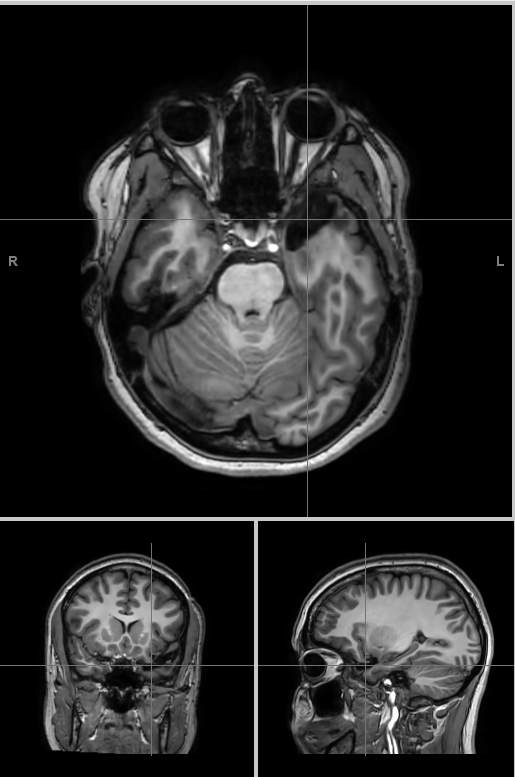 |
| 14 (8) | M | L MT SOZ;  L MTS | |  | 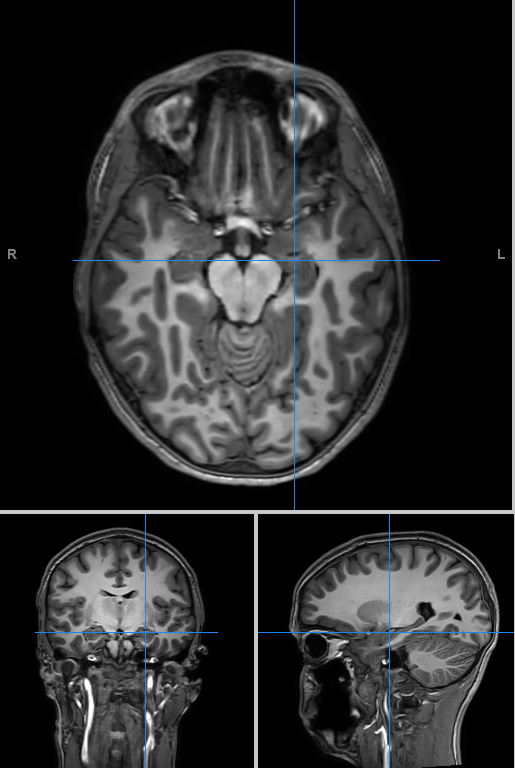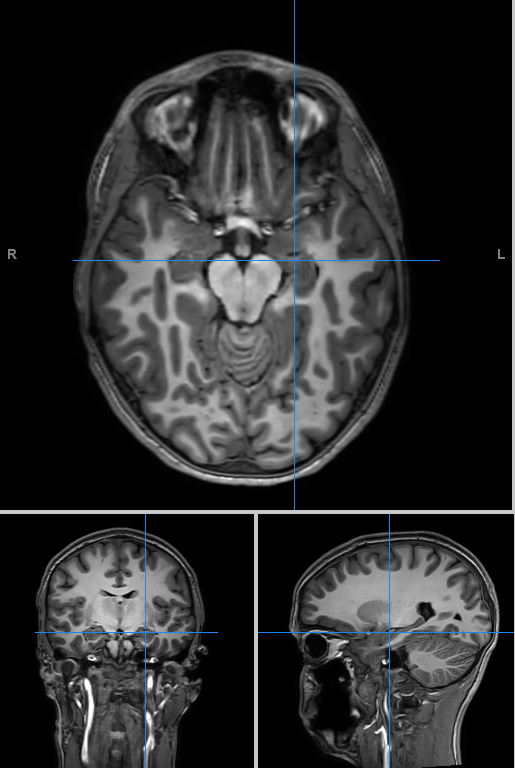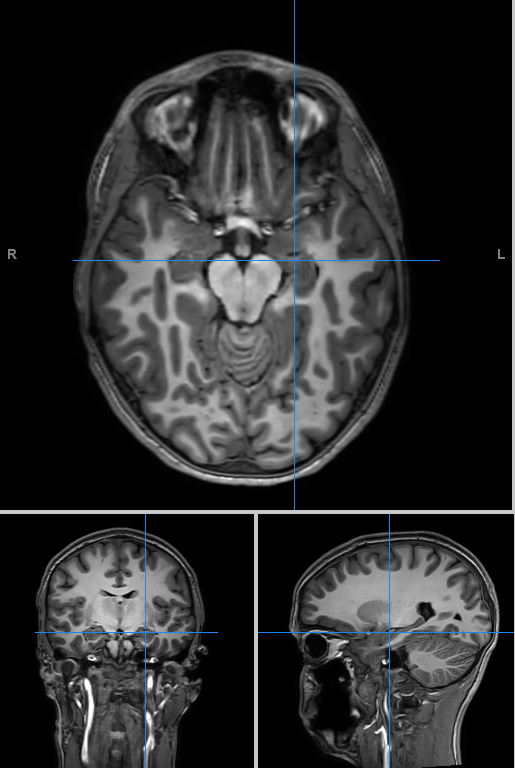 |
| 14 (7) | F | Midline Right (R) frontal (F) SOZ;  R frontal Focal cortical dysplasia (FCD) | | 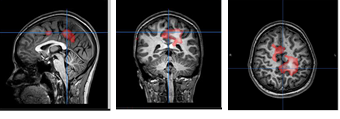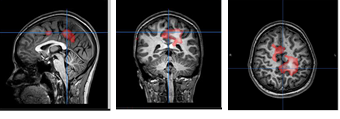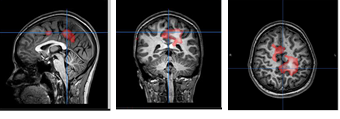 | 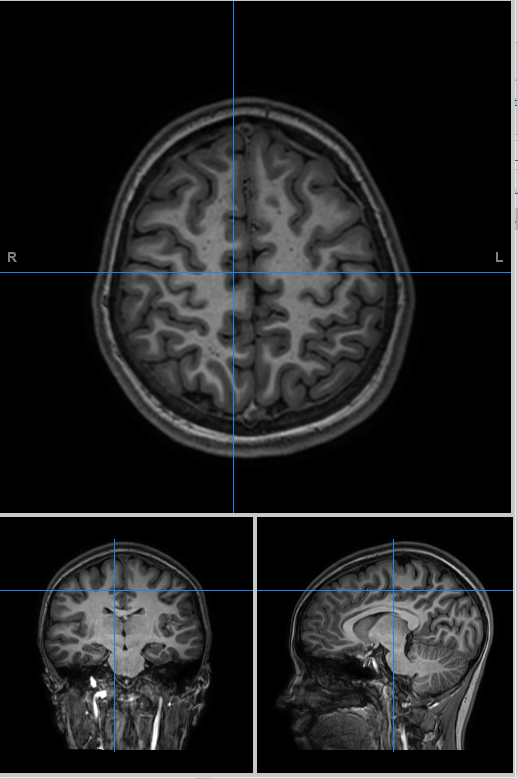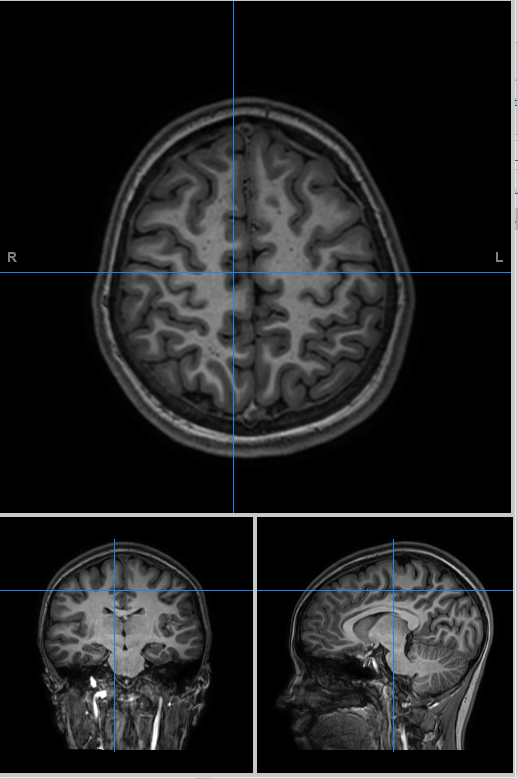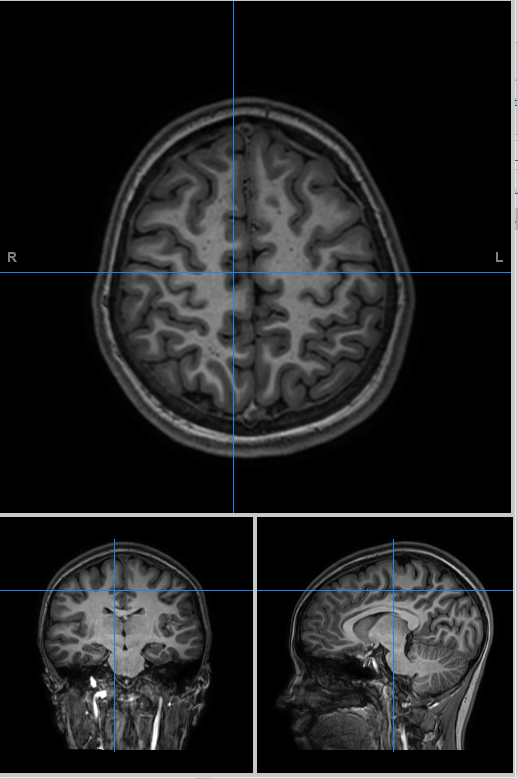 |
| 14 (10) | M | R frontal, parietal  MRI negative | | 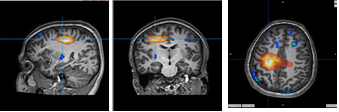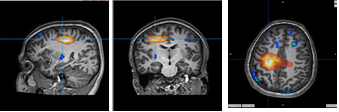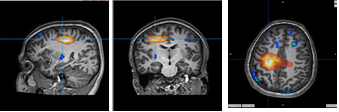 | 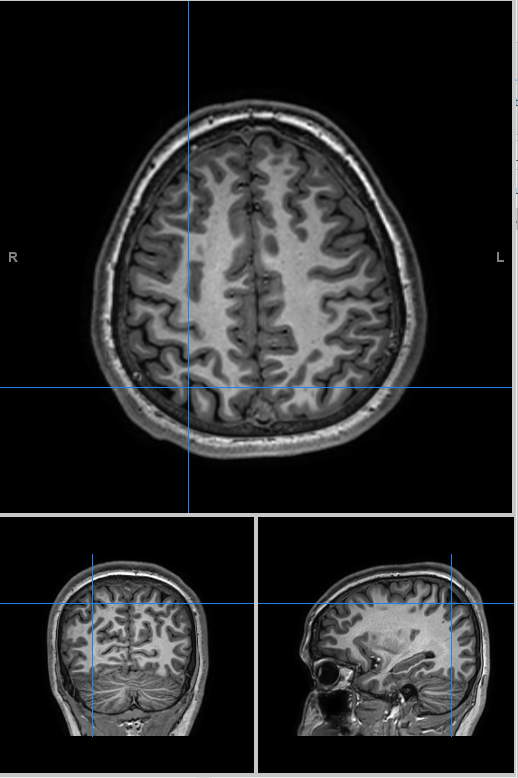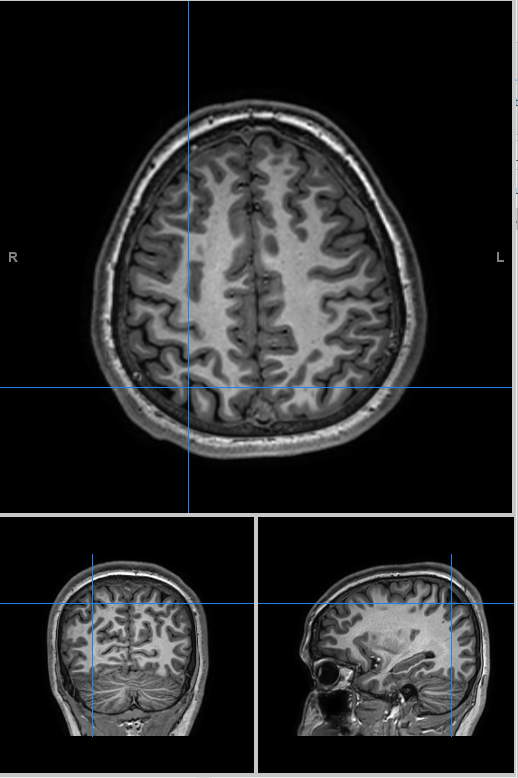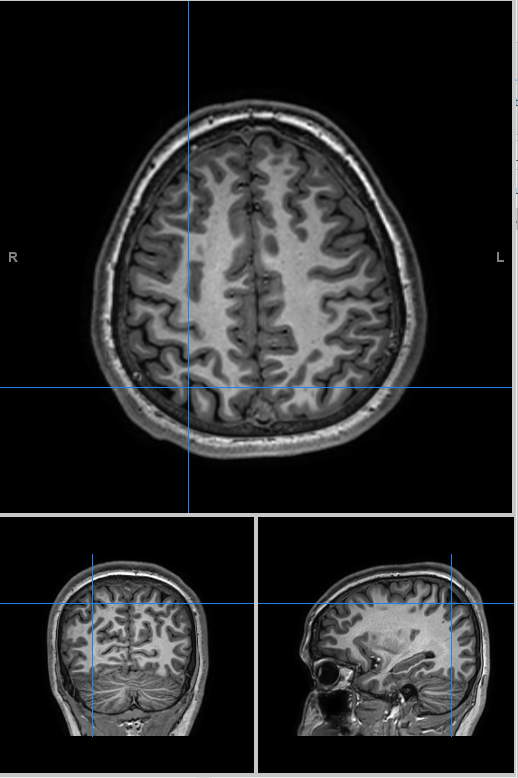 |
| 16 (3) | F | L insular frontal region;  L MT hypointensity | | 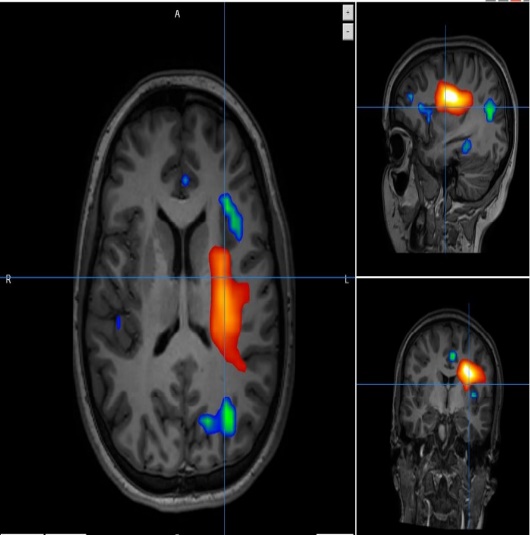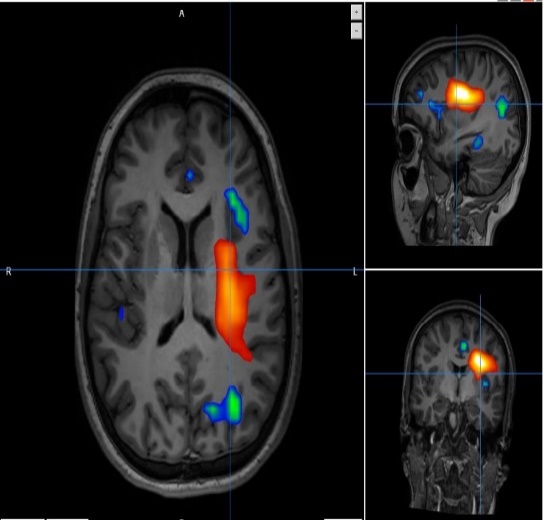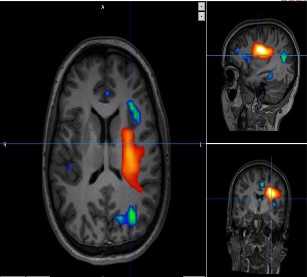 | 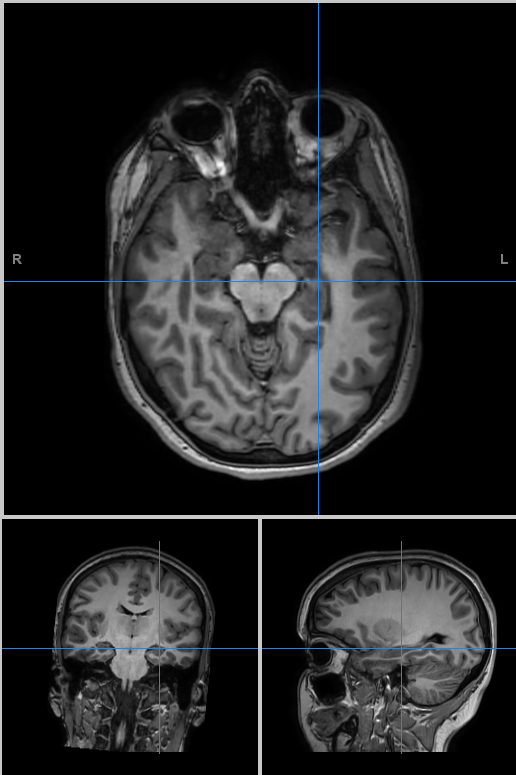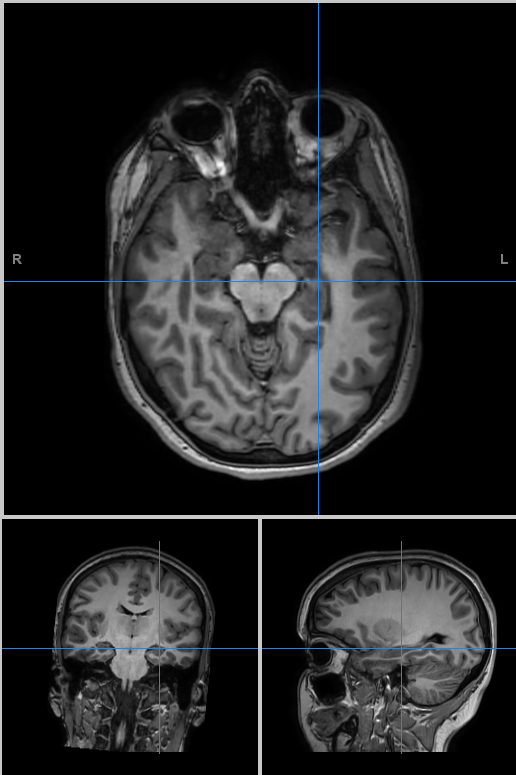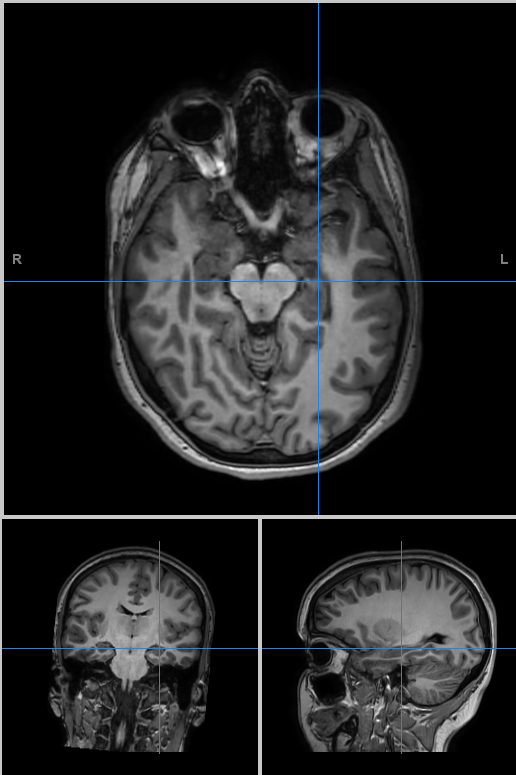 |
| 10 (1) | F | R and L pre-frontal cortex;  non-lesional | | 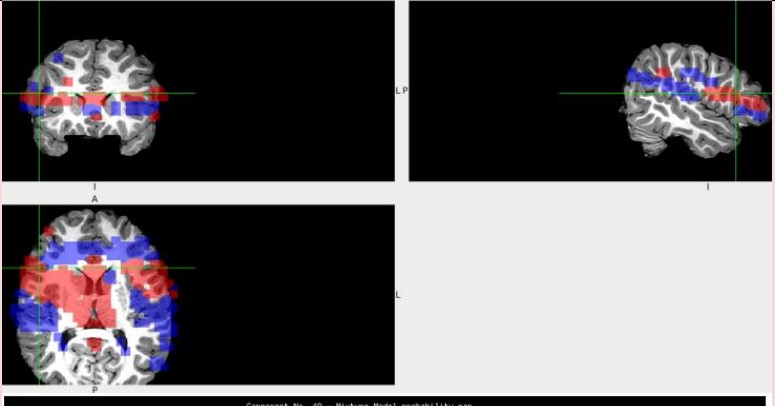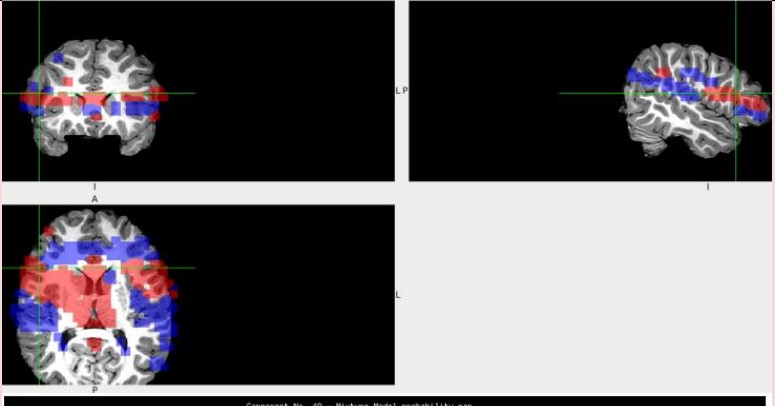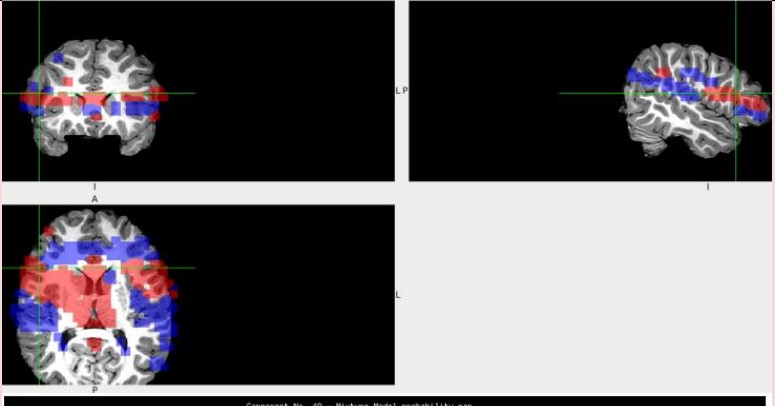 | 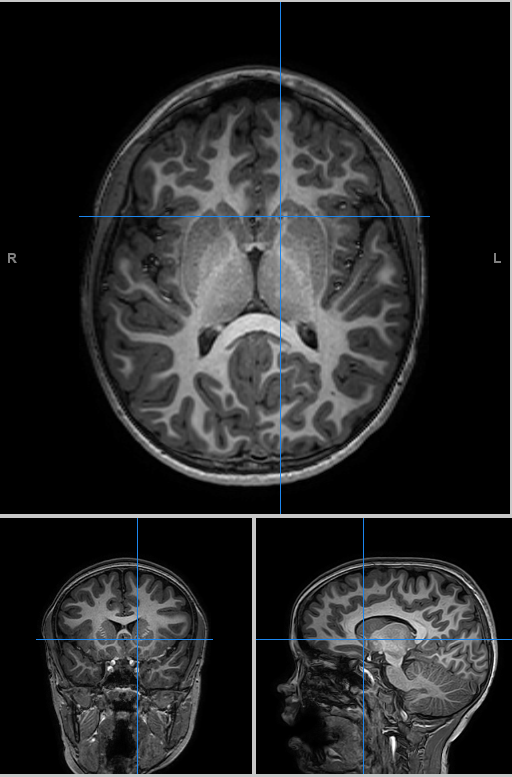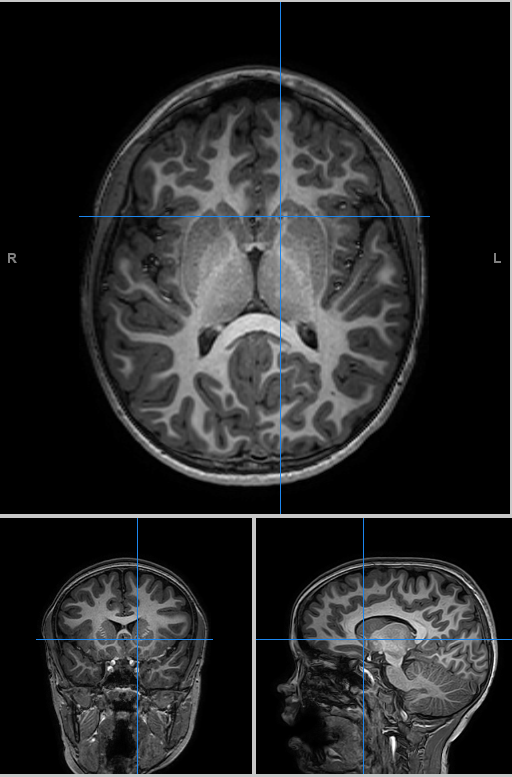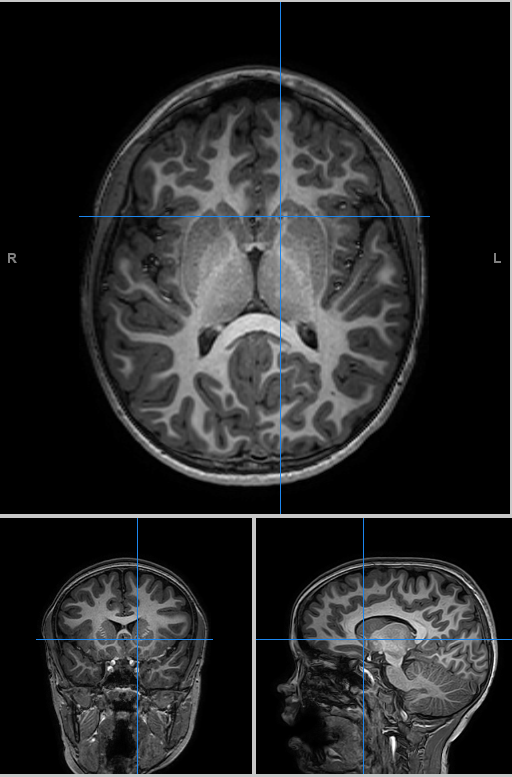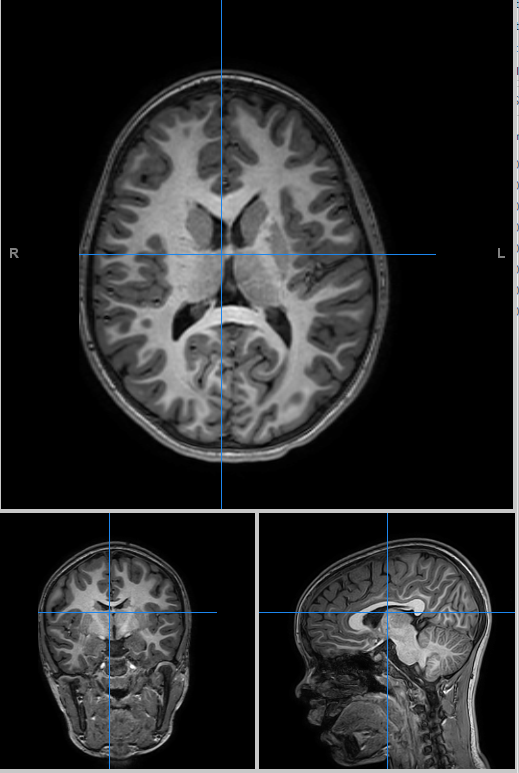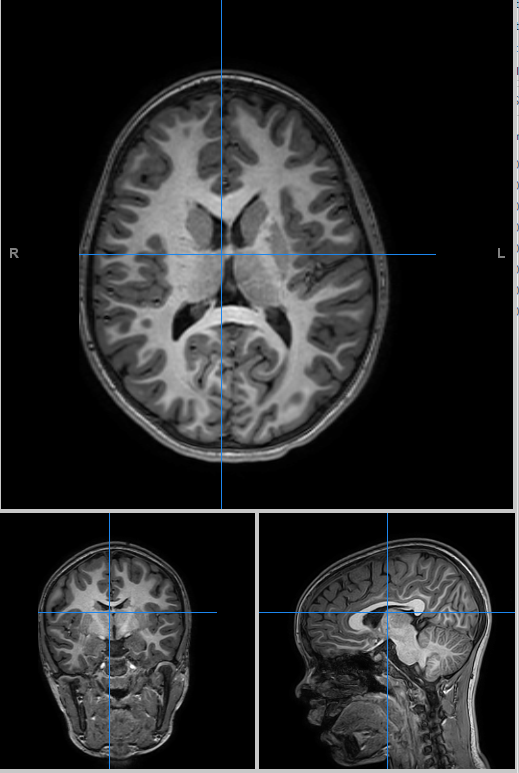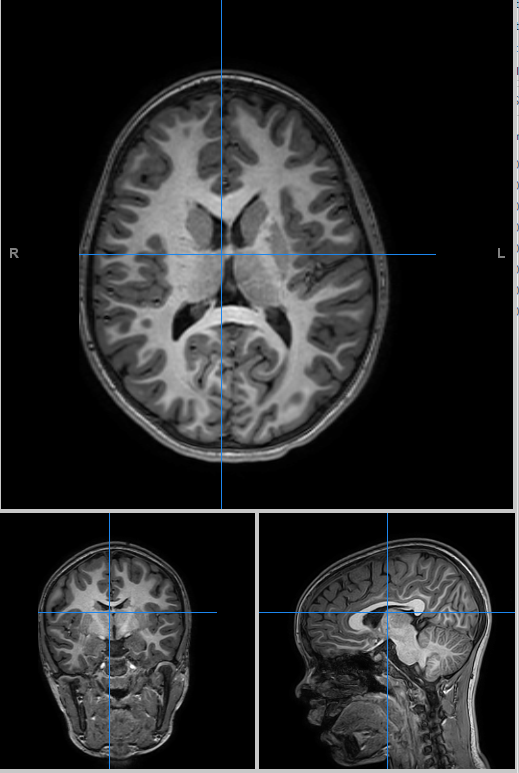 |
| 8 (2) | M | L & R MT- Anterior Temporal (AT) SOZ;  L caudate enlarged perivascular space | | 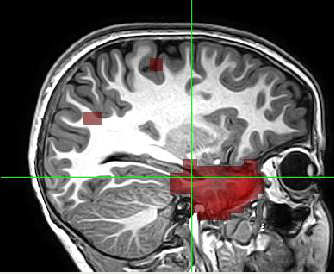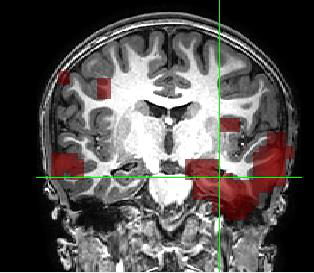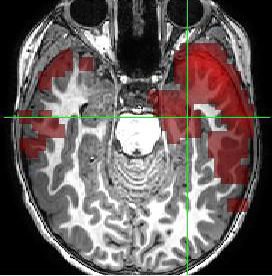 |  |
| 15 (6) | F | L & R MT AT SOZ:  L & R - MTS | |  | 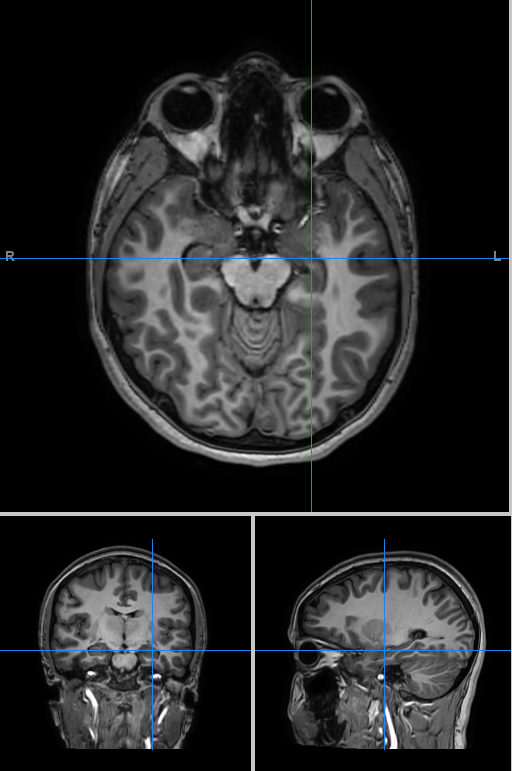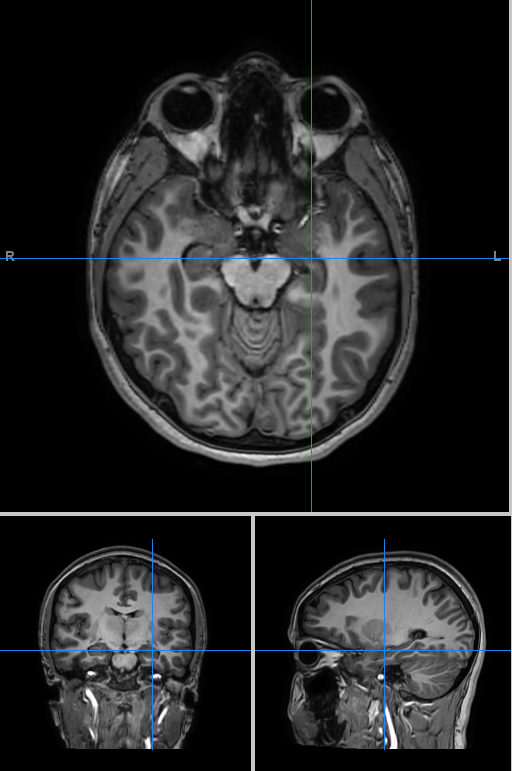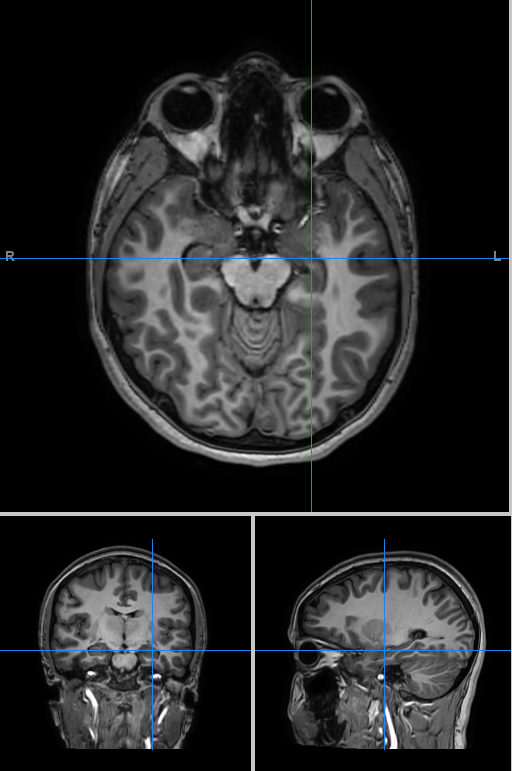 |
| 10 (5) | F | L & R posterior insula region;  MRI negative | | 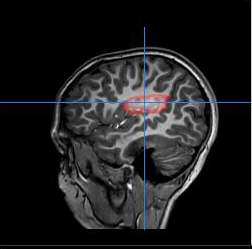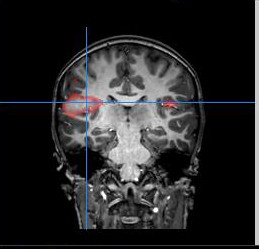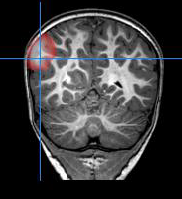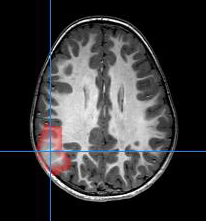 | 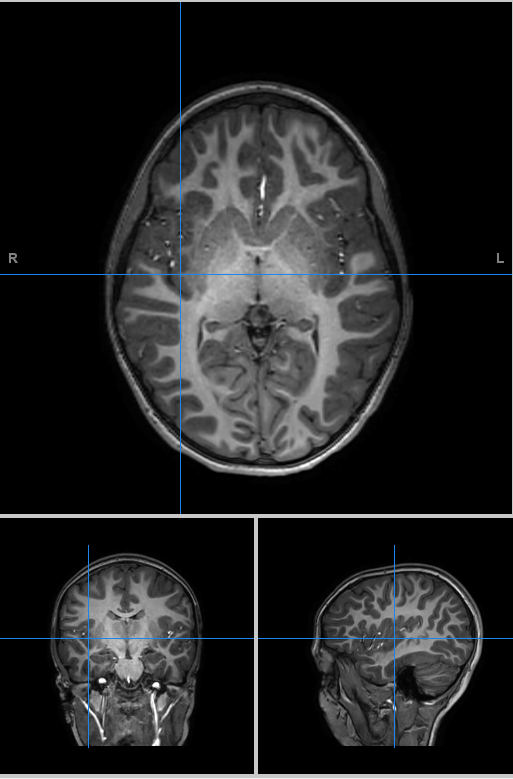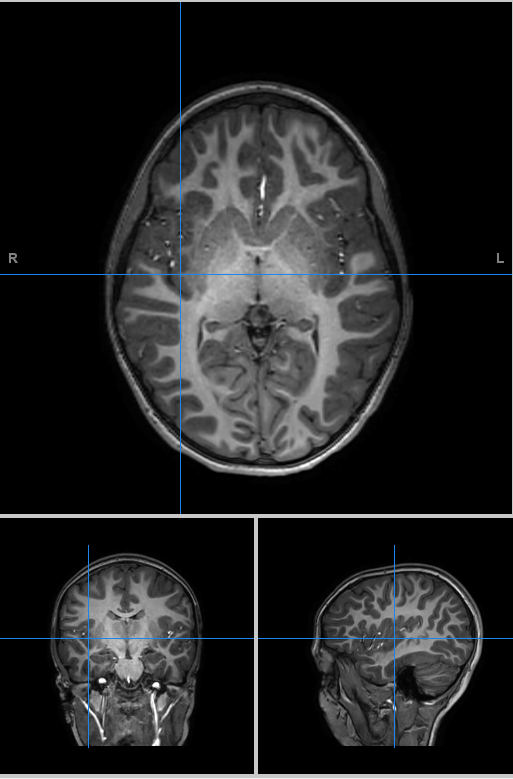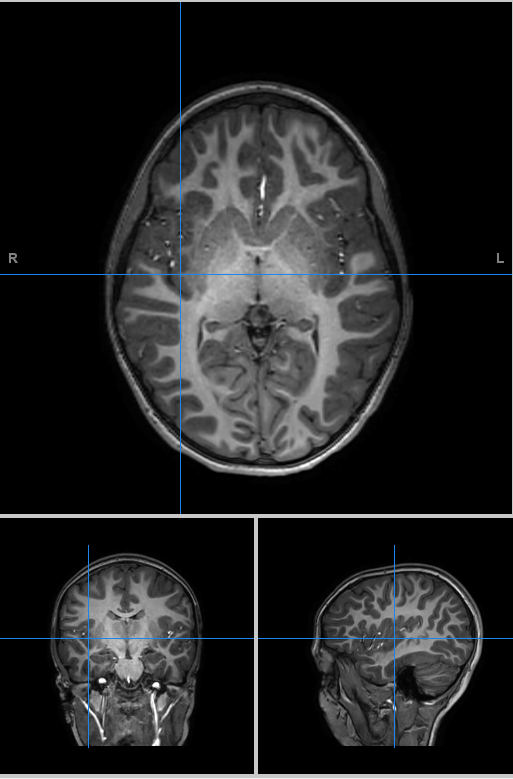 |
| 3 (2) | F | R parietal region, frontal, insula;  R MTS | |  | 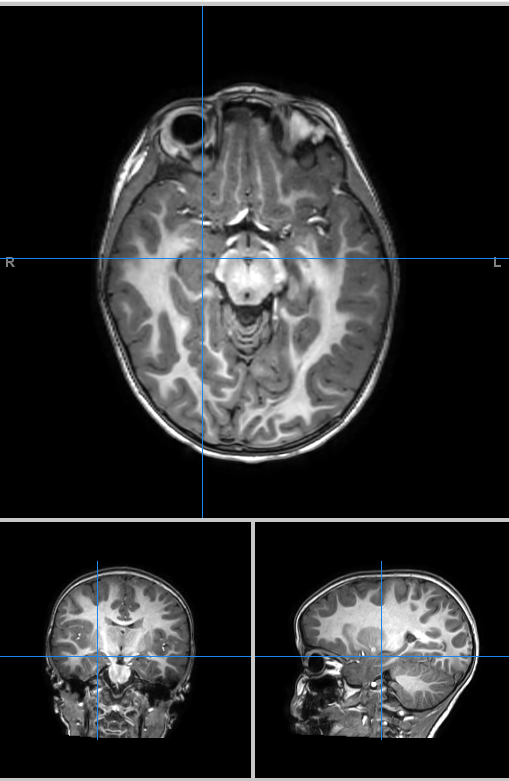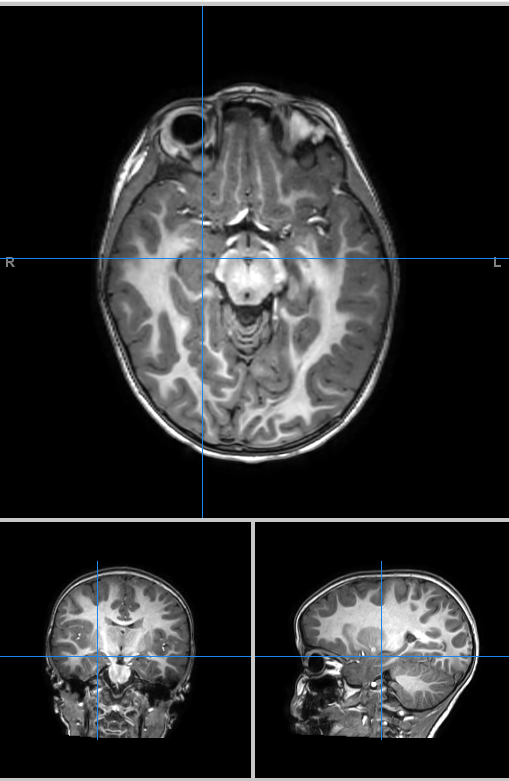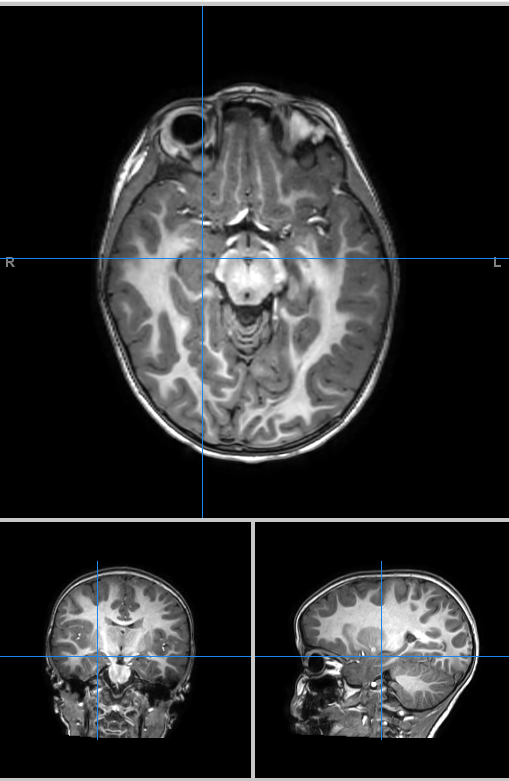 |
| 17 (9) | M | R MT-AT > RF SOZ;  R F subtle region of possible FCD | | 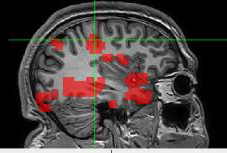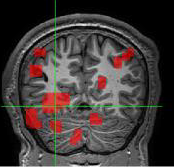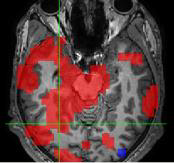 | 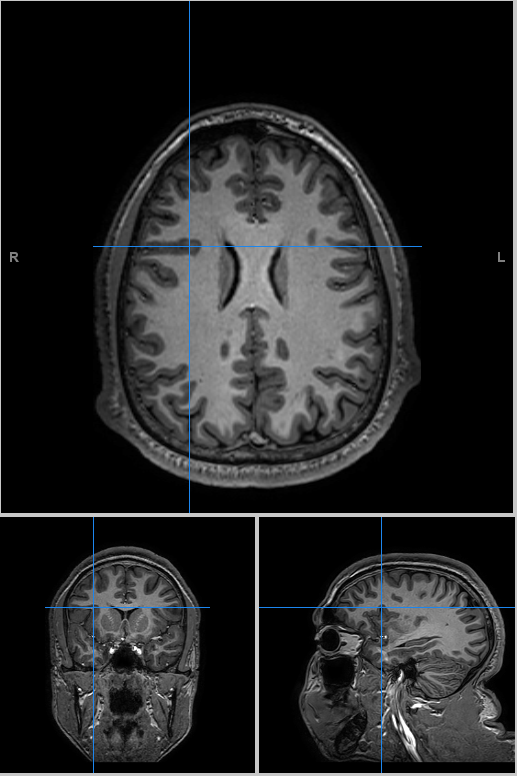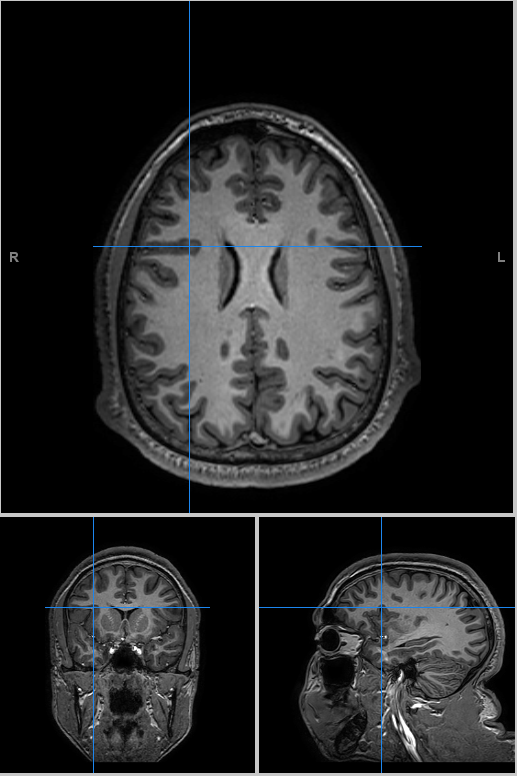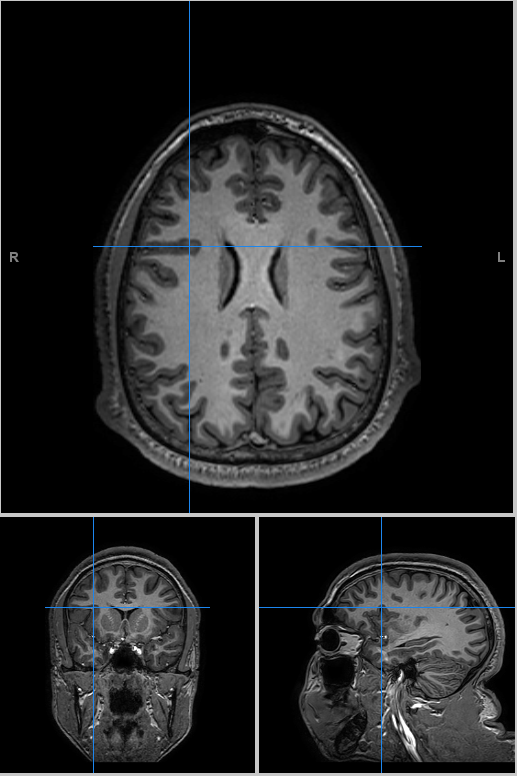 |
| 11 (8) | M | R AT, R F SOZ;  MRI negative | | 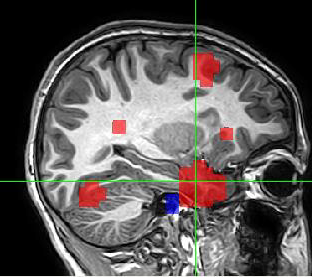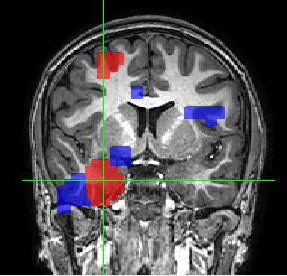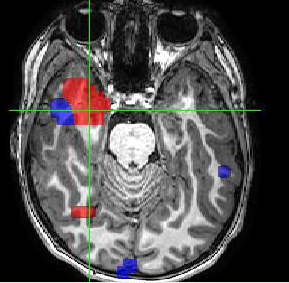 | 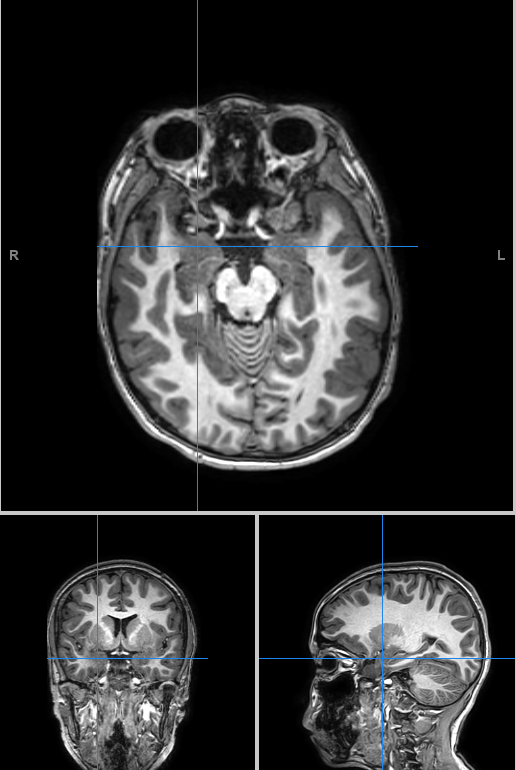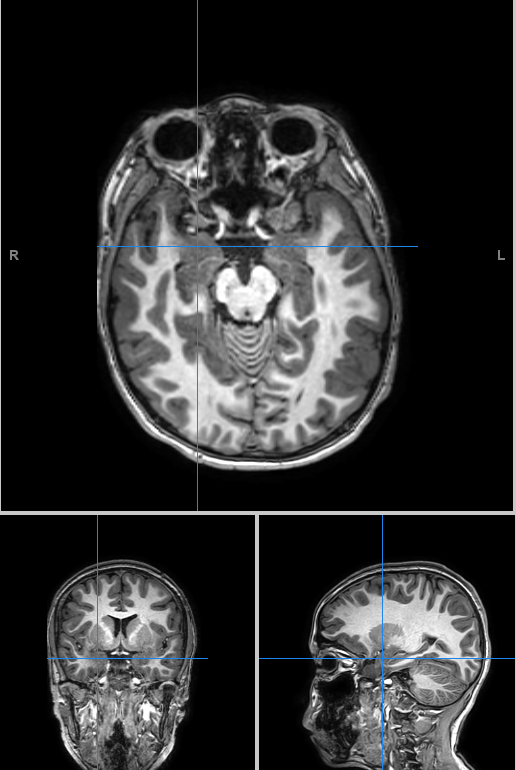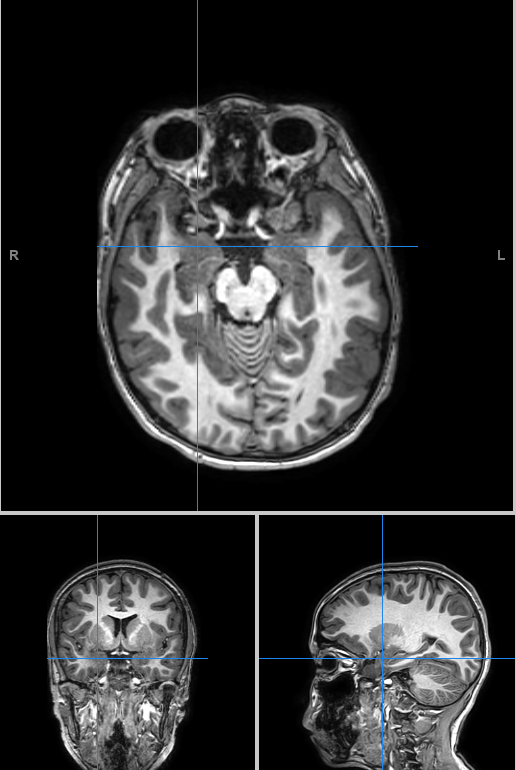 |
