# Supplementary material for: Automated seizure onset zone locator from resting-state functional MRI in drug-resistant epilepsy
Source: Front Neuroimaging. 2023 Jan 4;1:1007668. doi: 10.3389/fnimg.2022.1007668 (PMC10406253; doi:10.3389/fnimg.2022.1007668)
Supplement: Supplementary file 2 [file Table_2.docx]

| 4 (9) | F | R & L Operculum and prefrontal cortex (OPC);  cerebellar region cyst |  | 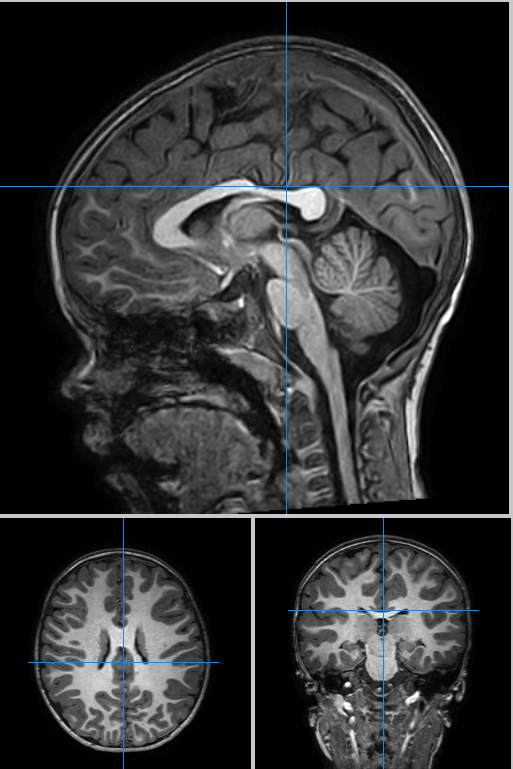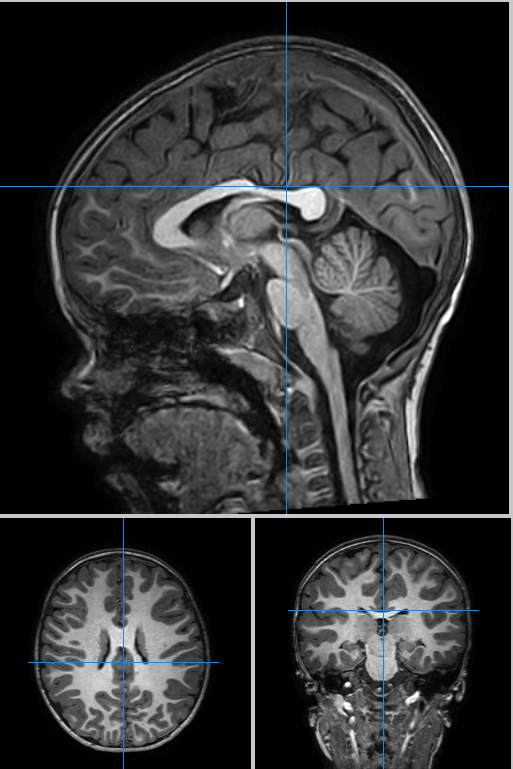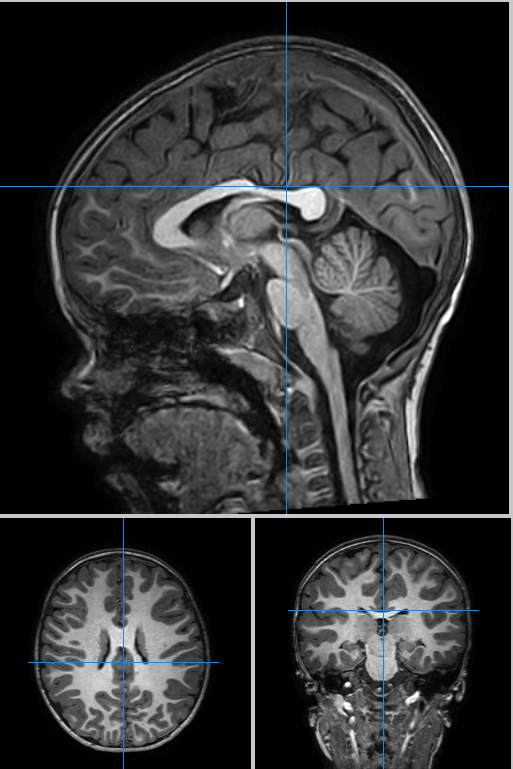 |
| --- | --- | --- | --- | --- |
| 18 (1) | F | R motor, Bilateral (B) Premotor Cortex (PMC);  PFC polymicrogyria | 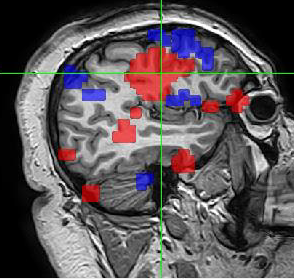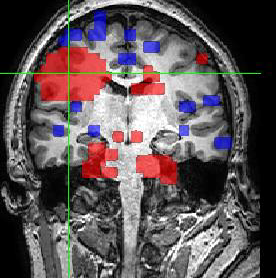 | 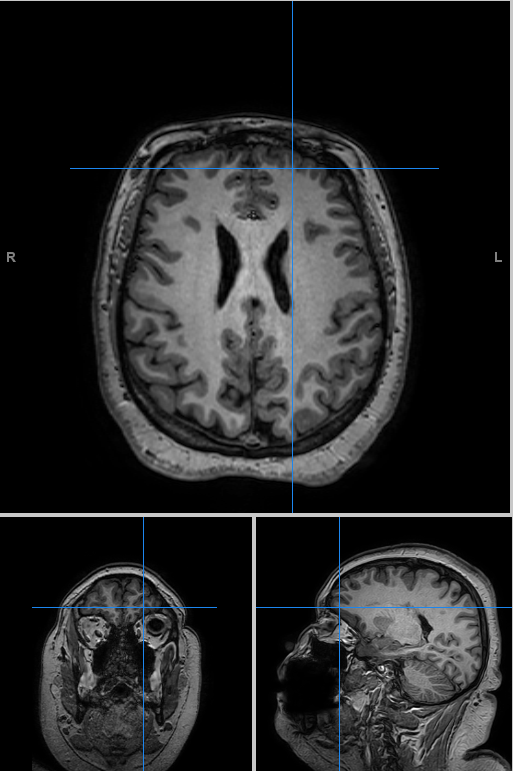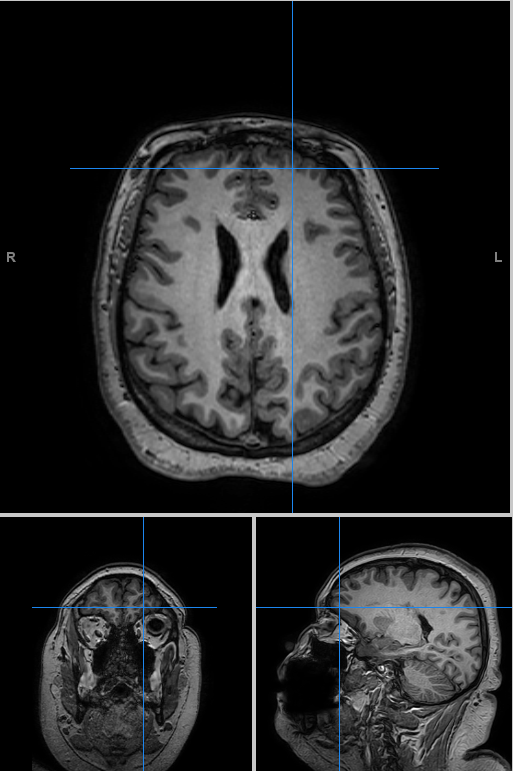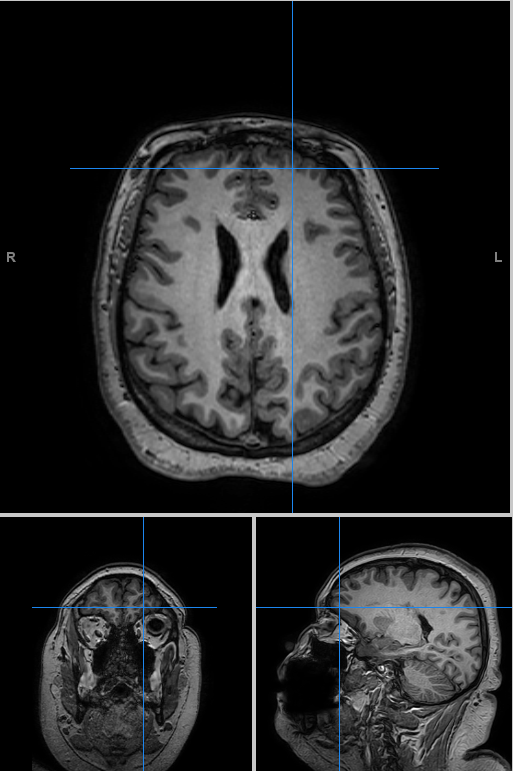 |
| 10 (5) | F | L AT-MT SOZ;  MRI negative |  | 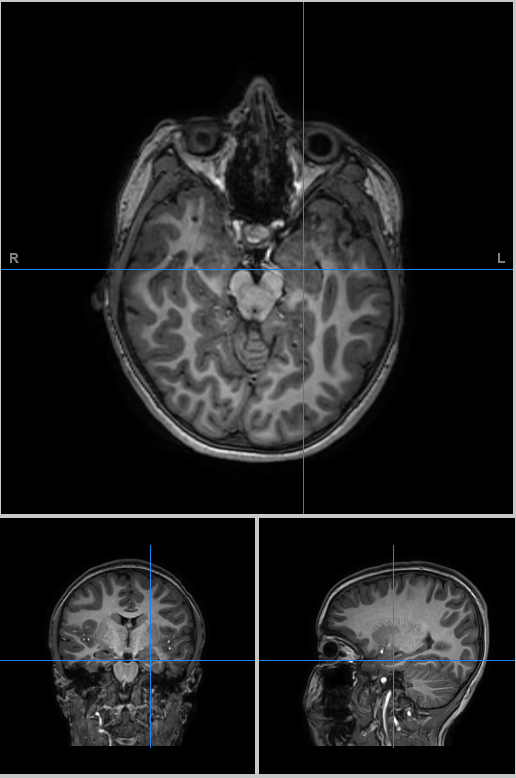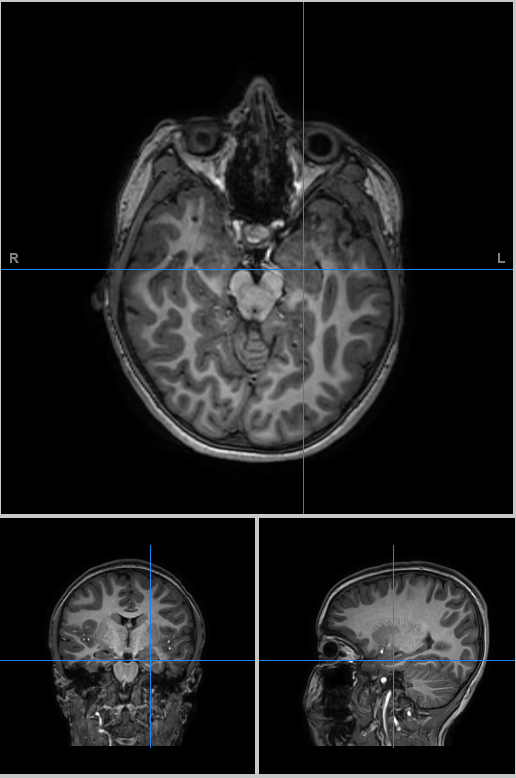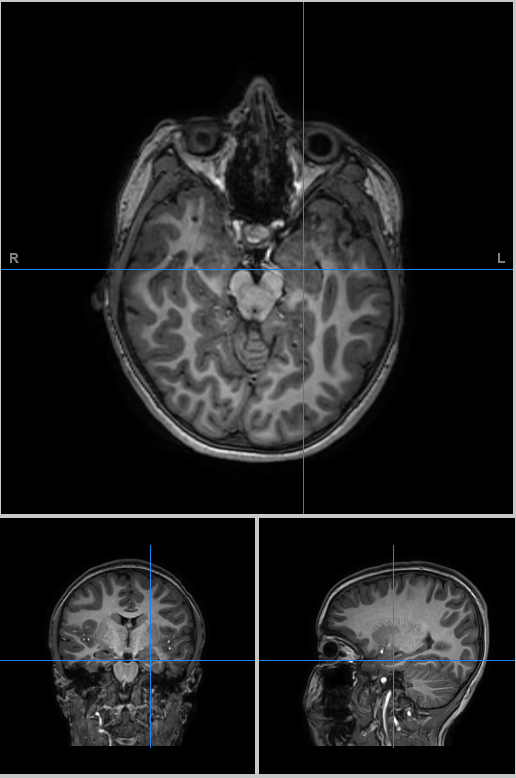 |
| 13(8) | M | B AT/MT;  MRI negative |  | 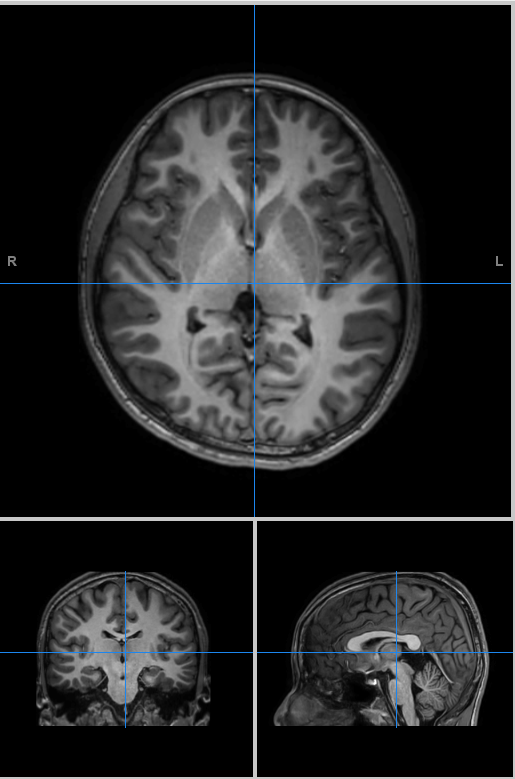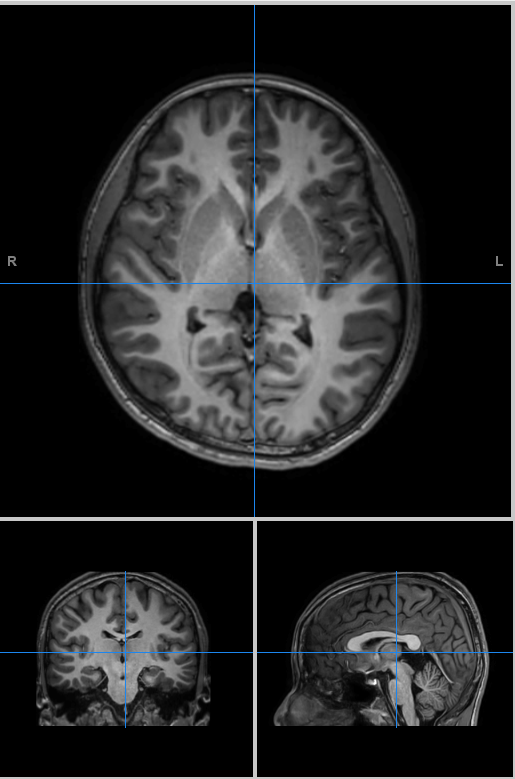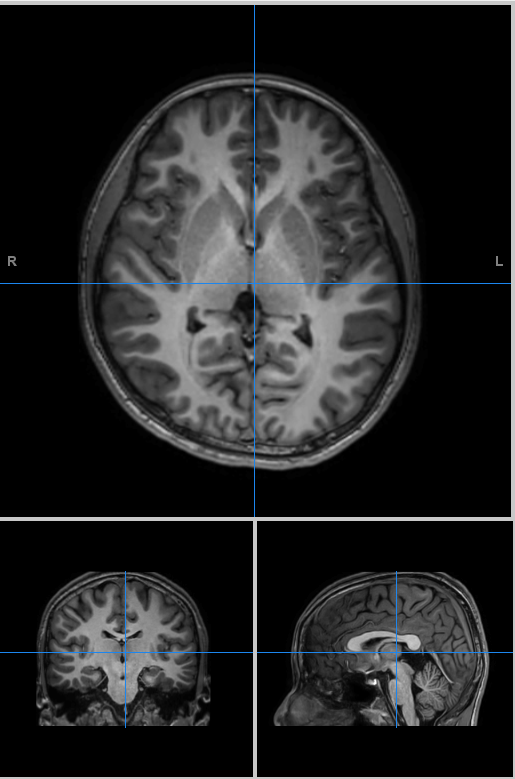 |
| 2(7) | F | multifocal, left and right, Abnormal Motor RSN;  Posterior dysgenesis of the corpus callosum and bilateral posterior greater, | 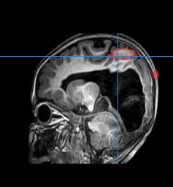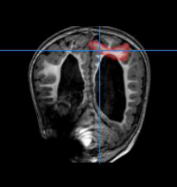 | 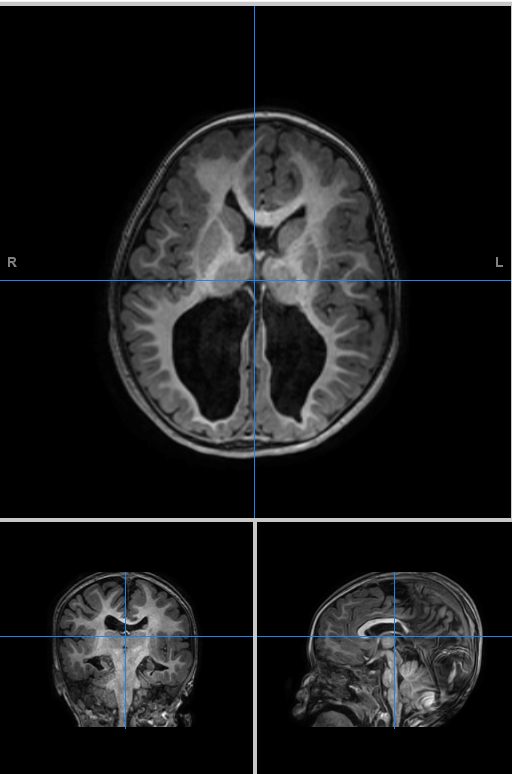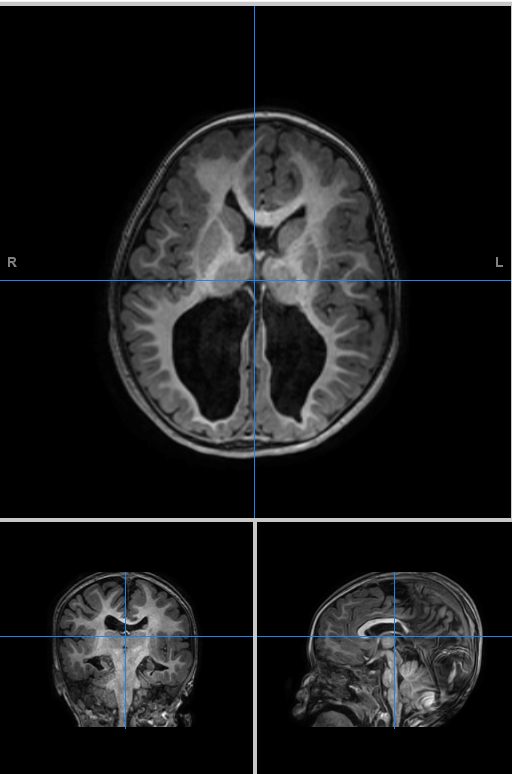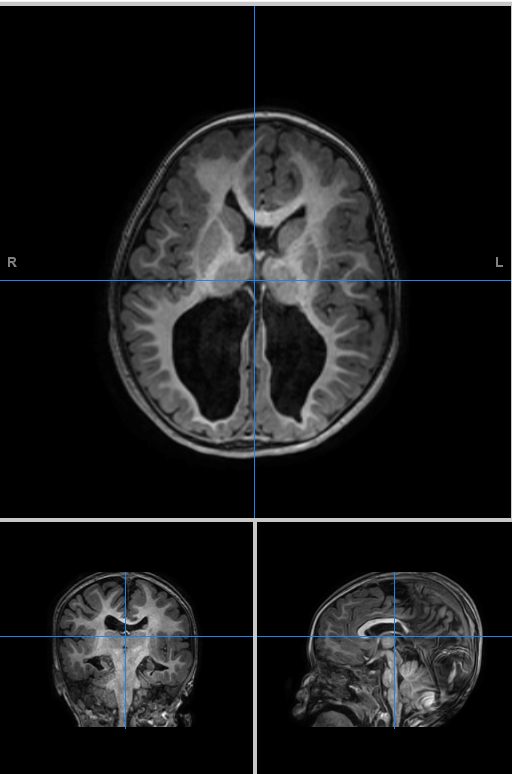 |
| 2(7) | M | L & R PFC downstream connectivity disruption;  hypothalamic hamartoma (HH) |  | 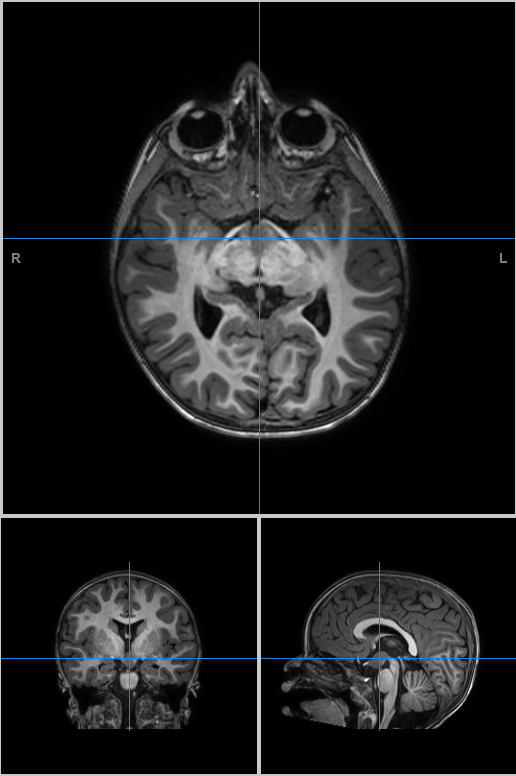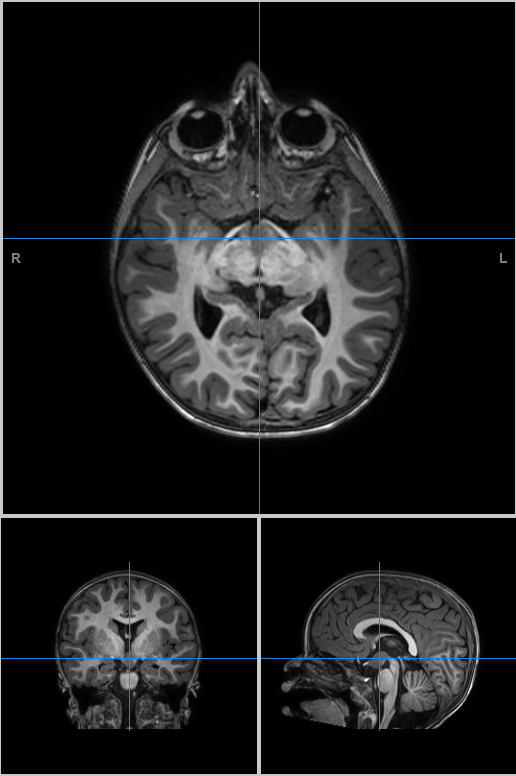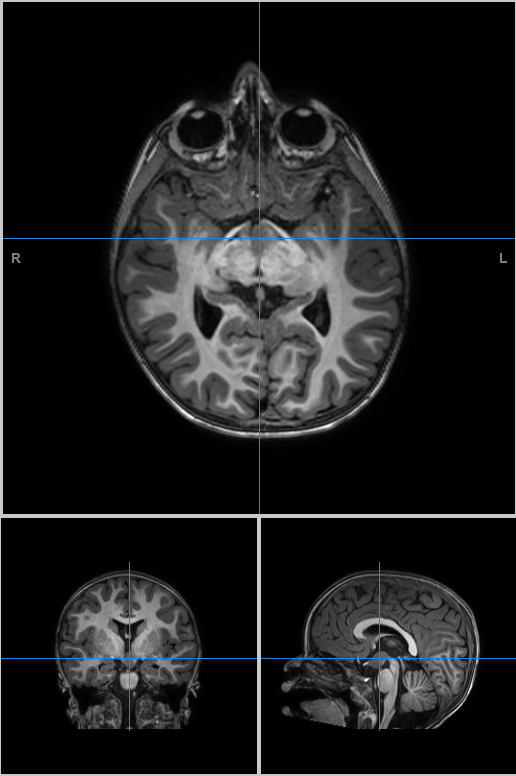 |
| 0(3) | F | B PFC superior lesion;  R anterior cingulate gyrus (ACG) FCD, dysagenesis corpus callosum |  | 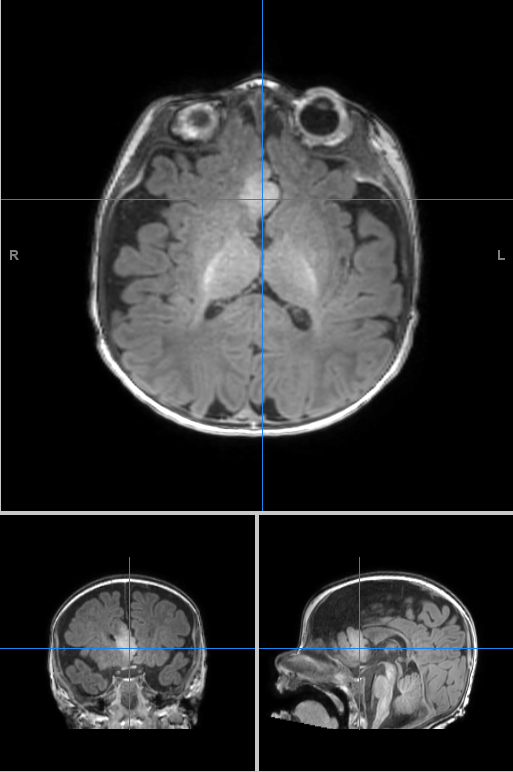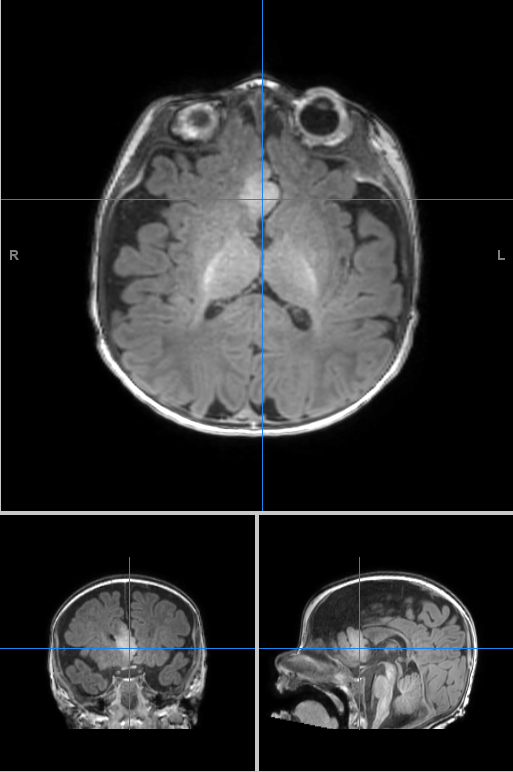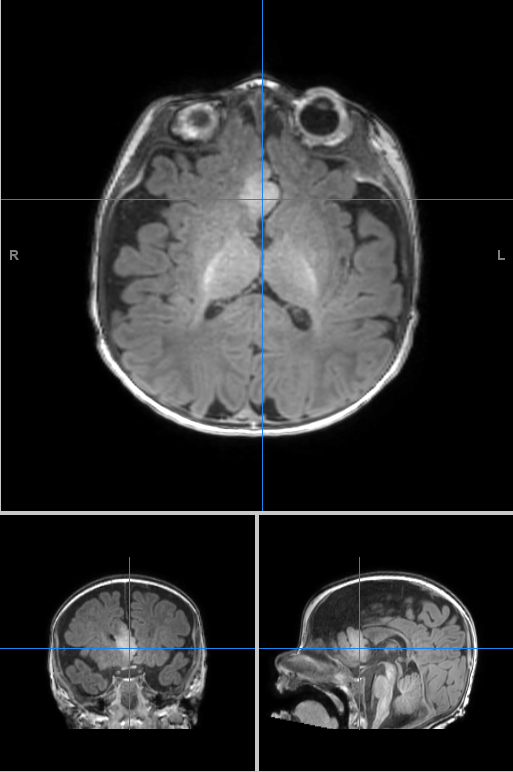 |
| 2(10) | F | R F-T, lateral occipital, lesser P ;  R posterior & anterior megancephally |  | 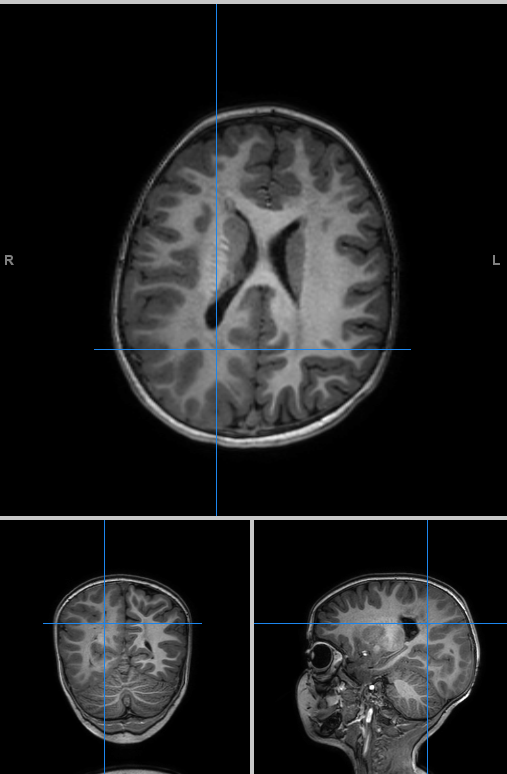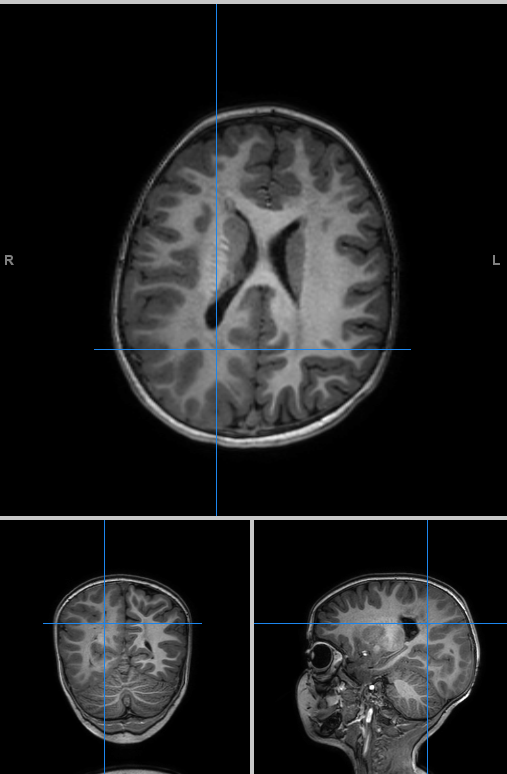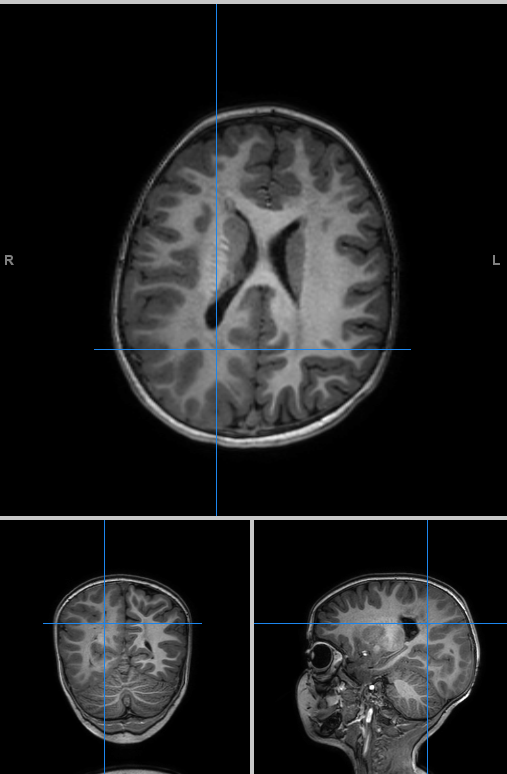 |
| 2(11) | M | R PFC & R AT SOZ;  R PFC congenital malformation |  | 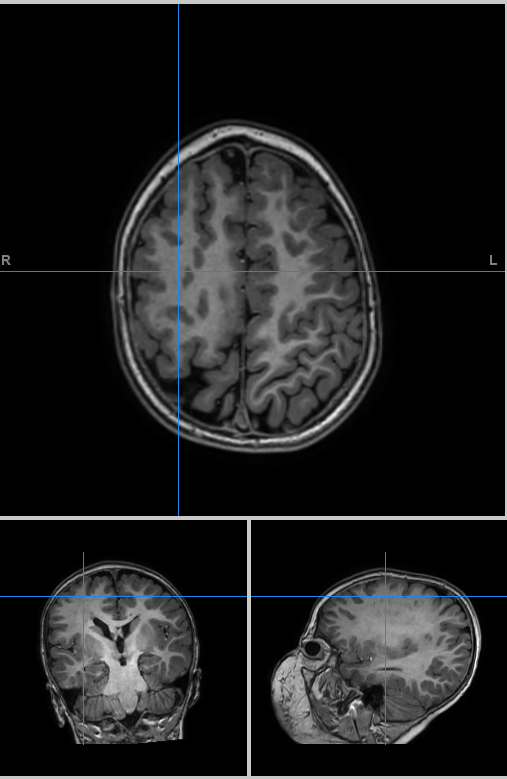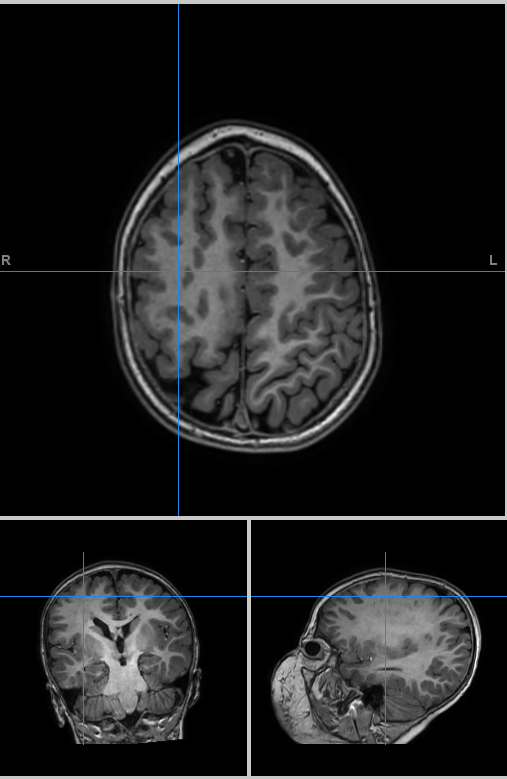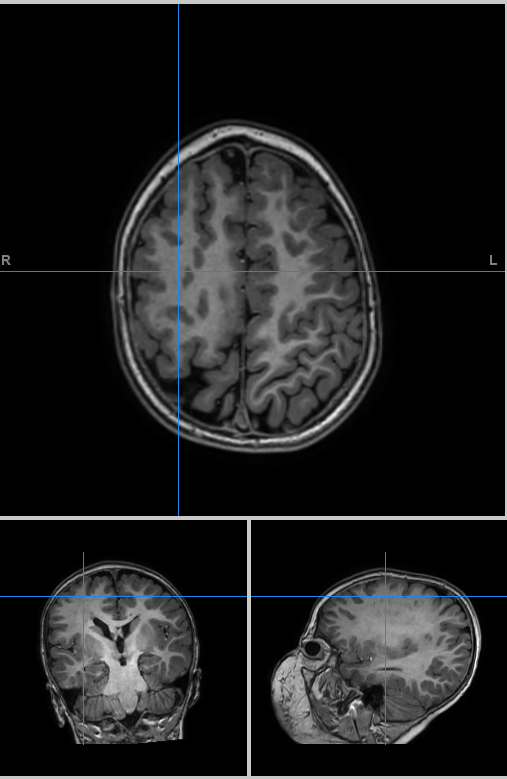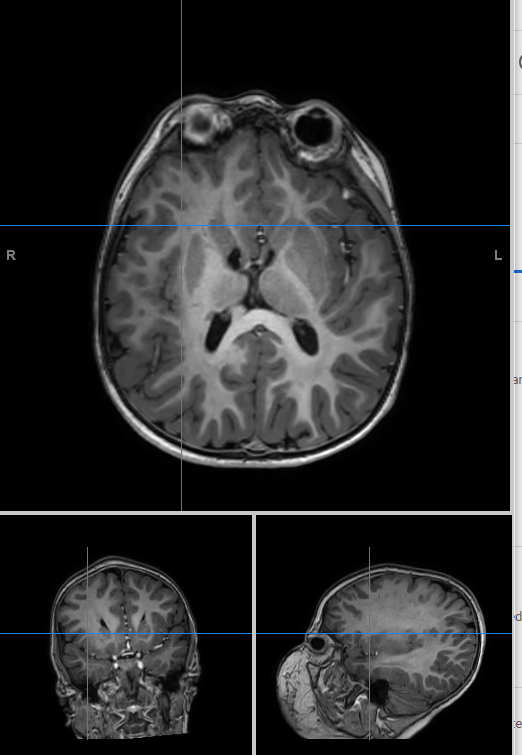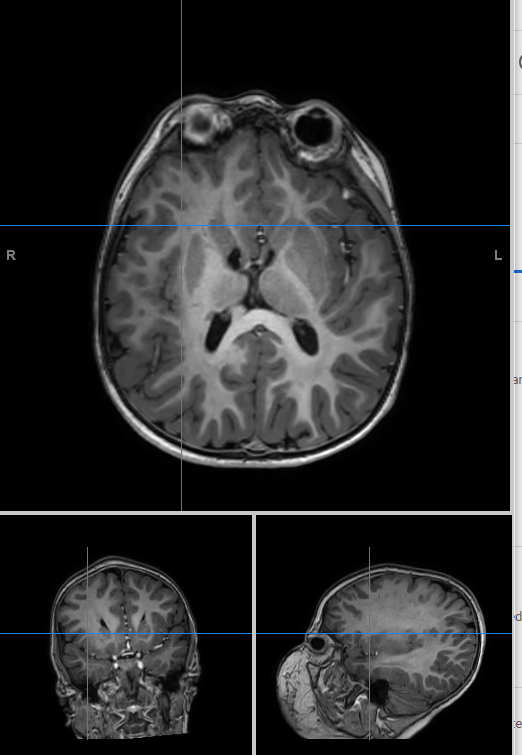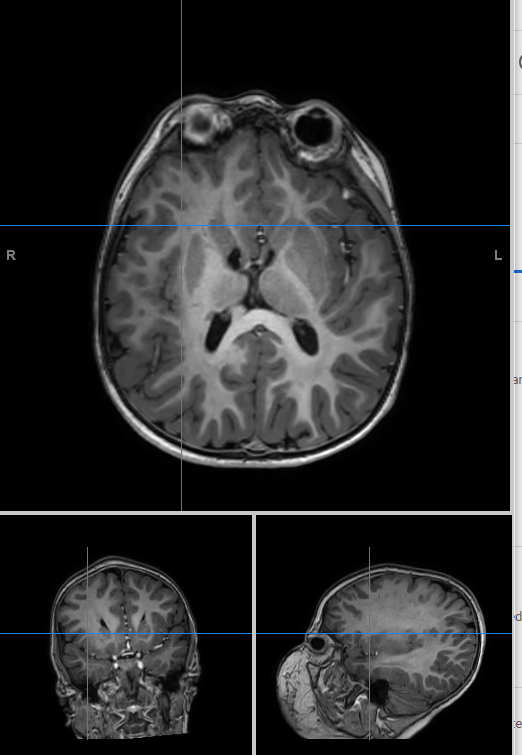 |
| 2(1) | F | R AT-MT;  R frontopontine tract (FPT) congenital malformation |  |  |
| 3(6) | M | L T & P & F broad SOZ; negative MRI |  | 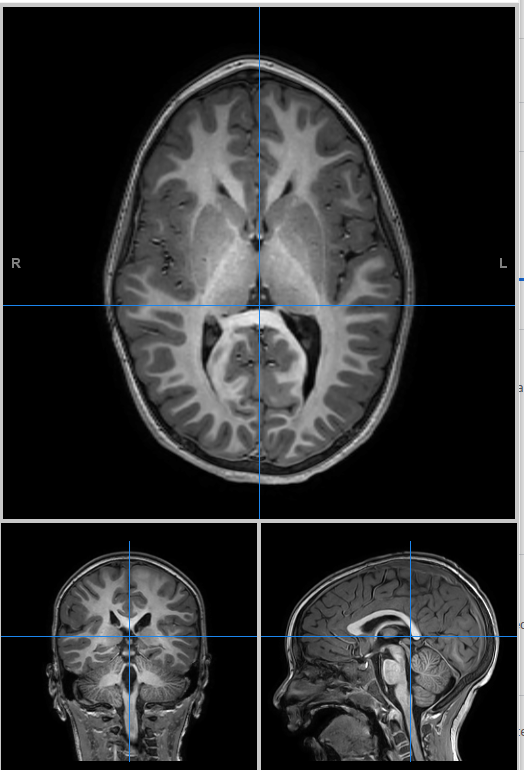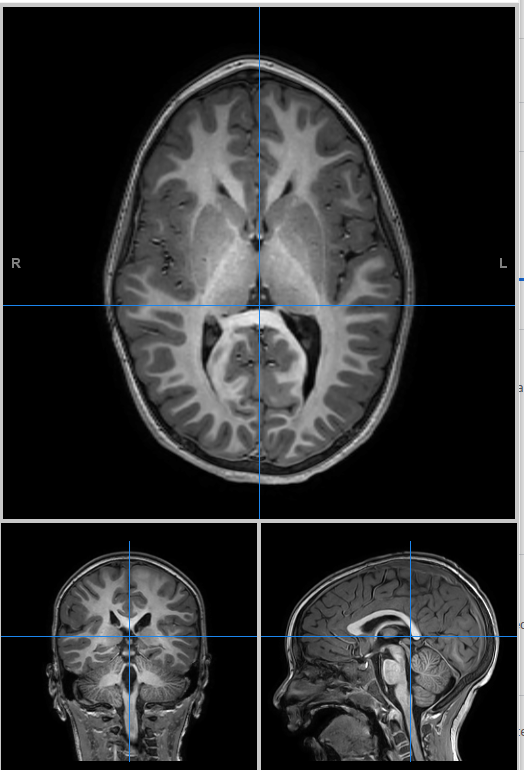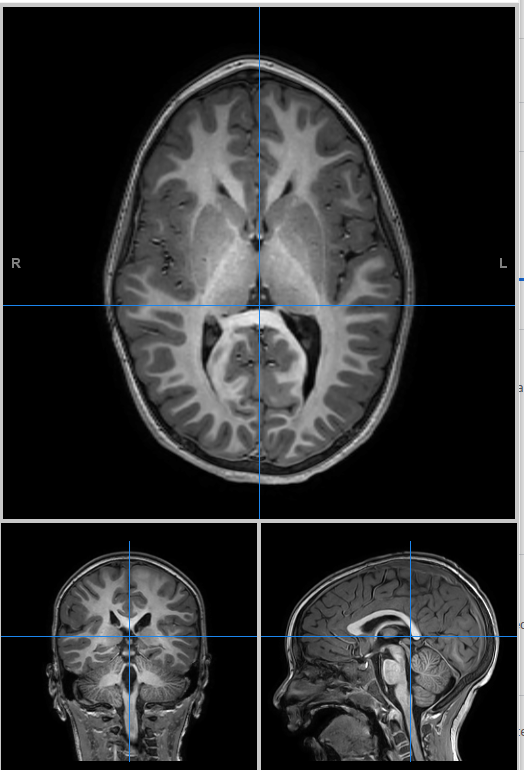 |
| 1(4) | F | L T SOZ ;  L T congenital malformation |  | 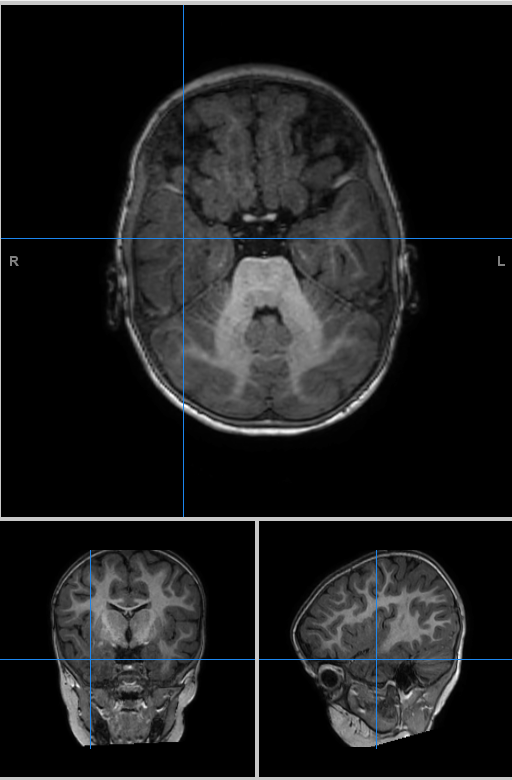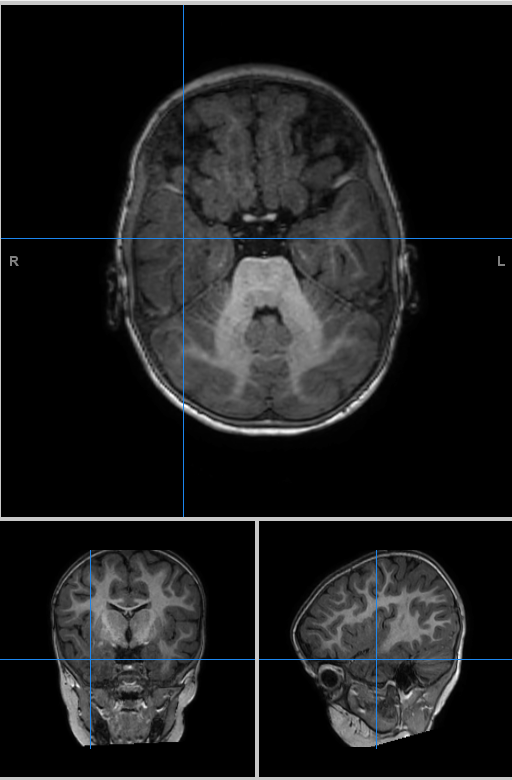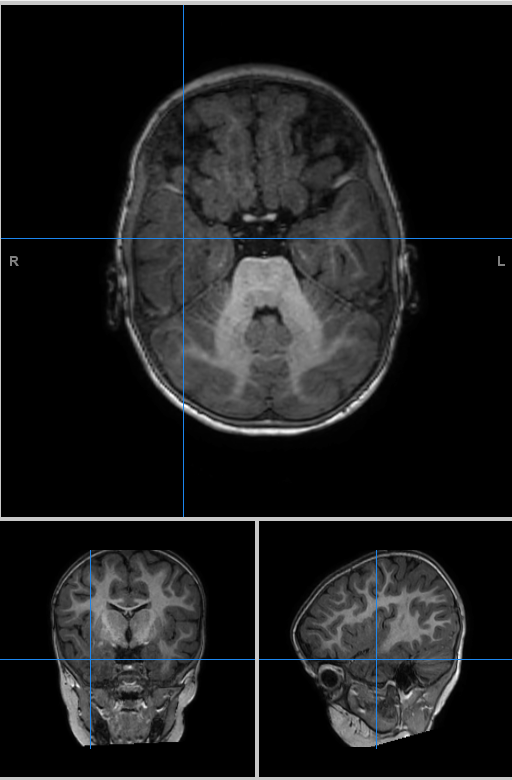 |
| 9(11) | M | L deep grey, OPC, Inferior Frontal Gyrus (IFG) & R; negative MRI |  | 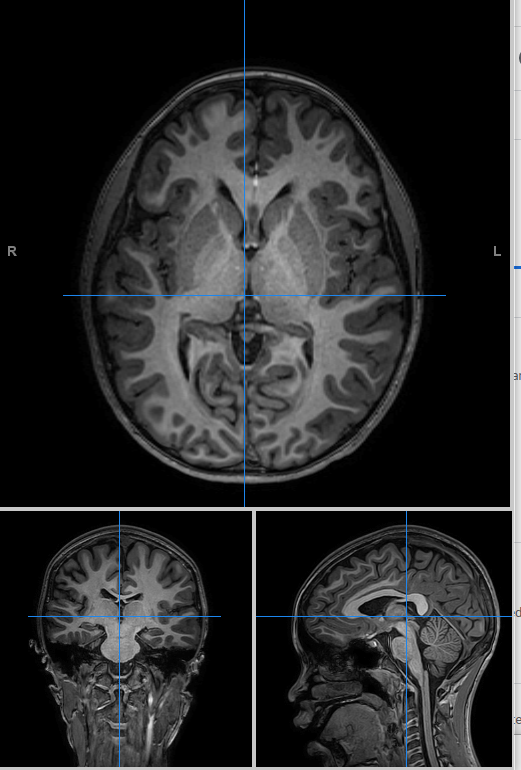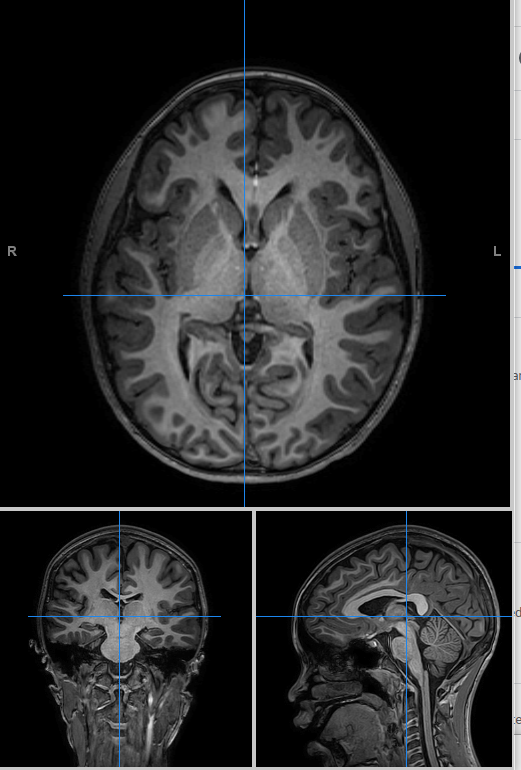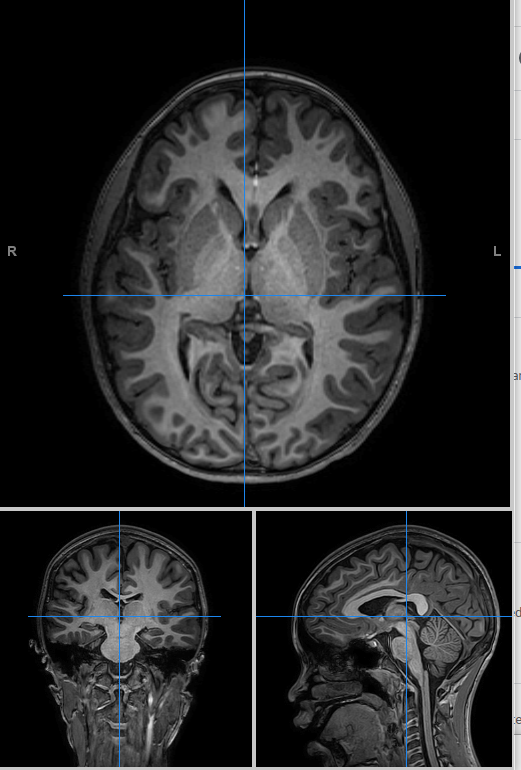 |
| 16(5) | M | R Frontal OPC; Negative MRI |  | 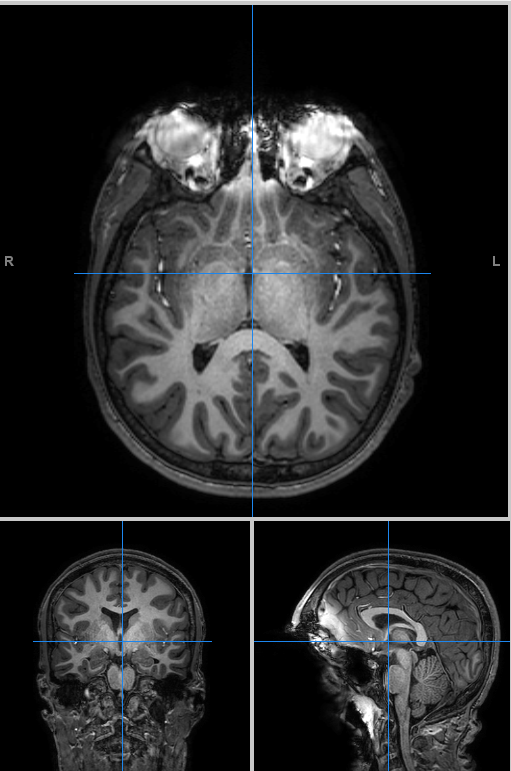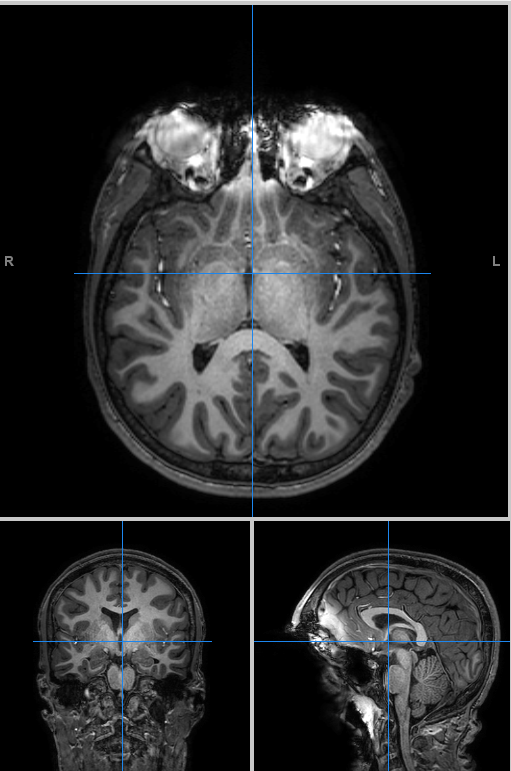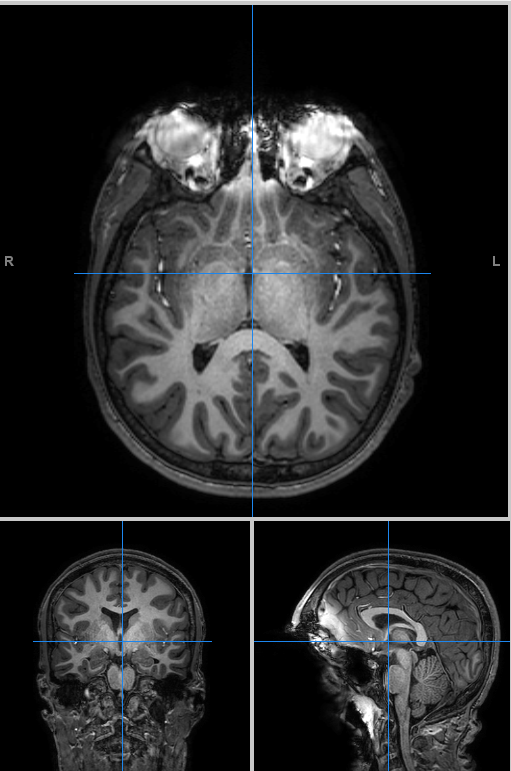 |
| 13(1) | M | L F SOZ;  MRI negative |  | 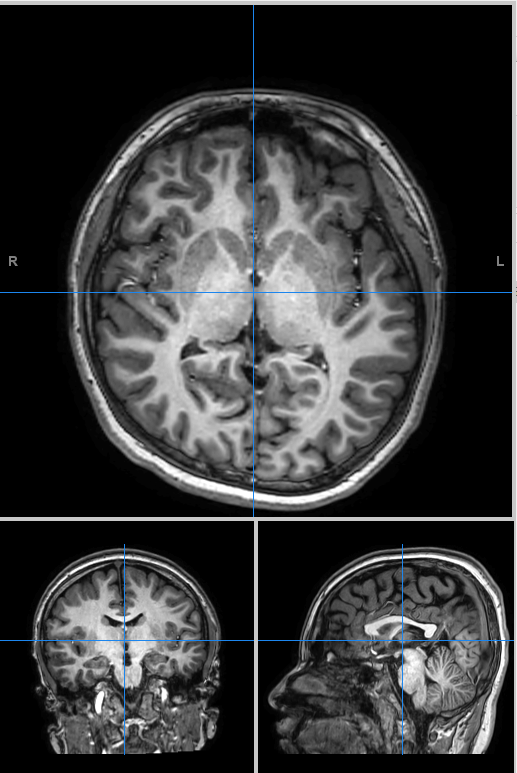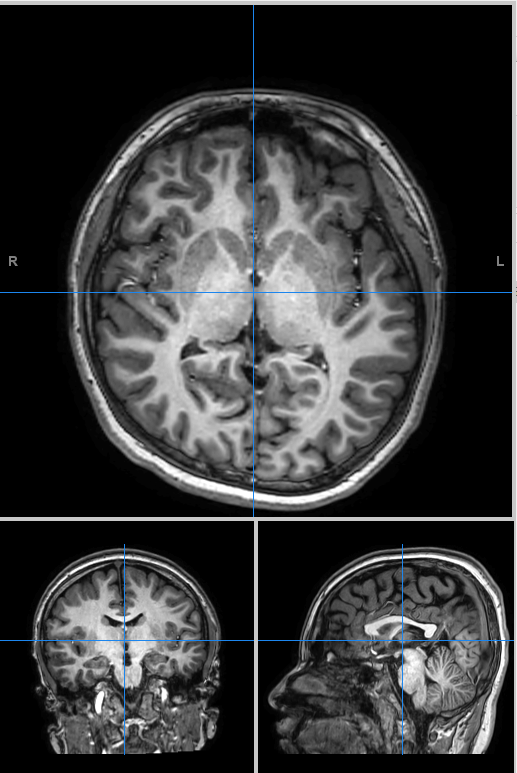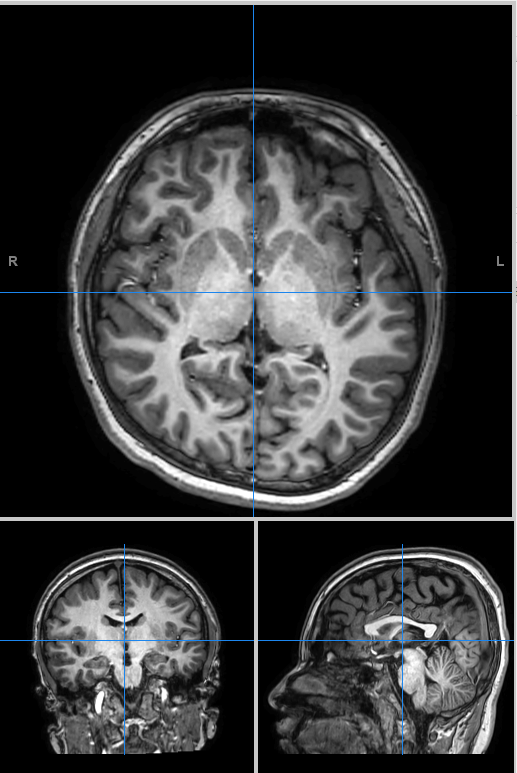 |
| 9(0) | F | B M T SOZ;  Generalized atrophy |  | 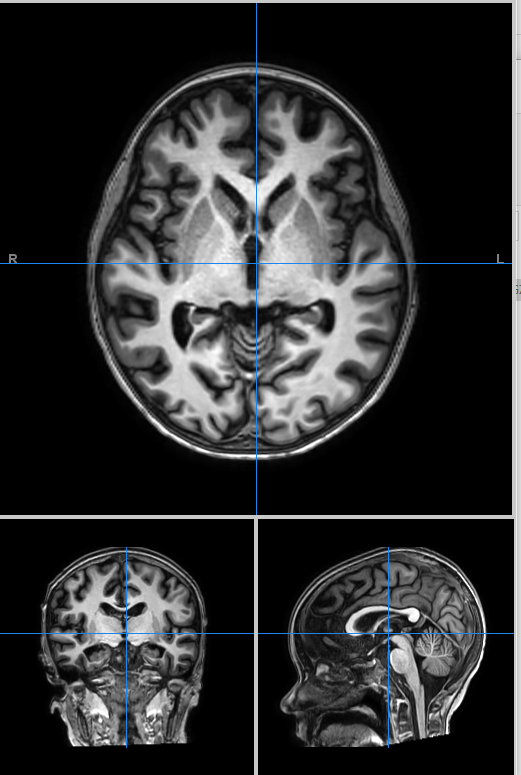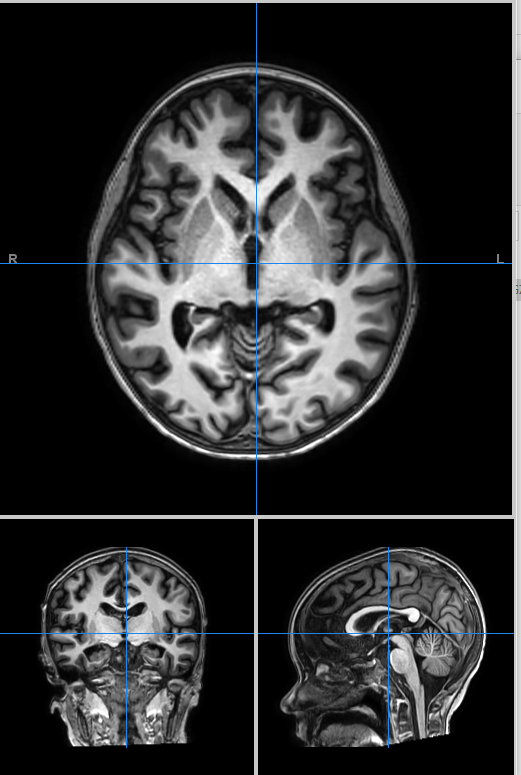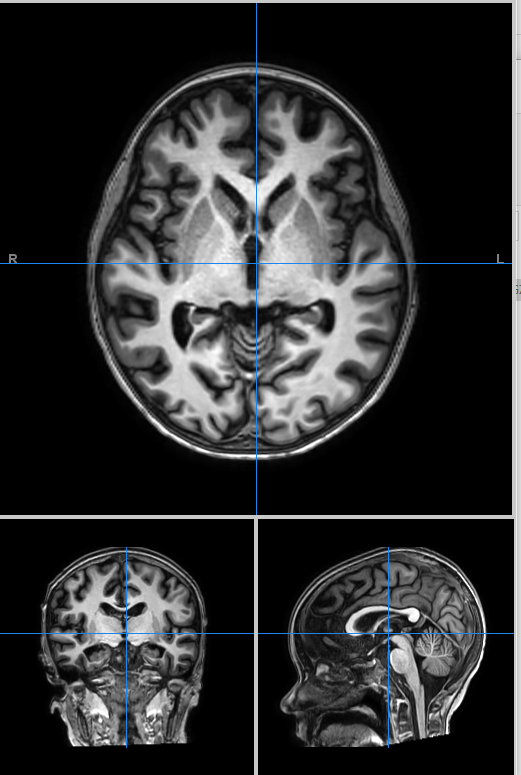 |
| 3(4) | F | L& R PMC;  B multifocal FPT tuberous sclerosis |  | 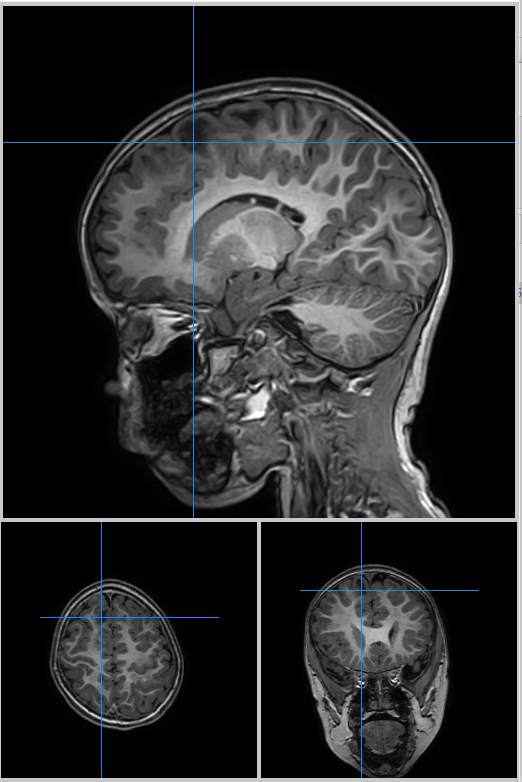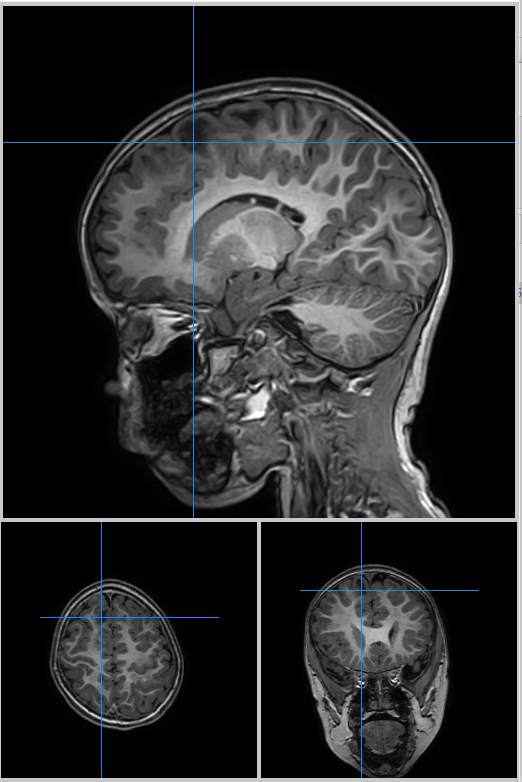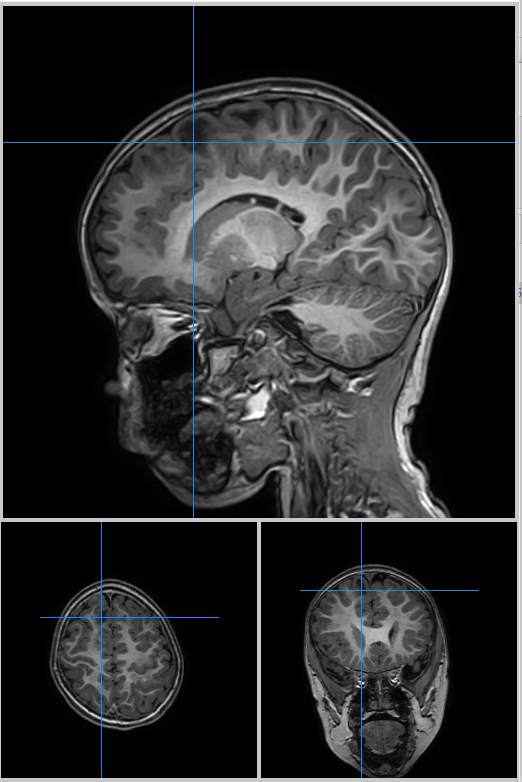 |
| 11(11) | F | R & L MT-AT;  R MT atypical configuration |  | 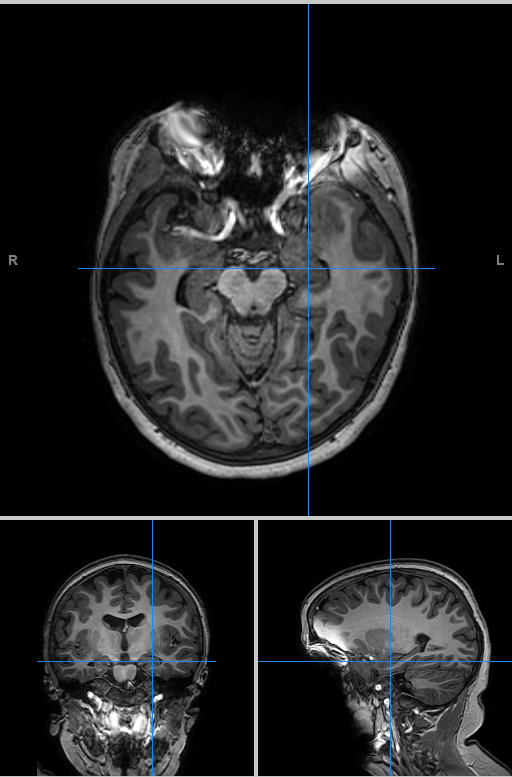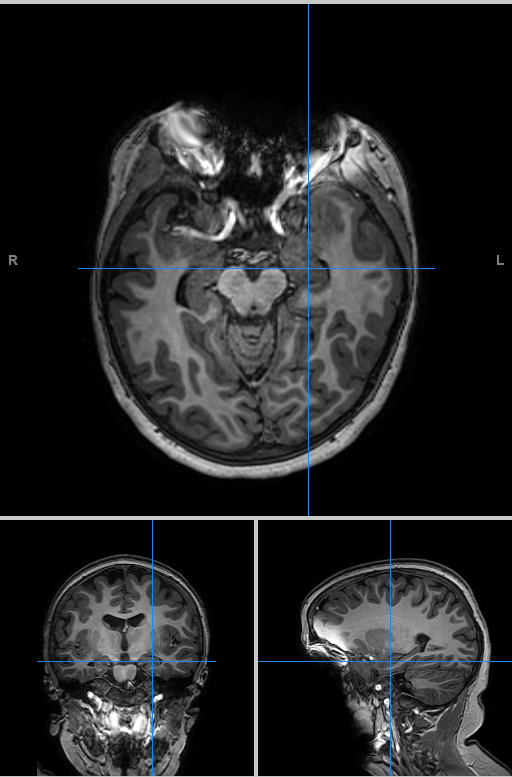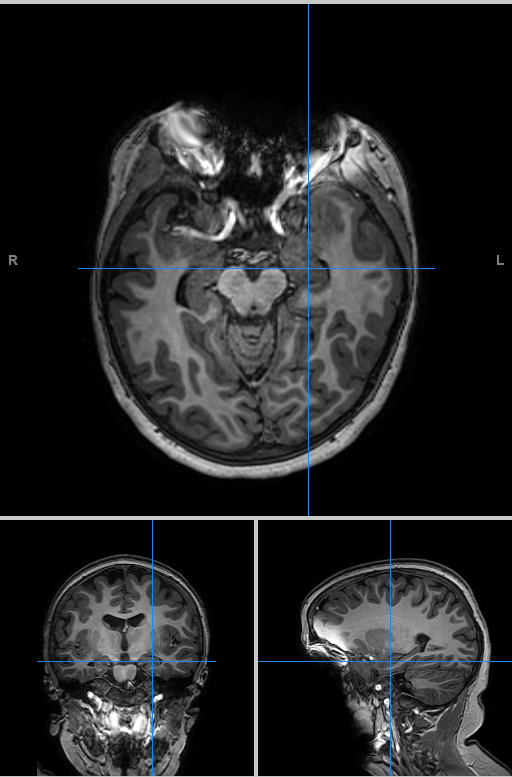 |
