# Supplementary material for: Automated seizure onset zone locator from resting-state functional MRI in drug-resistant epilepsy
Source: Front Neuroimaging. 2023 Jan 4;1:1007668. doi: 10.3389/fnimg.2022.1007668 (PMC10406253; doi:10.3389/fnimg.2022.1007668)
Supplement: Supplementary file 3 [file Table_3.docx]

| 12(8) | M | B PFC;  MRI negative |  | 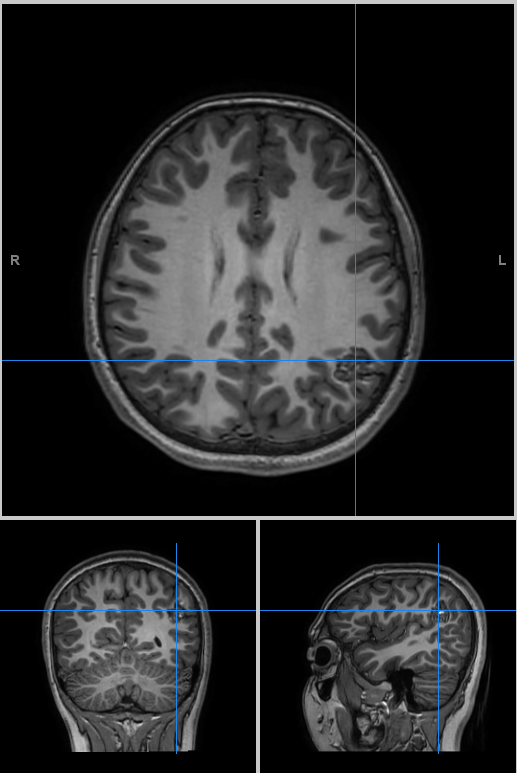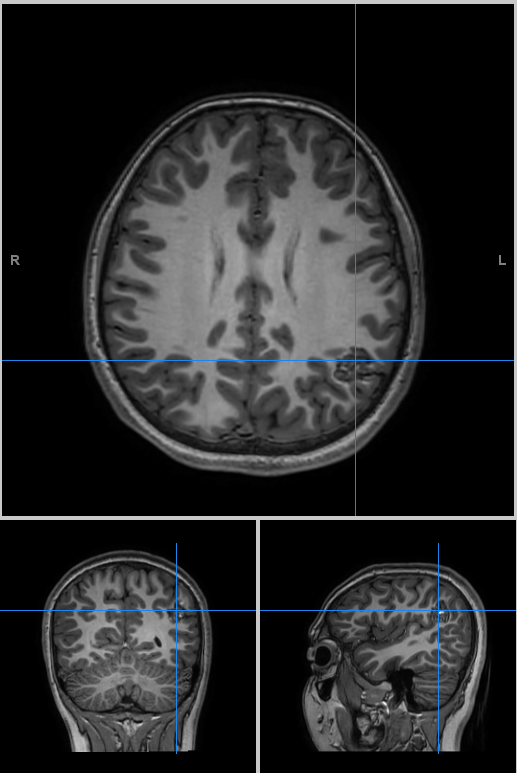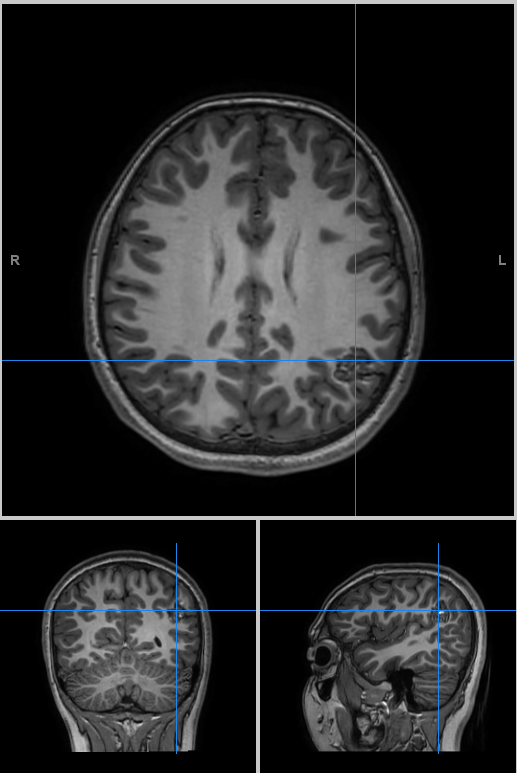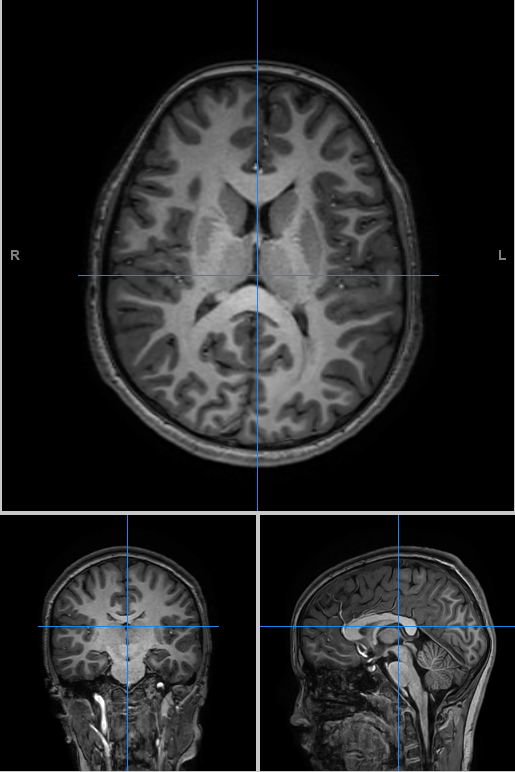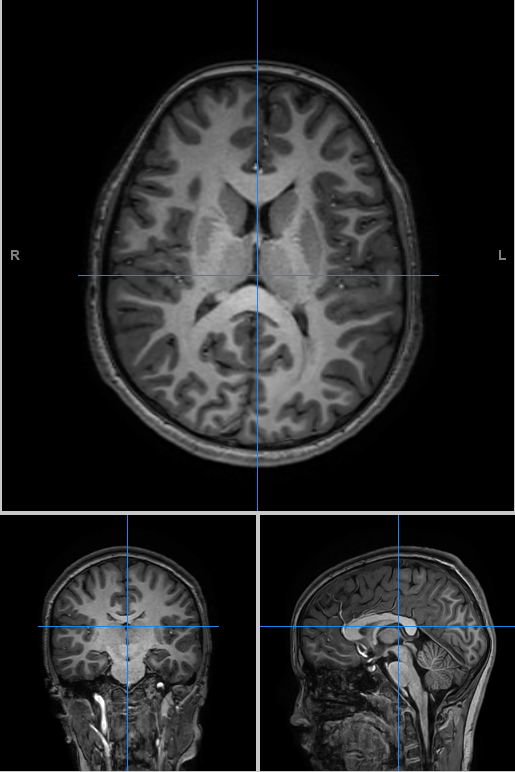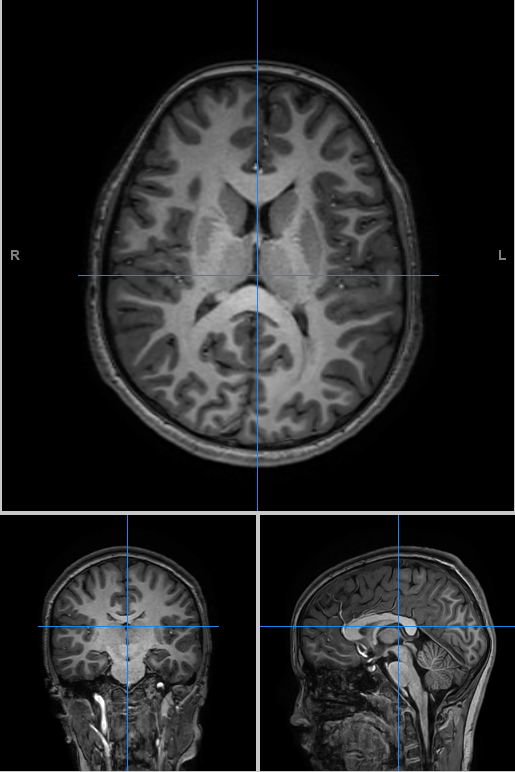 |
| --- | --- | --- | --- | --- |
| 12(7) | F | L P peri-lesional; L P FCD |  |  |
| 12(6) | M | R Temporal (T) Occipital (O) White matter (WM) deep SOZ;  MRI negative |  | 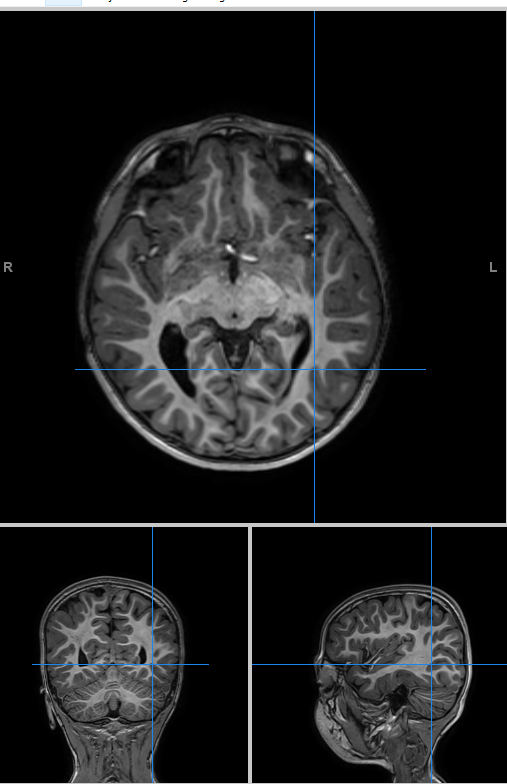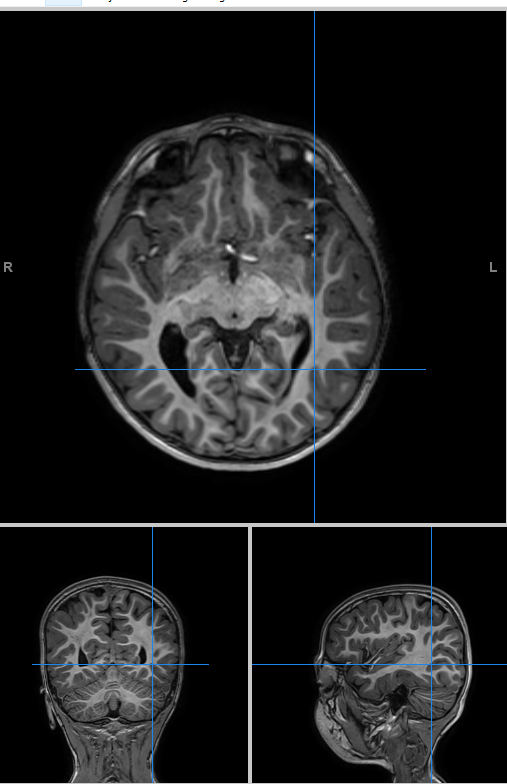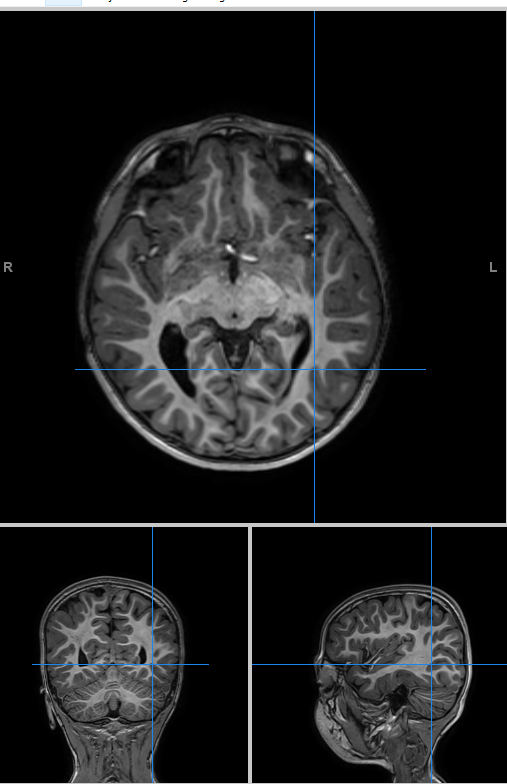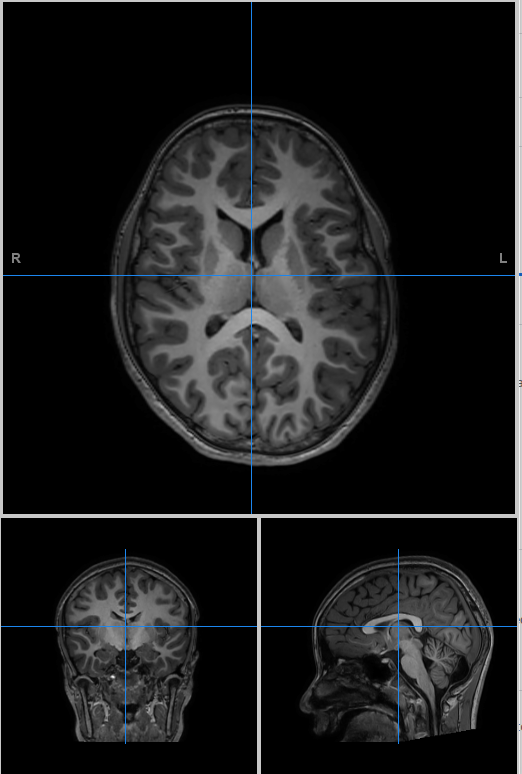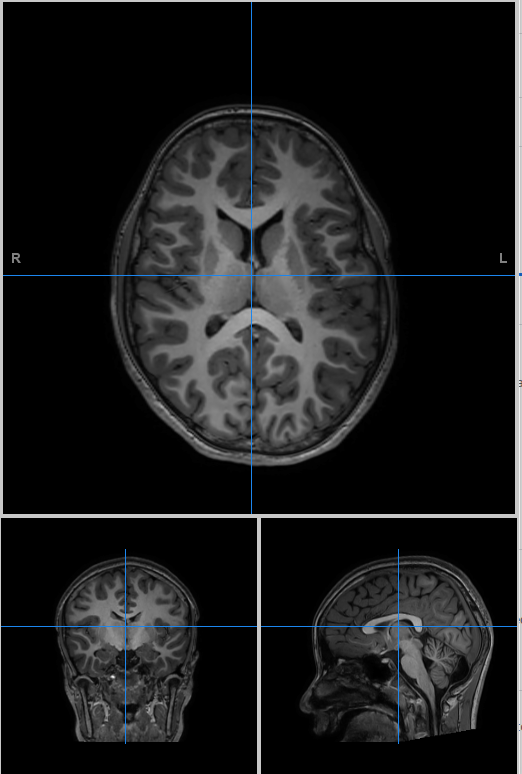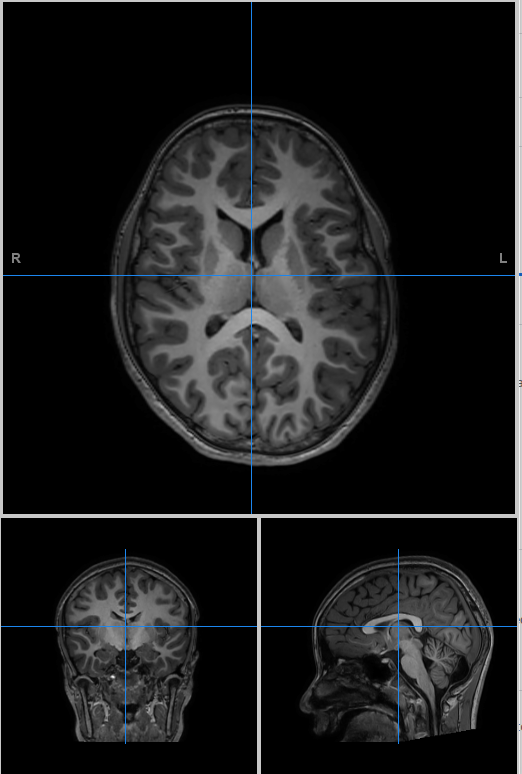 |
| 4(6) | M | L O-T SOZ;  L TO FCD |  |  |
| 0(7) | M | R AT SOZ; Global white matter atrophy |  | 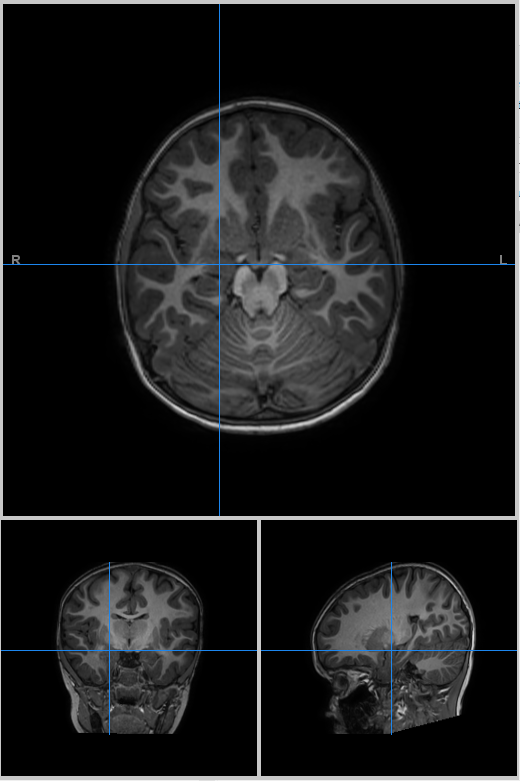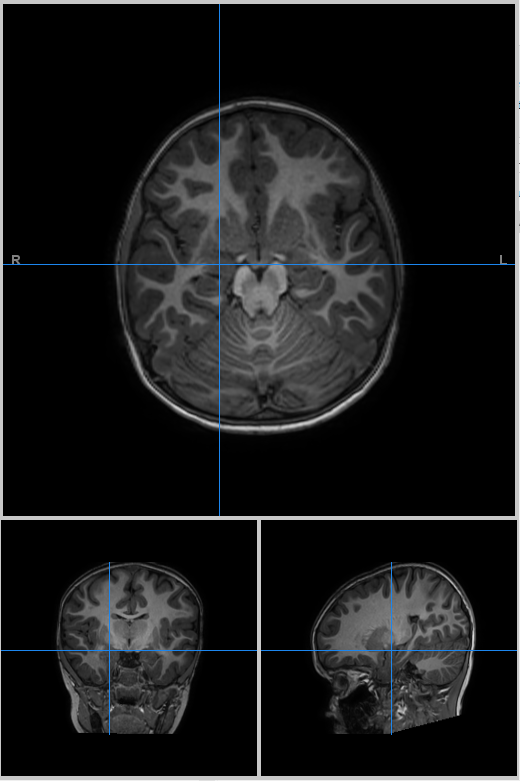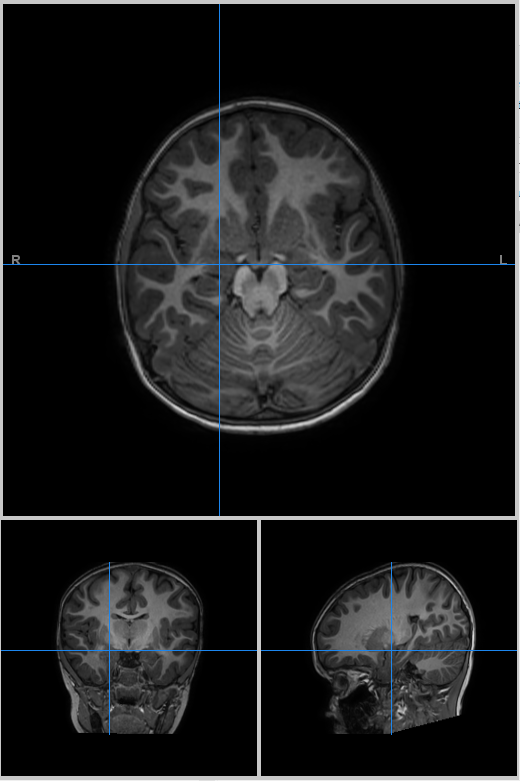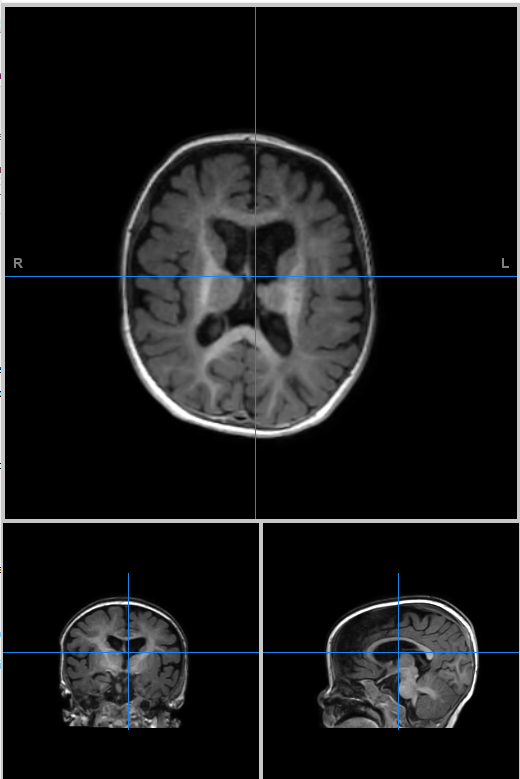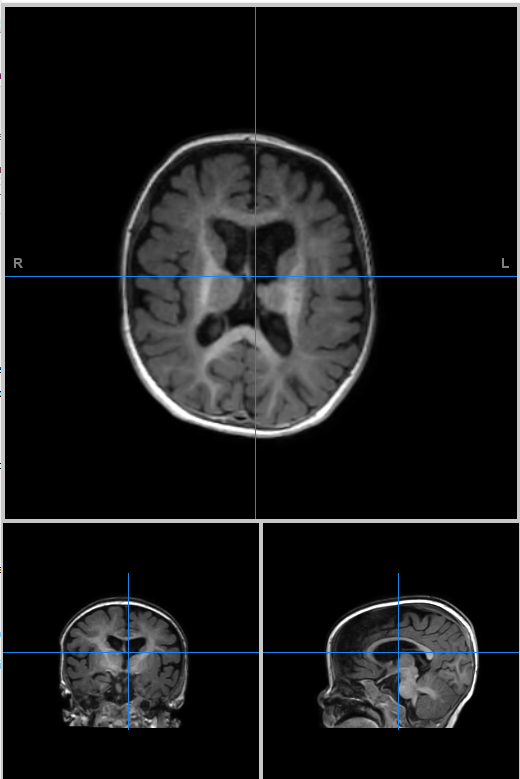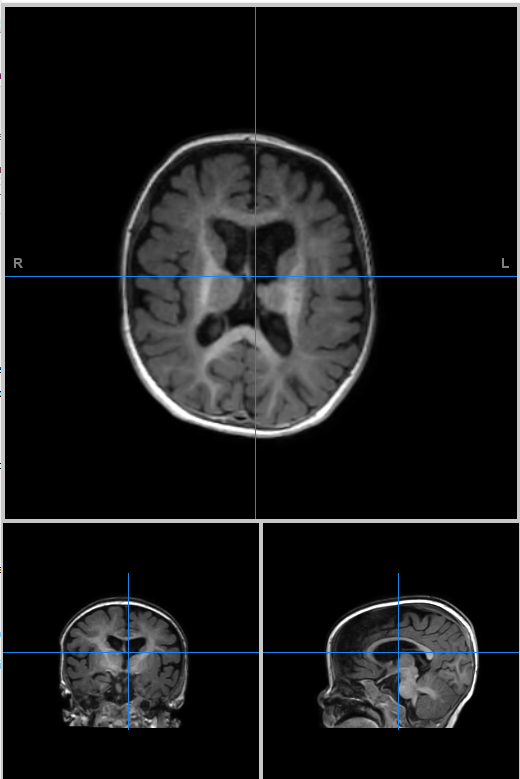 |
| 5(3) | F | R MT;  MRI negative |  | 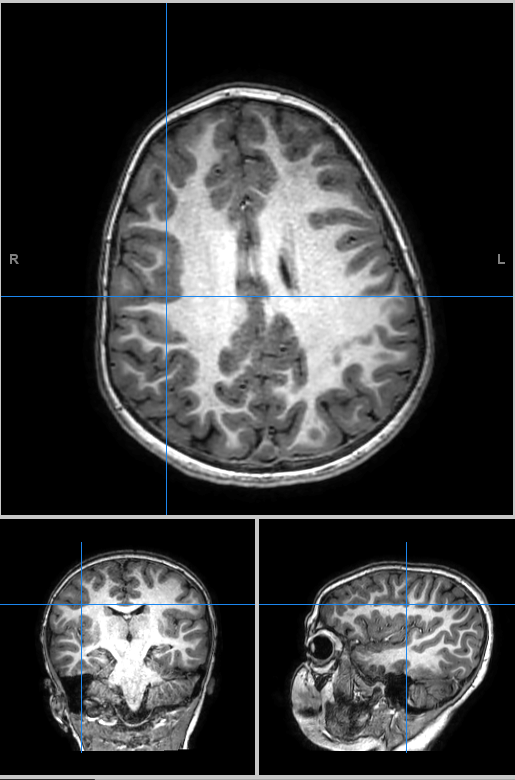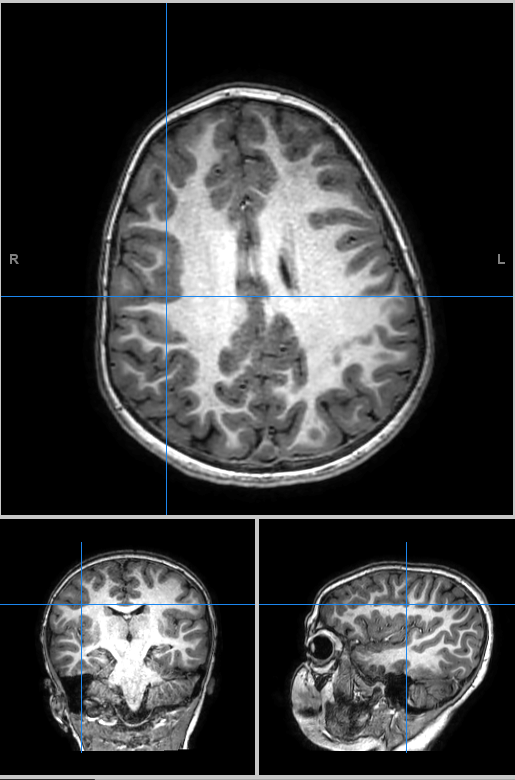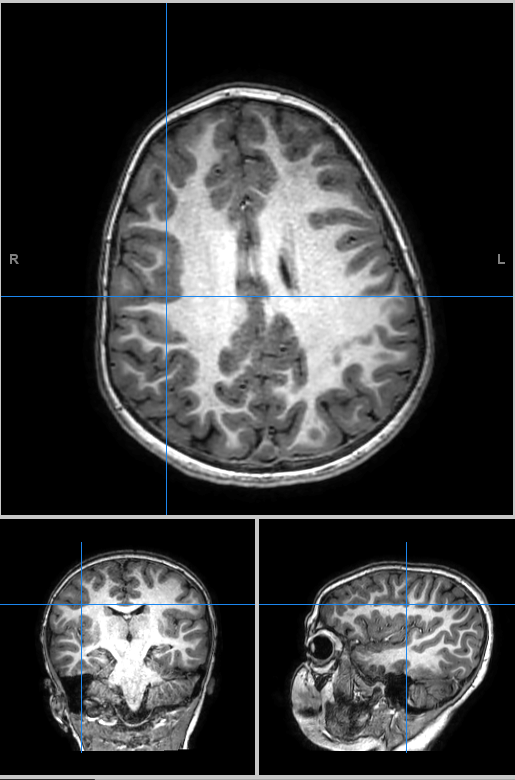 |
| 5(7) | M | R FP SOZ;  R F FCD |  |  |
| 1(5) | F | L T SOZ;  L T FCD |  | 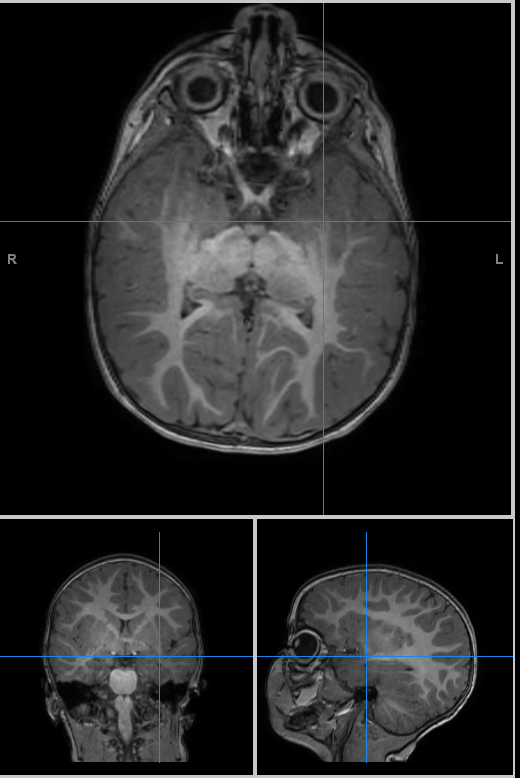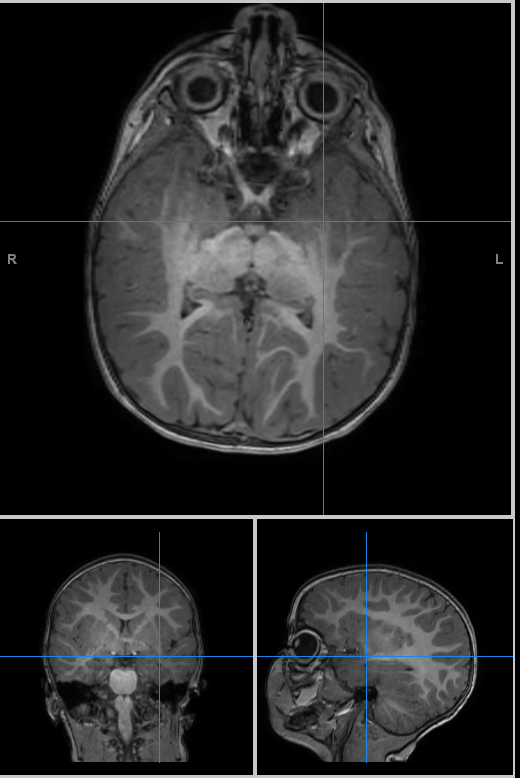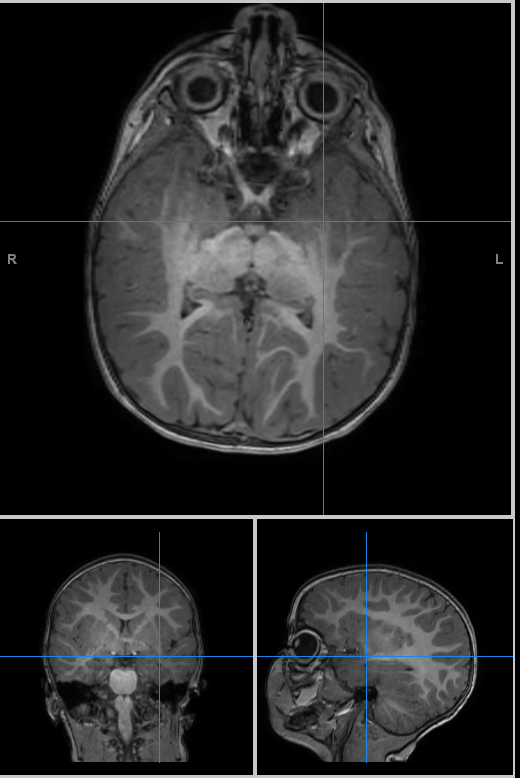 |
| 12(10) | M | R TO SOZ;  MRI negative |  | 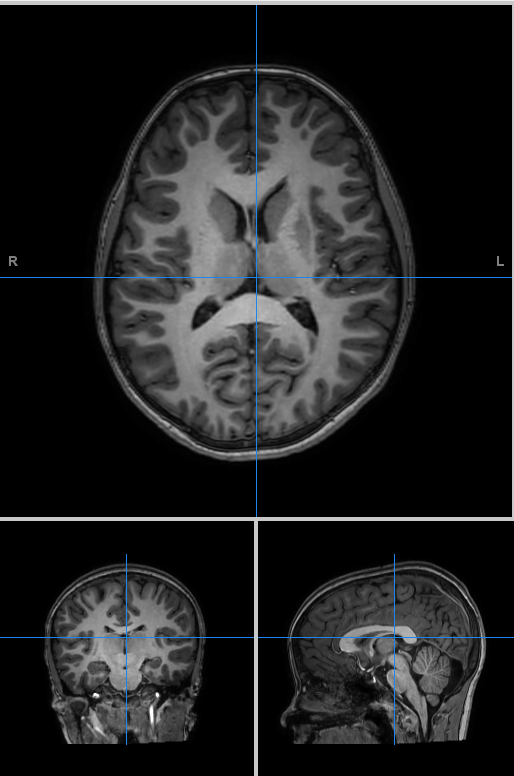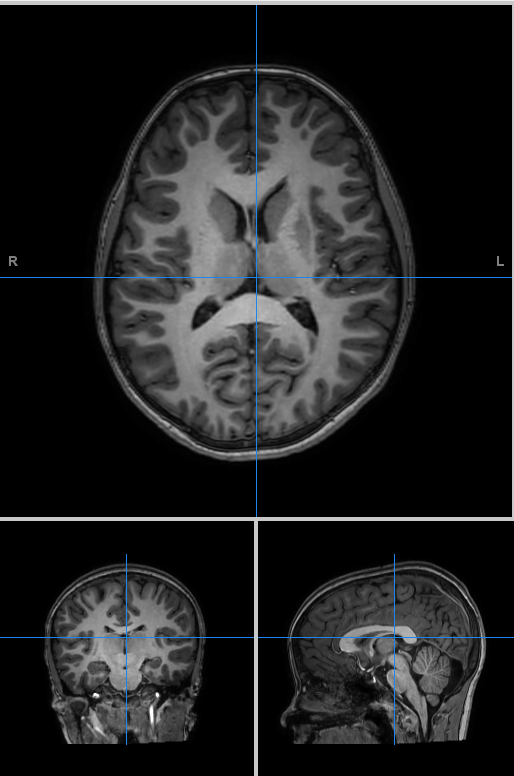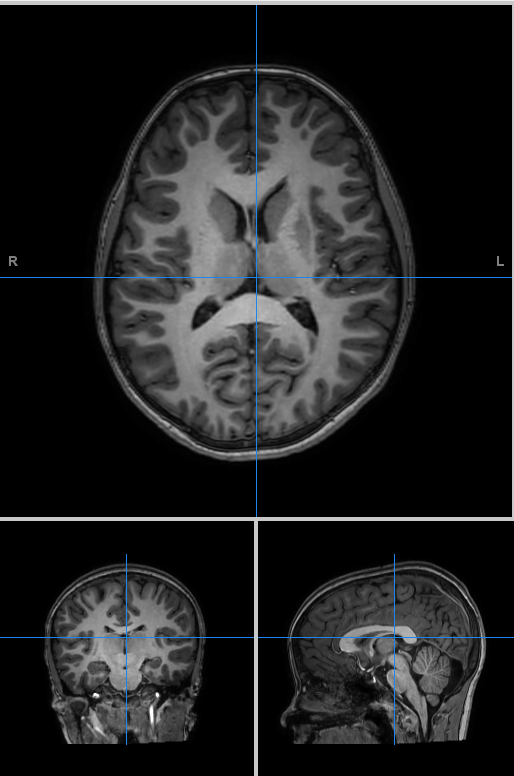 |
| 2(7) | F | R PFC;  MRI negative |  | 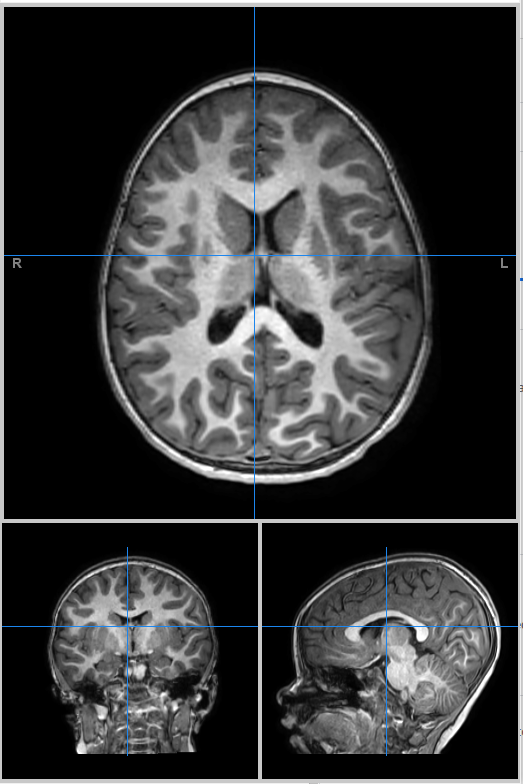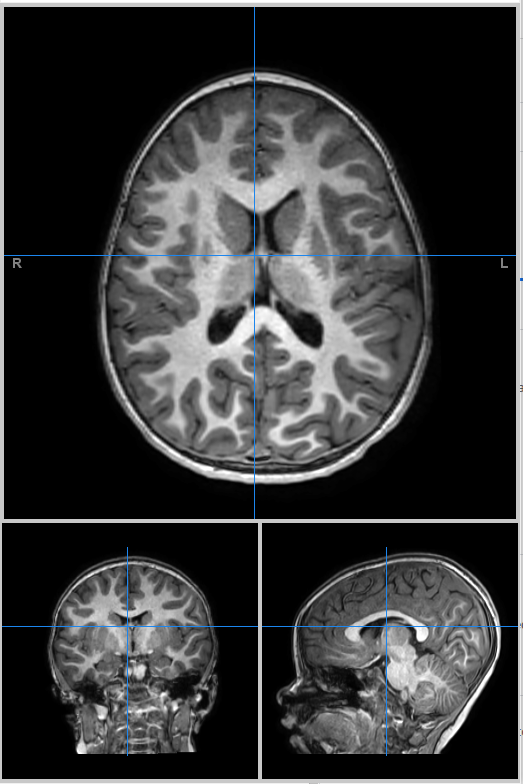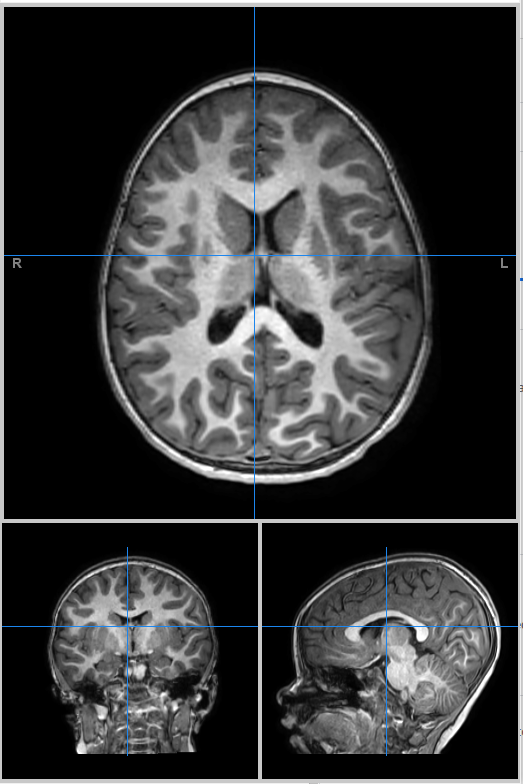 |
| 3(9) | M | R insula, BG, thalamus;  MRI negative |  | 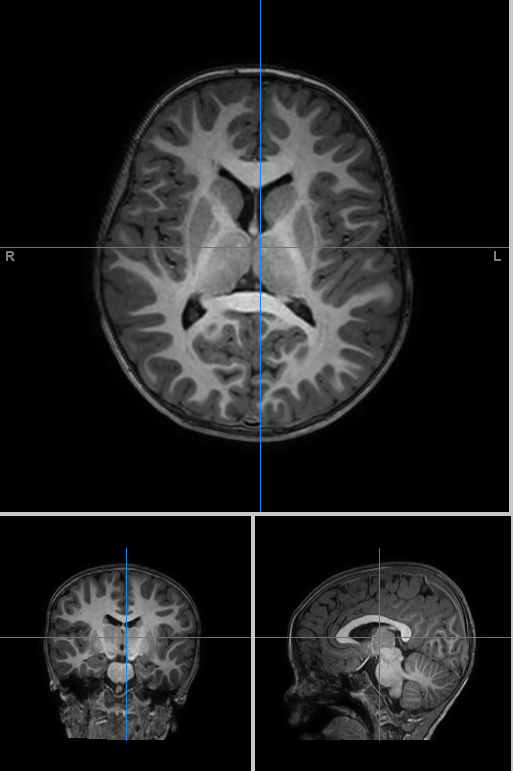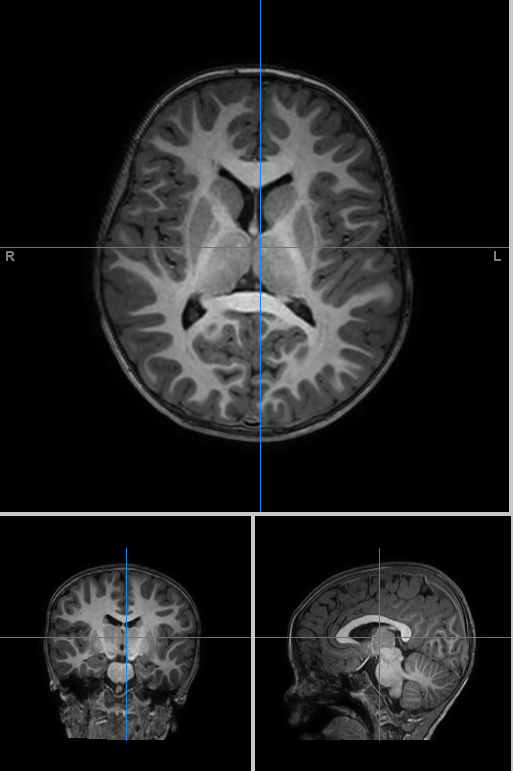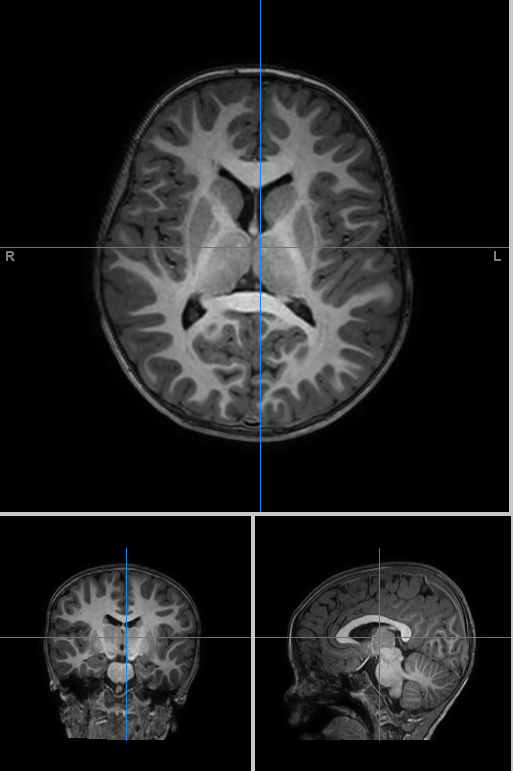 |
| 3(9) | F | R & L P SOZ;  R thalamic groove congenital malformation |  | 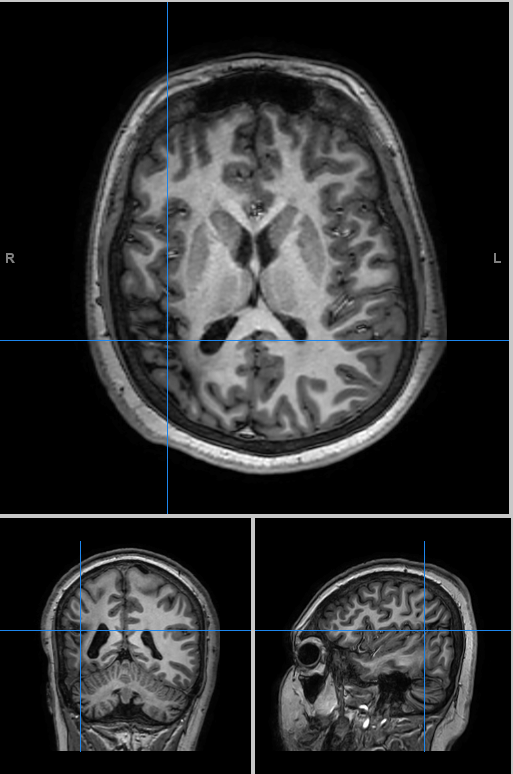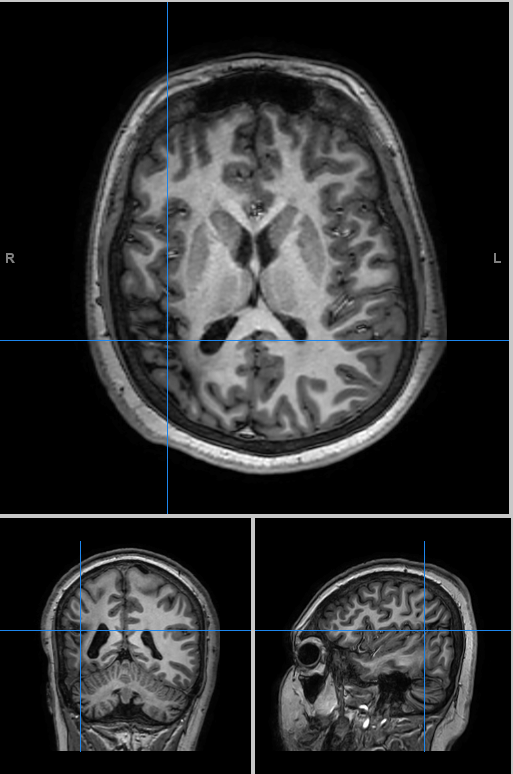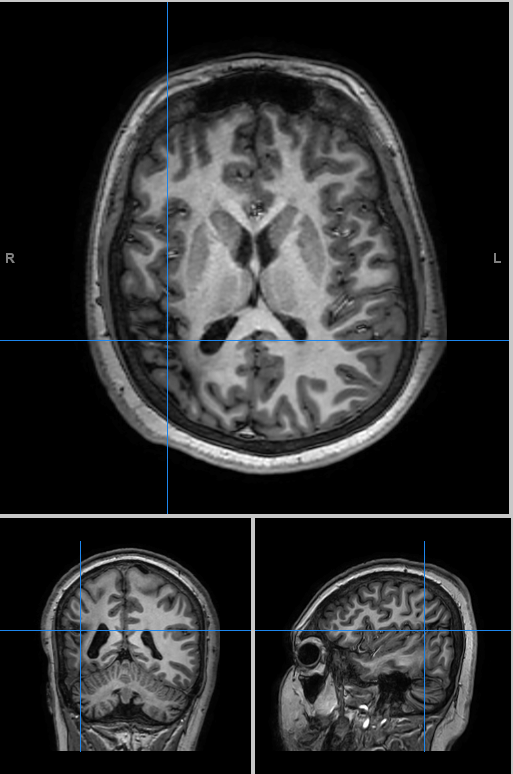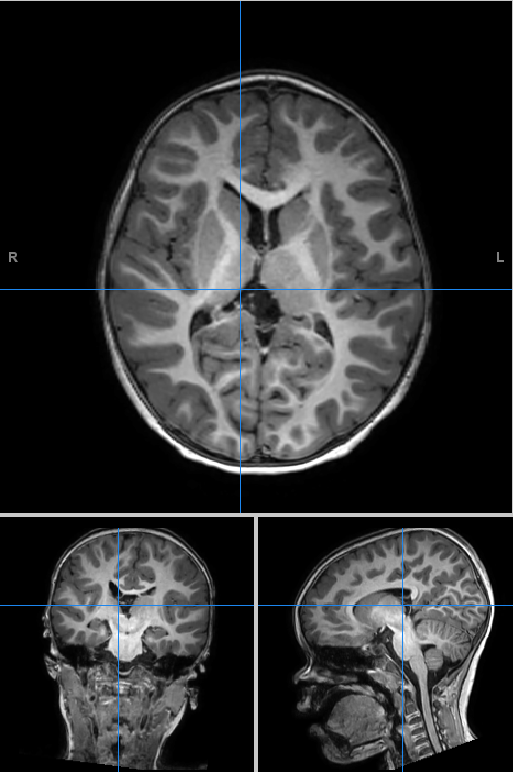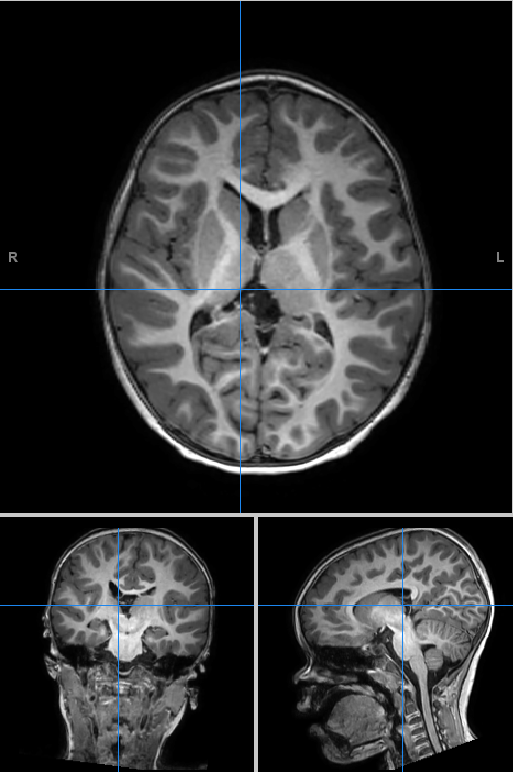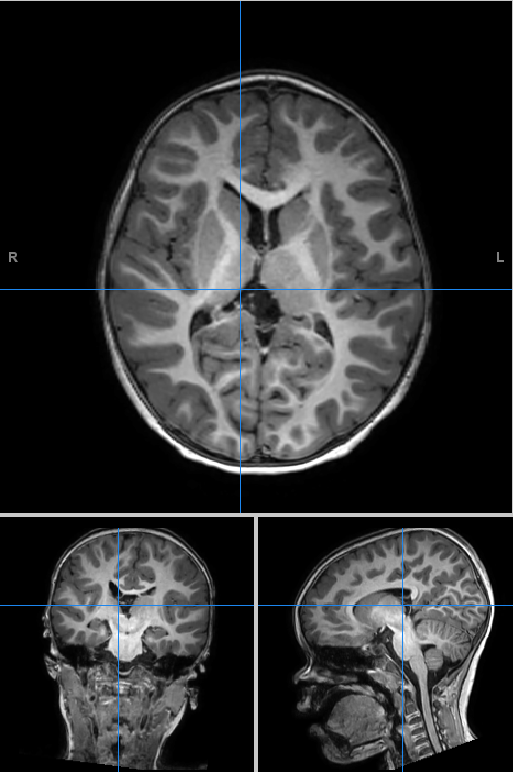 |
| 14(0) | M | R lateral F-P-O SOZ;  R TPO encephalomalacia | 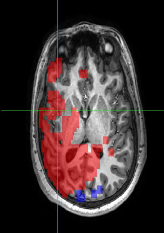 |  |
| 7(11) | M | R MT/AT SOZ;  R posterior colpocephaly and R MT dysplasia |  | 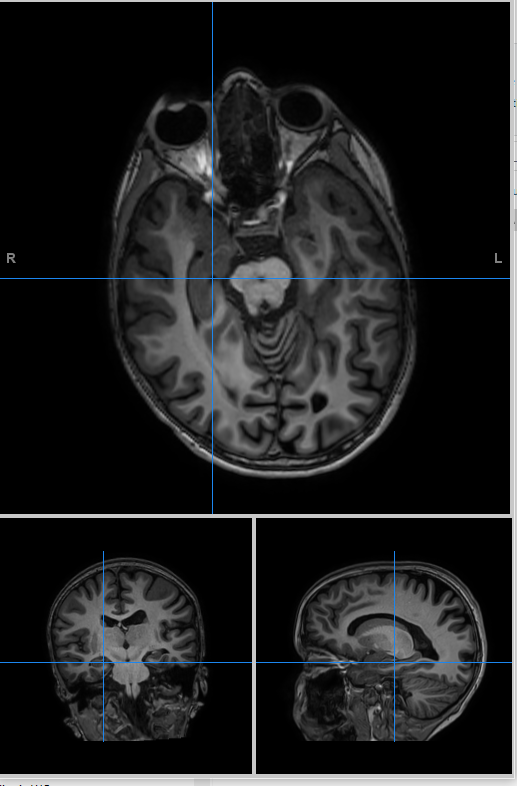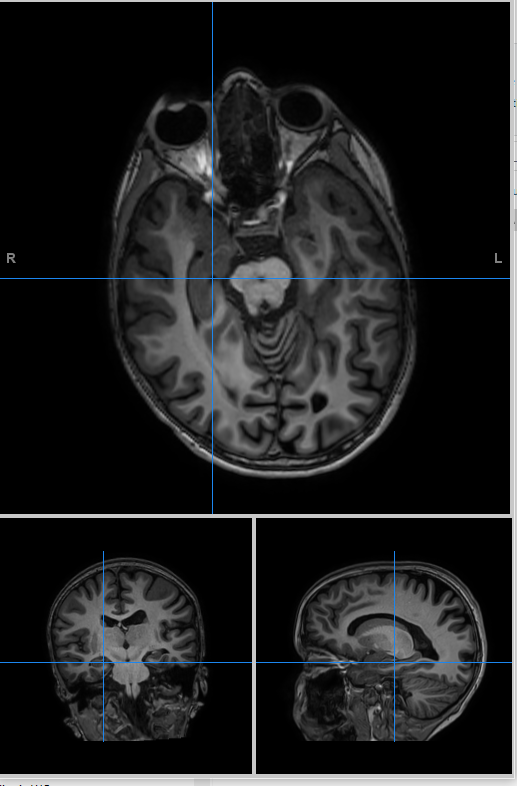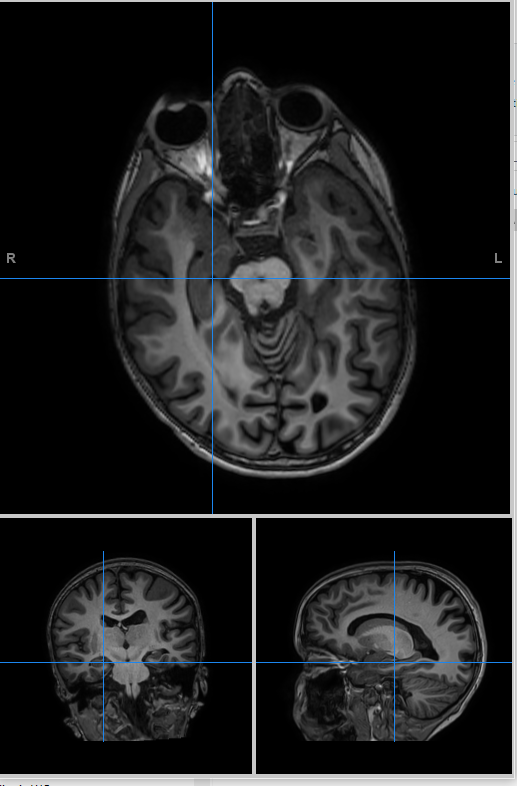 |
| 8(4) | F | R IFG SOZ;  MRI negative |  | 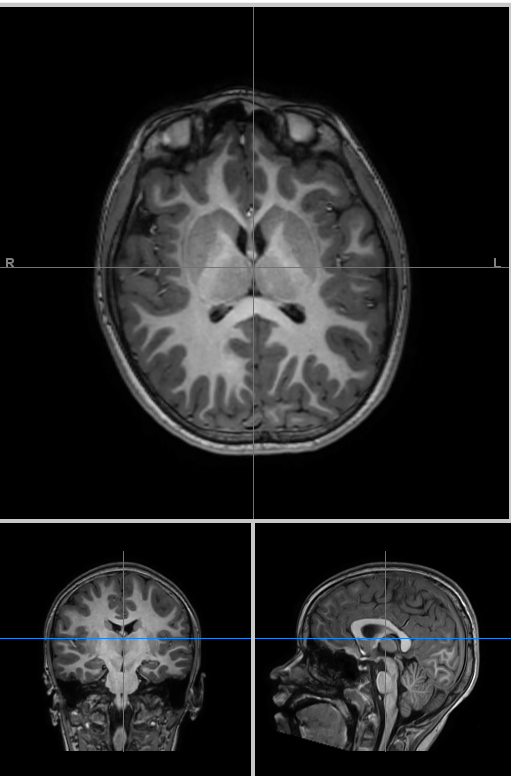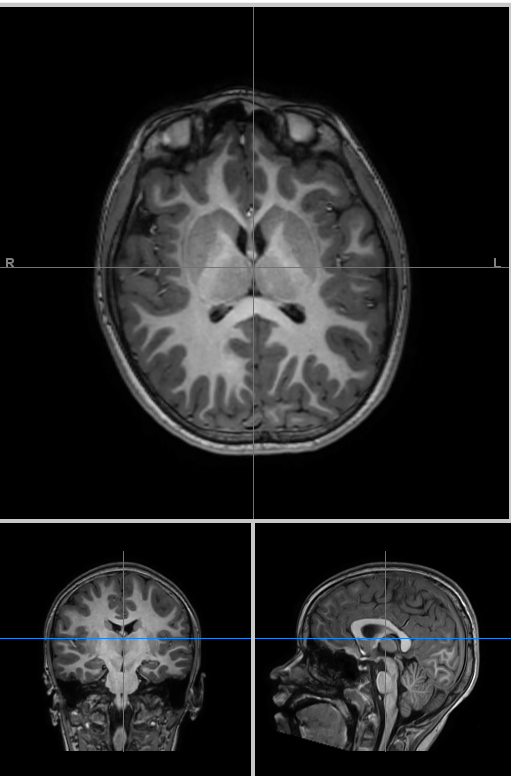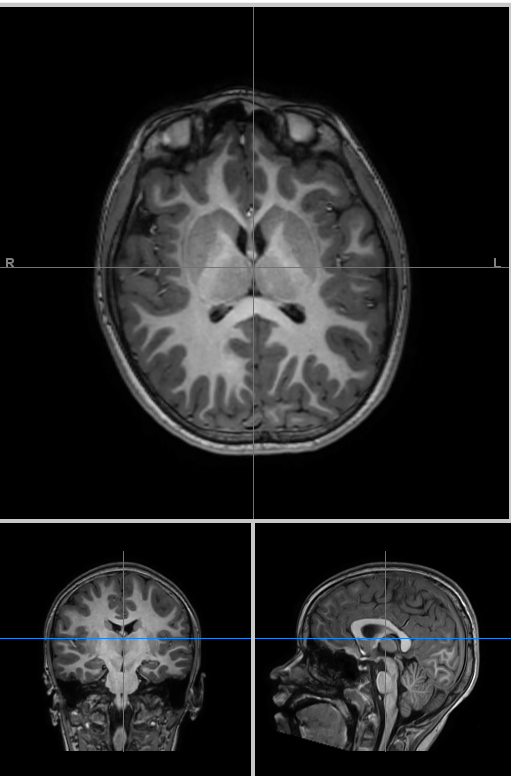 |
| 2(0) | M | R F SOZ;  R F FCD |  | 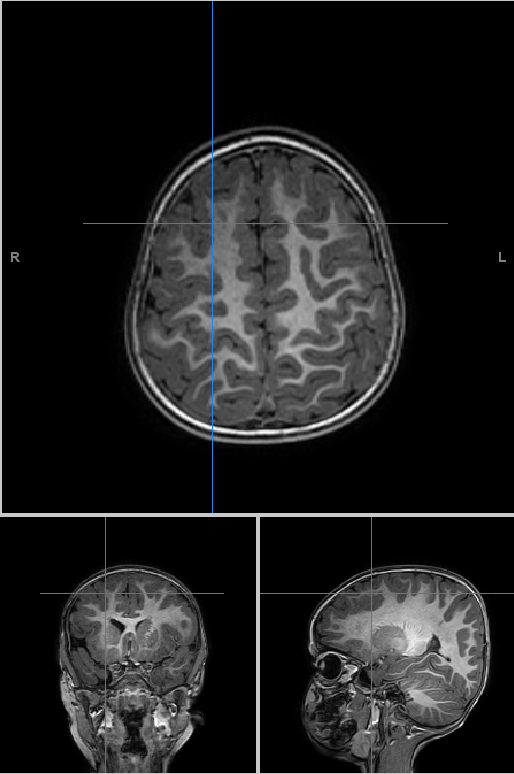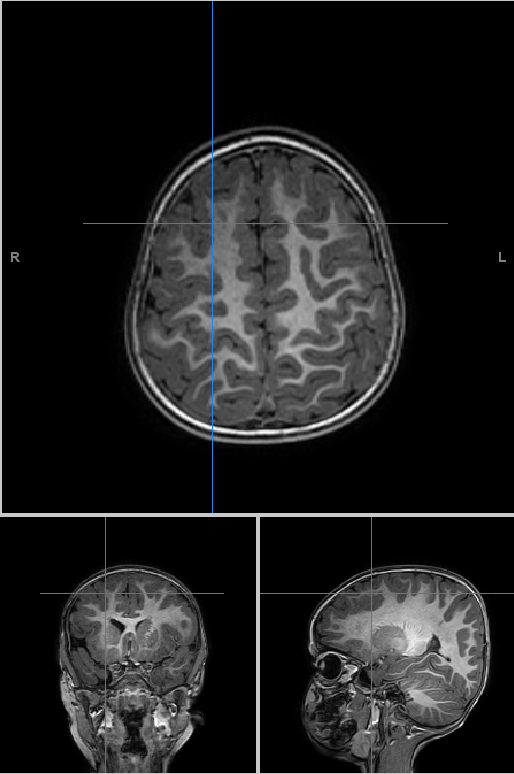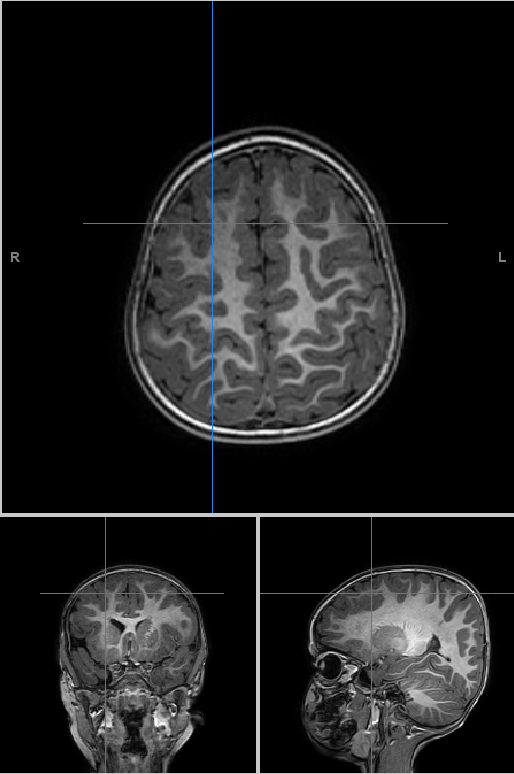 |
| 4(4) | F | R & L F ;  MRI negative |  | 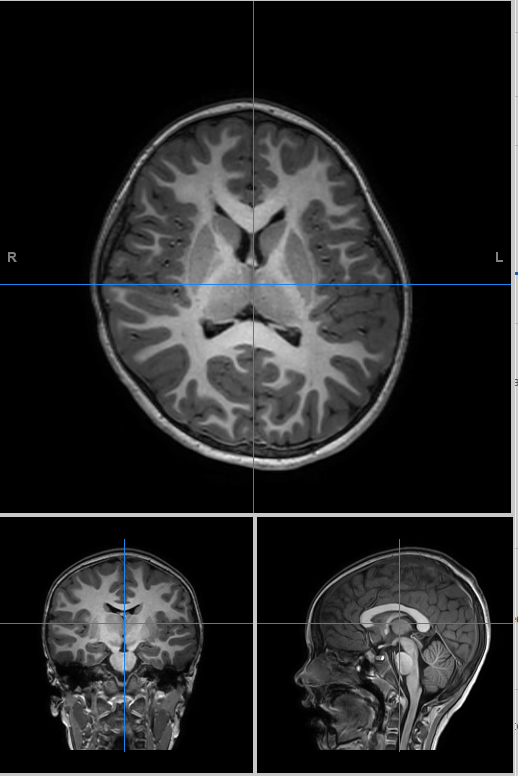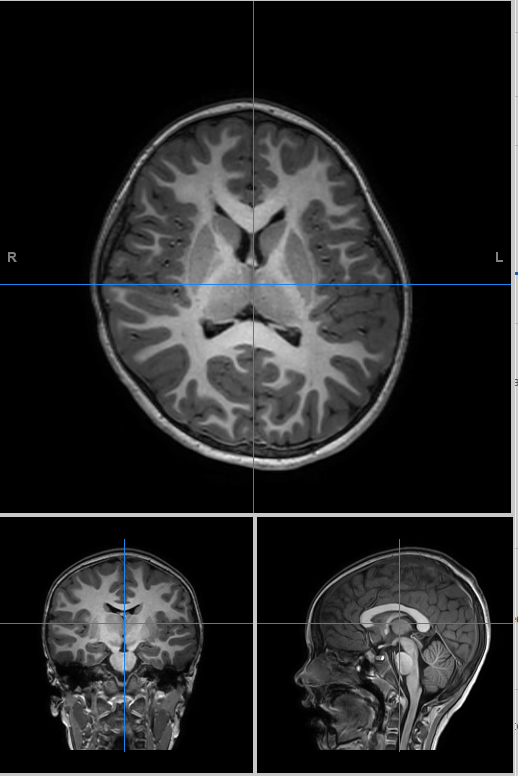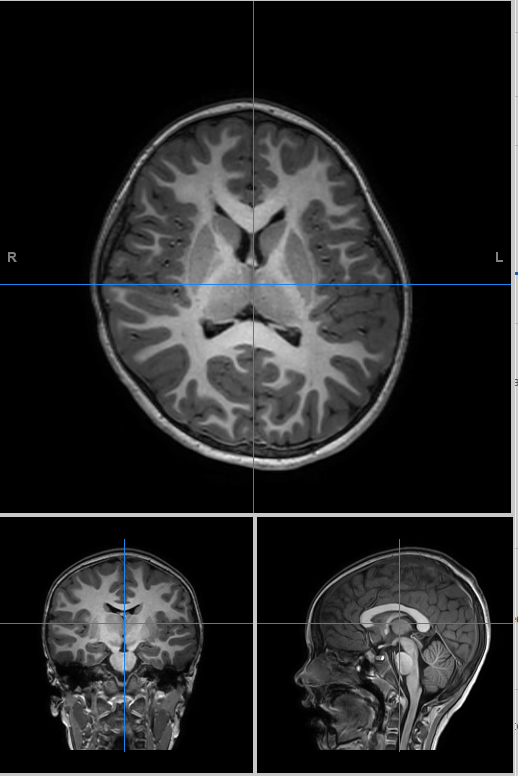 |
| 6(8) | F | B P – centrally; MRI negative |  | 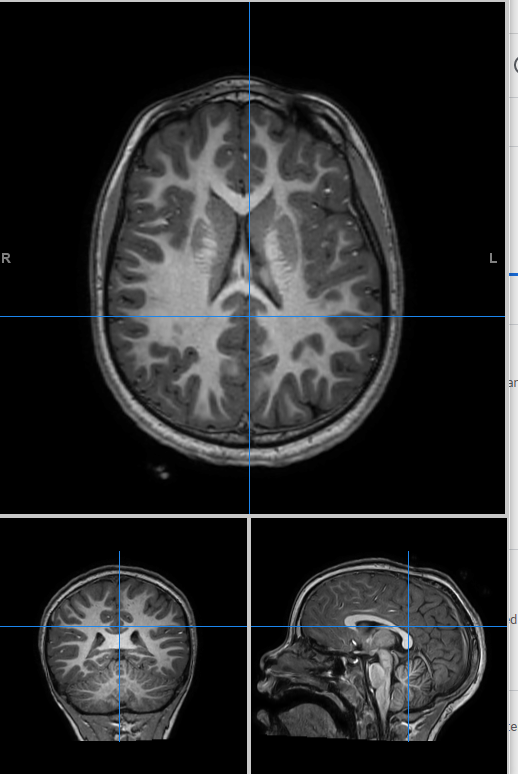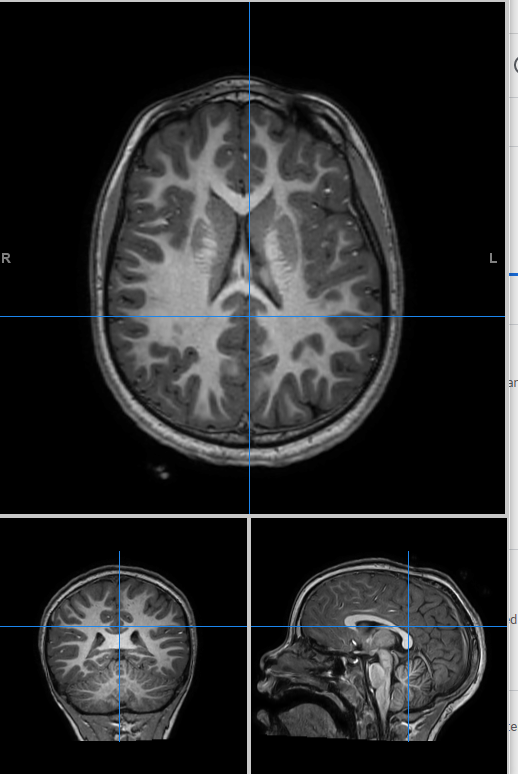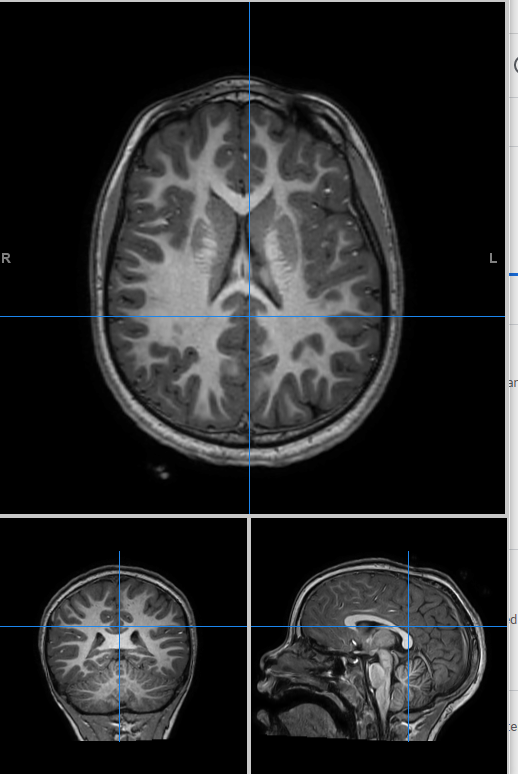 |
| 9(3) | F | R FCD SOZ;  MRI negative |  | 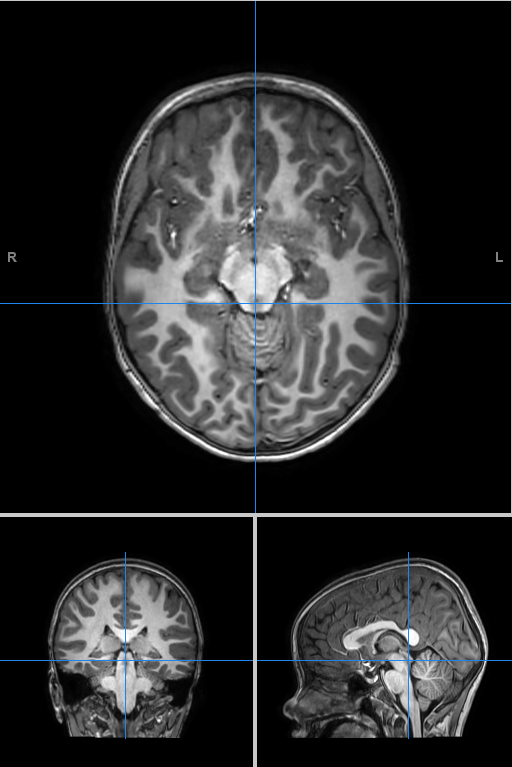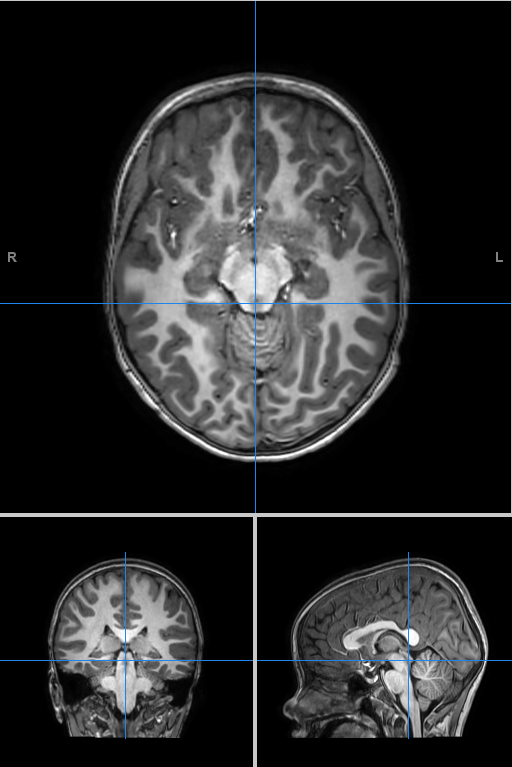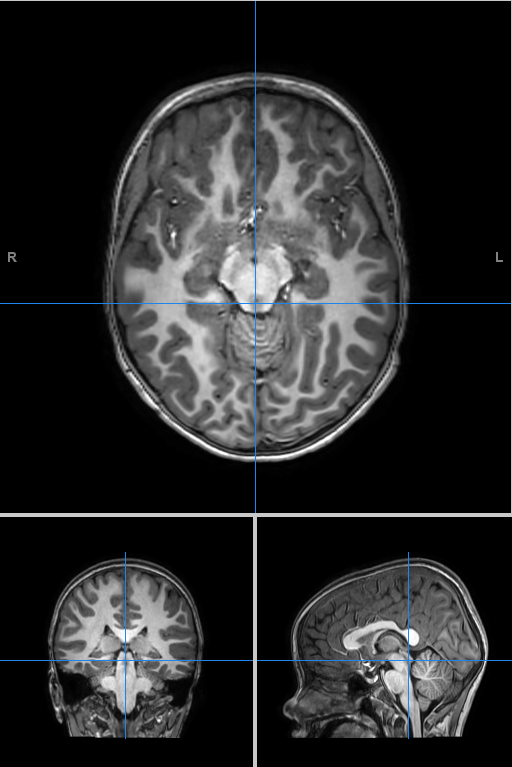 |
