# Supplementary material for: Automated seizure onset zone locator from resting-state functional MRI in drug-resistant epilepsy
Source: Front Neuroimaging. 2023 Jan 4;1:1007668. doi: 10.3389/fnimg.2022.1007668 (PMC10406253; doi:10.3389/fnimg.2022.1007668)
Supplement: Supplementary file 4 [file Table_4.docx]

| 14(8) | M | L & R MT-AT;  L MTS |  |  |
| --- | --- | --- | --- | --- |
| 14(7) | F | midline B R & L F SOZ;  MRI negative |  | 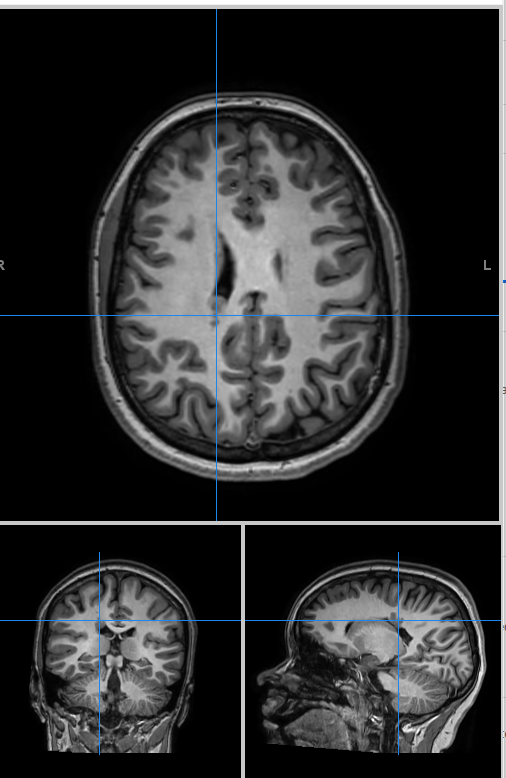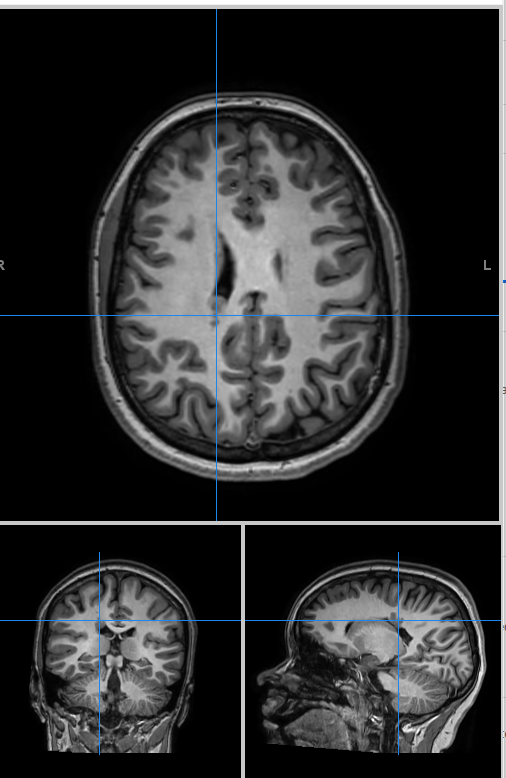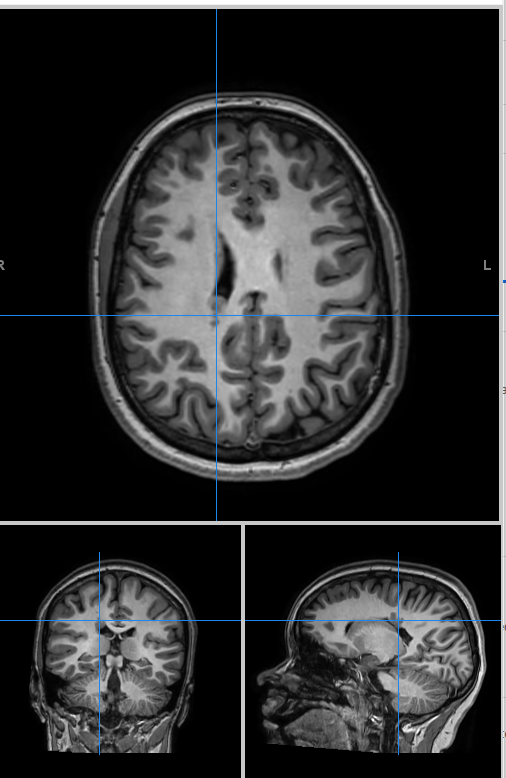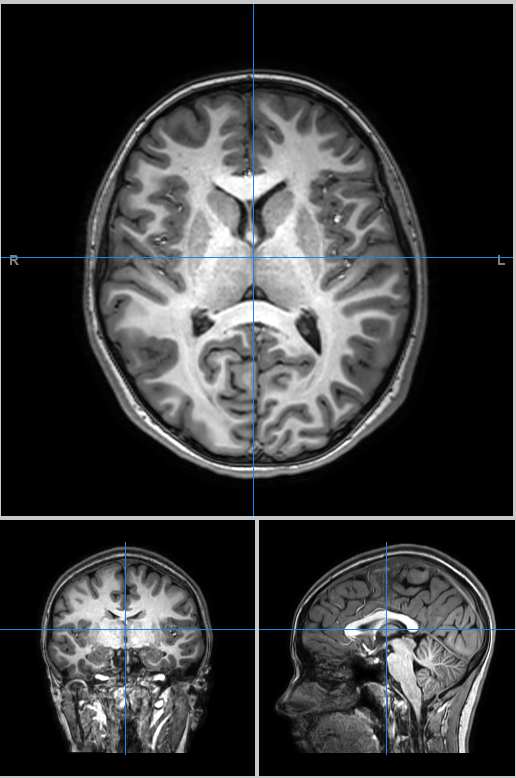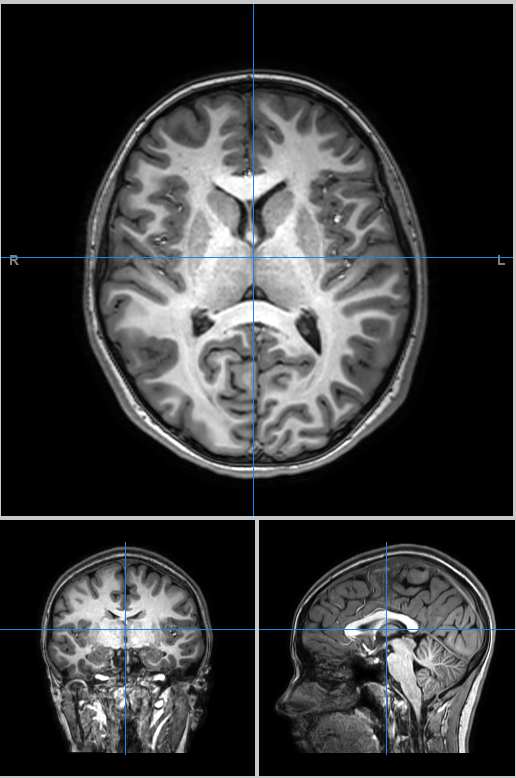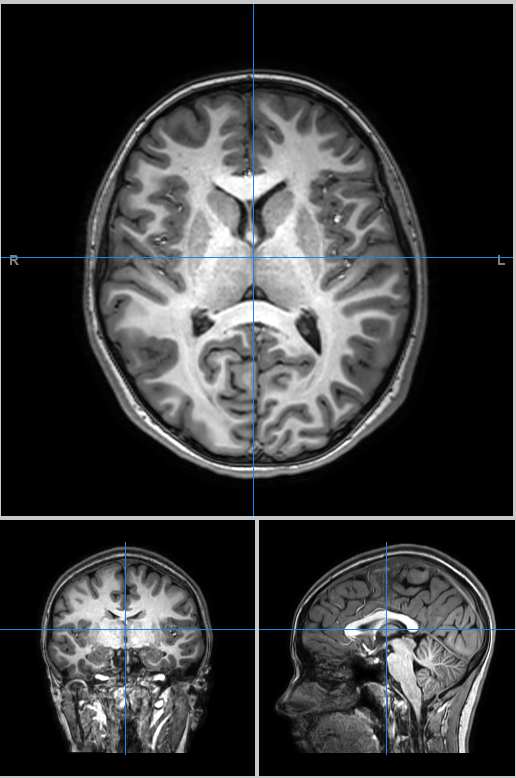 |
| 14(7) | F | cortical region of this right central FP-region gyrus is highly abnormal;  R PFC, L MC  R F periventricular hamartoma | 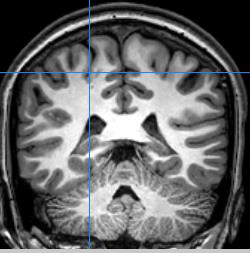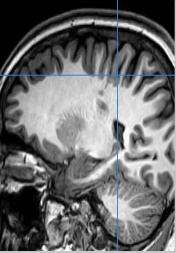  |  |


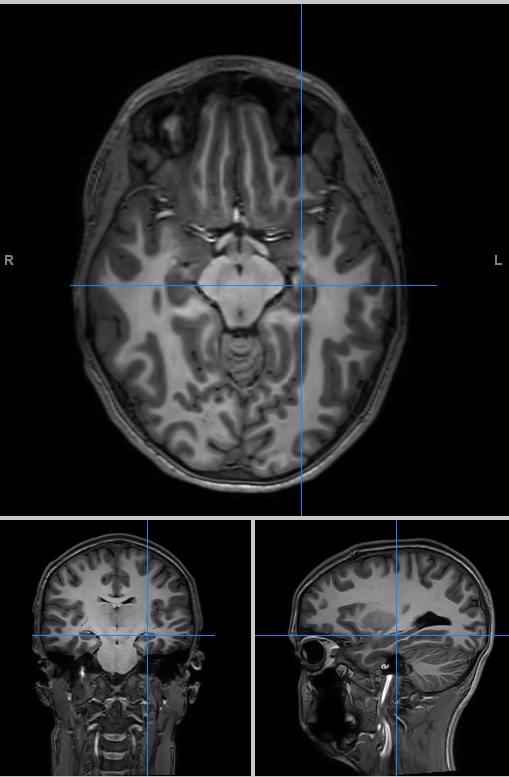

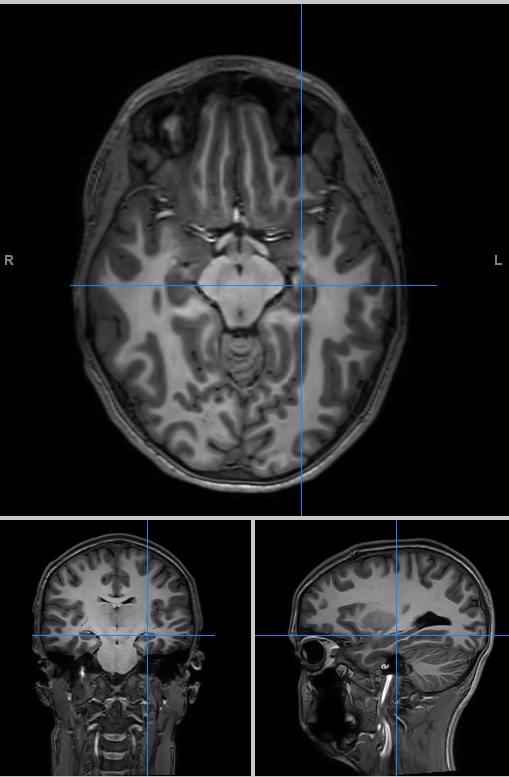

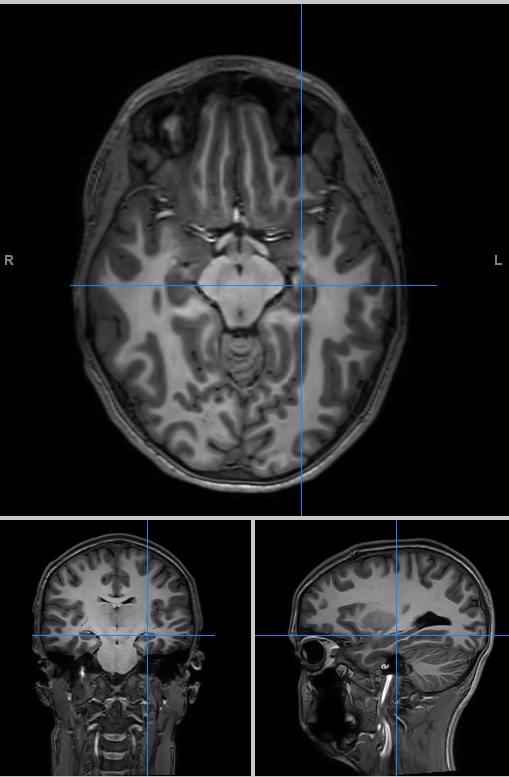


AT anterior temporal; BG basal ganglia; FCD focal cortical dysplasia; F frontal; T temporal, P parietal; O occipital; Opc opercular; SOZ seizure onset zone; L left; R right; B bilateral; MTS mesial temporal sclerosis; MT mesial temporal; IFG inferior frontal gyrus; PFC prefrontal cortex; M male; F female (under sex); WM white matter;
